# Fe-Based Medium-Entropy Alloys: Metastability, Microstructure, Strengthening, and Service-Oriented Design

**DOI:** 10.3390/ma19153205

**Published:** 2026-07-27

**Authors:** Qian Ma, Kun Han, Zhaoyang Wang, Haimei Li, Liangbin Chen, Ran Wei

**Affiliations:** 1 School of Materials Science and Engineering, Zhengzhou University, Zhengzhou 450001, China; 19329793097@163.com; 2 Henan Rongcarbon Technology Co., Ltd., Zhengzhou 450008, China; 18603810087@163.com; 3 Qinyang Hongda Steel Co., Ltd., Jiaozuo 454550, China; 15738593373@163.com; 4 School of Mechatronics Engineering, Xuchang University, Xuchang 461000, China; chenliangbin@xcu.edu.cn

**Keywords:** Fe-based medium-entropy alloys, metastability engineering, heterogeneous structures, precipitation strengthening, transformation-induced plasticity, strength–ductility synergy, service performance, transformation kinetics

## Abstract

Fe-based medium-entropy alloys (MEAs) are a cost-effective class of multi-principal-element alloys with tunable mechanical behavior. Their key advantage lies in the ability of Fe-rich, non-equiatomic compositions to regulate phase stability, deformation behavior, and strain hardening without relying heavily on expensive Co, Ni, or V. Increasing evidence shows that metastable face-centered cubic (FCC) matrices can provide excellent combinations of strength and ductility when their transformation behavior is properly controlled. Through compositional tuning and microstructural regulation, the phase stability, stacking-fault energy, precipitation behavior, and deformation pathways of Fe-based MEAs can be adjusted to achieve a balance between strength, ductility, and service reliability. This review critically synthesizes the metastability, microstructure, strengthening mechanisms, and service-oriented design principles of Fe-based MEAs. The literature discussed in this review was selected from peer-reviewed studies that report clear links among alloy composition, processing history, microstructure, deformation behavior, and mechanical or service-related properties. Unlike reviews that mainly classify alloy systems or deformation modes, this work emphasizes how metastability engineering and microstructural design can be integrated to guide application-specific alloy development. Representative Fe-rich non-equiatomic alloy systems are compared to clarify how alloying and processing regulate metastability, precipitation behavior, transformation kinetics, and strain partitioning. This review highlights that superior properties arise from the coordinated control of metastability, heterogeneous microstructures, and strengthening mechanisms. A central conclusion is that controlled transformation kinetics, rather than the pursuit of a maximum martensite fraction, is the key design variable for sustaining strain hardening and achieving stable strength–ductility synergy. Remaining challenges include quantitative deconvolution of coupled mechanisms, reliable prediction of local metastability, long-term microstructural stability, manufacturability, cost–performance balance, and integration of high-throughput experiments with computational alloy design. Overall, this review provides a service-oriented design framework for high-performance, low-cost Fe-based MEAs through the integrated control of composition, metastability, microstructure, processing, strengthening mechanisms, and application-specific performance.

## 1. Introduction

FCC high-entropy alloys (HEAs)/MEAs offer high ductility, fracture resistance, and cryogenic strength, but many early systems rely on costly Co, Ni, or V. Fe-based MEAs occupy a practical position among HEAs/MEAs, which cut costs by using Fe as the primary element while preserving broad composition-microstructure tunability [1]. Their distinction from conventional steels and equiatomic HEAs lies in non-equiatomic control of phase stability, deformation mode, and strain hardening. Recent reviews have identified DIMT, especially the FCC→BCC pathway, as an effective route to mitigate the strength–ductility trade-off [2,3].

Although recent reviews have summarized deformation-induced martensitic transformation, metastability engineering, and mechanical behavior in high- and medium-entropy alloys, most of them mainly focus on deformation mechanisms, alloy classification, or general strength–ductility relationships. In contrast, this review focuses specifically on Fe-rich medium-entropy alloys from the perspective of cost-performance design and service-oriented alloy development. The novelty of this review lies in integrating four aspects that are usually discussed separately: Fe-rich non-equiatomic composition design, metastability and transformation kinetics, microstructural regulation across multiple length scales, and service qualification under practical application conditions. Rather than treating martensitic transformation simply as a strengthening mechanism, this review emphasizes that controlled transformation kinetics, strain partitioning, and microstructural stability are central to achieving reliable performance.

**Figure 1:**
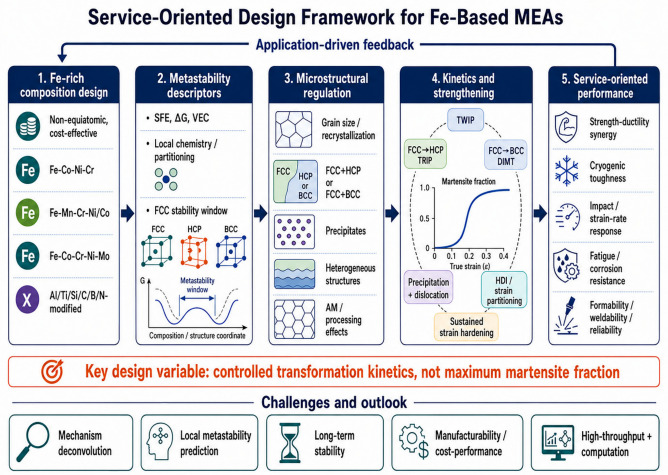
Service-oriented design framework for Fe-based medium-entropy alloys (MEAs), linking Fe-rich composition design, metastability descriptors, microstructural regulation, transformation kinetics/strengthening mechanisms, and service-oriented performance. The framework emphasizes that controlled transformation kinetics, rather than the maximum martensite fraction, is the key design variable for sustaining strain hardening and achieving strength–ductility synergy, cryogenic toughness, fatigue/corrosion resistance, impact response, formability, weldability, and reliability. Major challenges include mechanism deconvolution, local metastability prediction, long-term stability, manufacturability, cost–performance balance, and high-throughput computational alloy design.

The scope and structure of this review are summarized in Figure 1. First, the compositional characteristics and metastability criteria of Fe-based MEAs are discussed, with emphasis on Fe-rich non-equiatomic design, stacking-fault energy, Gibbs free-energy difference, and phase-stability descriptors. Second, representative alloy systems are classified according to their dominant design routes, including Fe-Co-Ni-Cr, Fe-Mn-Cr-Ni/Co, Fe-Co-Cr-Ni-Mo, and Al/Ti/Si/interstitial-modified systems. Third, microstructural regulation strategies, such as partial recrystallization, dual-phase design, precipitation strengthening, heterogeneous structures, gradient layers, additive manufacturing, and deformation processing, are compared in terms of transformation kinetics and strain partitioning. Finally, this review connects these mechanisms with service-oriented requirements, including cryogenic performance, fatigue resistance, corrosion behavior, formability, weldability, irradiation tolerance, and long-term reliability.

In Fe-based MEAs, the FCC matrix is often most useful when deliberately metastable. TWIP, FCC→HCP TRIP, and FCC→BCC DIMT can sustain strain hardening when the transformation pathway is properly regulated. Mn-dependent Fe-Mn-Co-Cr alloys and Fe-rich Fe-Co-Ni-Cr alloys demonstrate that metastability is a tunable parameter governing the dominant deformation pathway [4,5].

FCC stability depends on coupled thermodynamic and local-chemical factors, including SFE, Gibbs free-energy difference, valence electron concentration, lattice distortion, and matrix chemistry [3]. Mn/Ni and Co/Ni substitution studies further show that alloying can regulate the FCC-HCP Gibbs free-energy difference, SFE, and initial phase constitution [6,7]. However, excessive or spatially localized transformation can impair ductility, whereas grain refinement and strain-dependent martensite evolution can distribute transformation over a broader strain range [8,9]. Therefore, the key design target is not to maximize the final martensite fraction, but to regulate transformation kinetics so that martensite formation proceeds progressively during deformation and continuously sustains strain hardening.

Representative Fe-based MEAs can be classified according to their dominant composition-design routes. Fe-Co-Ni-Cr-based systems mainly regulate FCC→BCC transformation through the Fe/(Co, Ni) balance and phase-stress partitioning [5,10]. Fe-Mn-Cr-Ni/Co-based systems are governed mainly by SFE and can balance slip, TWIP, and FCC→HCP TRIP [11,12]. Mo-bearing Fe-Co-Cr-Ni-Mo alloys introduce solute strengthening, μ-phase/carbide precipitation, and partitioning control [13,14,15]. Al, Ti, Si, and interstitial C/B/N additions further broaden the design space. Al drives FCC alloys toward FCC + BCC [16], whereas Si lowers Co/Ni content and promotes Lüders deformation, DIMT, and HDI strengthening [17]. C additions can generate carbide precipitation, interstitial strengthening, or twinning-dominant FCC deformation depending on matrix chemistry and processing [12,18]. Heavy N-doped FeMnCoCr combines nitride precipitation, martensite, heterogeneous grains, HDI strengthening, and twinning-dominant deformation [19]. N-supersaturated Fe_50_Mn_30_Co_10_Cr_10_ coatings instead follow a solid-solution route without nitride formation [20]. Trace-B-doped Fe_50_Mn_30_Co_10_Cr_10_ is a solid-solution B and sequential TWIP/TRIP case, not a boride-precipitation case [21]. FeMnCoCrCuC and Nb-added FeMnCoCrC MEAs represent M_23_C_6_ dual-heterostructure and Nb_2_C/carbide-morphology routes [22,23]. In Cr_12_Fe_60_Mn_16_Ni_12_ MEAs, small Al or C additions increase yield strength (YS) through solute, precipitate, and lattice-strain strengthening [24]. Ti-related alloying and particle-reinforcement strategies further diversify the microstructural design of Fe-based MEAs. Depending on the alloying and processing route, these strategies may couple retained FCC, thermally or deformation-induced BCC martensite, μ-phase precipitation, deformation twins, dislocations, microbands, nanoscale carbides, or TiC-related phase-stability tuning [25,26,27]. In particular, TiC-reinforced additively manufactured composites show that partial TiC decomposition can stabilize γ-austenite and suppress DIMT, shifting deformation toward slip-dominated plasticity [27].

Microstructure provides the second lever for controlling metastability. Composition sets the thermodynamic tendency, whereas grain size, recrystallization state, retained defects, precipitates, shear bands, twins, cell boundaries, and gradient layers determine martensite nucleation and transformation kinetics [3]. Partial recrystallization and bimodal structures can regulate DIMT and hardening contributions across different FCC domains [28,29], while additive manufacturing introduces cell-boundary segregation and defect structures that tune local FCC stability and room-temperature TRIP [30].

Strengthening in Fe-based MEAs is intrinsically coupled. Solid-solution atoms, grain boundaries, stored dislocations, deformation substructures, and multiphase precipitates can jointly impede dislocation motion and improve strength [31]. TRIP and TWIP sustain hardening through new interfaces and slip-path subdivision; HDI and back-stress strengthening arise from incompatible deformation between domains [32]. Representative heterogeneous, precipitation-strengthened, and transformation-assisted Fe-based MEAs show that the strength–ductility balance should be interpreted as an evolving structure–mechanism–property relationship rather than as the contribution of a single strengthening mechanism [14,33,34].

Several Fe-based MEAs show strong low-temperature responses because cryogenic deformation can enhance transformation-mediated hardening and phase-stress partitioning [5,11,17,25,33,35]. Tensile strength alone does not define engineering usefulness. Cryogenic toughness, fatigue resistance, corrosion behavior, strain-rate response, formability, weldability, and long-term reliability do not necessarily scale with tensile strength [36,37]. Corrosion behavior further confirms that service performance cannot be inferred from tensile strength alone. Reported comparisons involving Co-for-Ni substitution, subsystem alloys, and Ti/Si-modified Fe-based MEAs show that corrosion resistance is strongly affected by alloy chemistry, phase constitution, and passive-film characteristics rather than by tensile strength alone [7,12,38,39]. Therefore, Fe-based MEAs should be evaluated as service materials rather than only as tensile specimens.

Overall, this review establishes a service-oriented framework for Fe-based MEAs by linking Fe-rich cost-performance alloy design, metastability kinetics, microstructural regulation, strengthening mechanisms, and service qualification.

## 2. Thermodynamic and Microstructural Basis of Metastability in Fe-Based MEAs

### 2.1. Metastability and Phase Stability

In Fe-based MEAs, metastability is an alloy-design variable rather than a constitutional deficiency. It describes a state in which the FCC matrix is sufficiently stable for processing and early plastic deformation, but sufficiently unstable to activate TWIP, FCC→HCP TRIP, or FCC→BCC DIMT under appropriate loading conditions. Therefore, phase stability should be evaluated not only by whether martensite can form, but also by when, where, and how gradually the transformation proceeds during deformation. 

In this context, metastability should be considered at two different levels. Thermodynamic or chemical criteria, such as stacking-fault energy (SFE), Gibbs free-energy difference, valence electron concentration (VEC), phase constitution, and elemental partitioning, define the intrinsic tendency of the FCC matrix to remain stable or transform into HCP or BCC martensite. In contrast, kinetic and microstructural factors, including grain size, retained defects, shear bands, twins, precipitates, local segregation, cell boundaries, and gradient interfaces, determine where, when, and how fast the transformation occurs during deformation. Therefore, phase stability in Fe-based MEAs should be evaluated by combining thermodynamic descriptors with transformation kinetics and microstructural constraints. To clarify the conceptual relationship among SFE, phase stability, deformation mode, and potential failure risk, a qualitative design map is summarized in Table 1.

**Figure 2:**
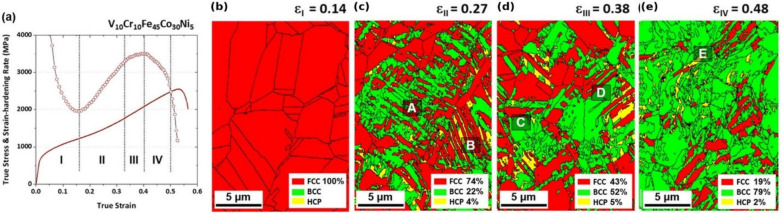
Stage-dependent cryogenic strain hardening and martensitic transformation in the V_10_Cr_10_Fe_45_Co_30_Ni_5_ medium-entropy alloy. (**a**) True stress and strain-hardening rate as functions of true strain, showing four characteristic deformation stages. (**b**–**e**) EBSD phase maps obtained at true strains of 0.14, 0.27, 0.38, and 0.48, respectively, showing the progressive reduction in the FCC fraction and the corresponding formation and evolution of BCC martensite. In (**c**), regions A and B indicate a complex BCC network containing abundant high-angle grain/phase boundaries and BCC formed along pre-existing HCP regions, respectively. In (**d**), regions C and D indicate the growth and coalescence of BCC martensite, whereas region E in (**e**) highlights the deformation and bending of the transformed BCC domains. The increase in strain-hardening rate is associated with BCC martensite formation and the multiplication of grain and phase boundaries, whereas the late-stage decrease is related to the growth, coalescence, deformation, and bending of the transformed BCC domains. Adapted from Fig. 5(a,b,d,f,h) of Jo et al. [41], *Scientific Reports* 9, 2948 (2019), under the Creative Commons Attribution 4.0 International License. The original panels were rearranged, relabeled, and graphically modified.

Based on this framework, specific Fe-based MEA systems can be interpreted as examples of controlled destabilization rather than simple phase instability. Fe-Co-Ni-Cr ferrous MEAs exhibit composition-dependent phase instability. In Fe_x_(CoNi)_90−x_Cr_10_, lowering the Co-Ni content reduces FCC stability, enabling Fe_60_Co_15_Ni_15_Cr_10_ to undergo FCC→BCC transformation during cryogenic deformation [5]. Although the starting microstructure is nearly single-phase FCC, sequential BCC martensite forms under strain, sustains multi-stage hardening, and produces ~1.5 GPa tensile strength and ~88% ductility at 77 K [5]. Thus, the parent FCC phase can transform to either HCP or BCC, depending on composition and temperature, leading to distinct hardening behaviors. The Fe_80−x_Mn_x_Co_10_Cr_10_ HEA system exemplifies this: Fe_50_Mn_30_Co_10_Cr_10_ retains ~28% HCP after quenching, with further FCC→HCP transformation under tensile loading [4]. This shifts design from phase stabilization to controlled destabilization, leveraging reduced stability for phase-boundary and transformation-induced hardening [4]. The kinetic contribution of FCC→BCC transformation is more directly illustrated by the cryogenic deformation of the V_10_Cr_10_Fe_45_Co_30_Ni_5_ MEA. As shown in Figure 2, this alloy exhibits a four-stage strain-hardening response associated with the progressive evolution of deformation-induced martensite. At a true strain of 0.14, the EBSD phase map remains nominally fully FCC, indicating that the transformation has not yet been extensively activated at the EBSD-resolvable scale. At a true strain of 0.27, the FCC fraction decreases to 74%, accompanied by the formation of 22% BCC and 4% HCP phases. The emerging BCC network and the accompanying increase in high-angle grain boundaries and phase boundaries coincide with the rapid increase in strain-hardening rate. With further deformation to a true strain of 0.38, the BCC fraction increases to 52%, while the FCC fraction decreases to 43%. Although transformation continues, the growth and coalescence of the BCC domains reduce the incremental boundary-strengthening contribution. At a true strain of 0.48, the microstructure becomes BCC-dominated, containing approximately 79% BCC, 19% FCC, and 2% HCP, and the deformation and bending of the transformed BCC domains are accompanied by a reduction in strain-hardening rate [41].

**Figure 3:**
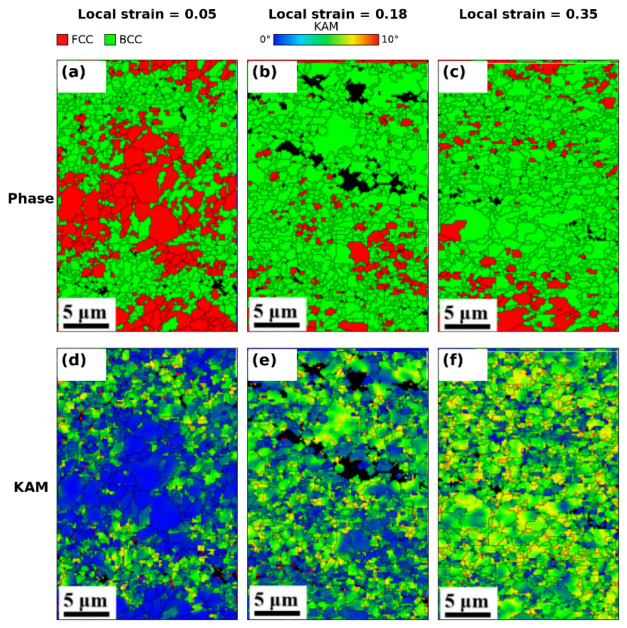
Spatially resolved FCC→BCC transformation and strain accumulation in the SMC30 ferrous medium-entropy alloy at 298 K. (**a**–**c**) EBSD phase maps at local strains of 0.05, 0.18, and 0.35, respectively, showing progressive transformation of FCC into BCC martensite across the compositionally heterogeneous MEA and transition regions. (**d**–**f**) Corresponding kernel average misorientation maps, showing increased local lattice rotation and dislocation accumulation after the transformation rate decreases. The results illustrate a transition from TRIP-dominated deformation at low and intermediate strains to dislocation-dominated hardening at larger strain. Adapted from Son et al. [42], Microstructures 6, 2026066 (2026), https://doi.org/10.20517/microstructures.2025.117, licensed under the Creative Commons Attribution 4.0 International License. Changes were made.

These results demonstrate that the mechanical benefit of martensitic transformation is governed not only by the final martensite fraction, but also by the transformation onset strain, transformation rate, evolving morphology, and strain interval over which the transformation proceeds. During the early stage, the parent FCC phase accommodates plastic strain while retaining a sufficient transformable fraction. The subsequent formation of BCC martensite introduces new phase boundaries and dynamically subdivides the microstructure, thereby increasing the strain-hardening rate. However, when the transformed BCC domains grow and coalesce, the rate of new-interface generation decreases, and the incremental contribution of transformation to strain hardening is progressively weakened. The useful transformation state is therefore not simply one with a high BCC fraction, but one in which martensite formation remains progressive over a sufficiently broad strain range. The kinetics of martensitic transformation can be regulated not only along the strain axis but also spatially through compositional heterogeneity. A recent spatial metastability control strategy introduced pure Fe into a metastable Fe_60_Co_15_Cr_10_Ni_15_ ferrous MEA, producing Fe-rich regions, MEA regions, and compositionally graded transition regions with different FCC stabilities. As shown in Figure 3, the SMC30 alloy already exhibits pronounced FCC→BCC transformation in both the transition and MEA regions at a local strain of 0.05. At a local strain of 0.18, corresponding approximately to the peak strain-hardening rate, most of the FCC phase in the MEA regions has transformed into BCC martensite. With further deformation to a local strain of 0.35, the BCC fraction increases only slightly, whereas the kernel average misorientation rises substantially, indicating that the dominant hardening mechanism shifts from transformation-assisted plasticity to dislocation accumulation [42].

Figure 3 further demonstrates that beneficial metastability can be spatially programmed. The compositionally graded transition regions reduce local FCC stability and activate transformation earlier than the relatively stable MEA regions, thereby distributing martensitic transformation across different locations and deformation stages. Once most of the transformable FCC has been consumed, however, further plastic deformation is accommodated mainly by dislocation accumulation rather than continued phase transformation. This observation reinforces that the final martensite fraction alone is insufficient for evaluating TRIP efficiency. The transformation onset, spatial sequence, transformation rate, and remaining transformable FCC fraction must be considered together. Therefore, reduced phase stability should not be interpreted as universally beneficial. Beneficial metastability requires delayed, spatially distributed, and gradual transformation during deformation, so that martensite formation continuously supplies new interfaces, promotes strain partitioning, and sustains work hardening. In contrast, harmful metastability occurs when the parent FCC phase transforms too early, too rapidly, or too locally. Such abrupt transformation can rapidly exhaust the transformable FCC fraction, concentrate strain within transformed regions, reduce the capacity for subsequent strain hardening, and promote premature necking or fracture. Thus, the design objective is not to reduce FCC stability as much as possible, but to place the alloy within a metastability window in which transformation is sufficiently active to enhance strain hardening while remaining sufficiently delayed and distributed to preserve ductility. For the FCC→BCC route, in situ neutron diffraction provides direct mechanical evidence: in Fe_60_Co_15_Ni_15_Cr_10_, the BCC fraction increases during low-temperature tension, while the stress carried by BCC martensite rises continuously as the FCC contribution decreases [10]. Choi et al. showed that BCC-DIMT can enhance hardening but also trigger early necking under uniaxial tension when transformation is excessive or spatially non-uniform, especially in the less stable Fe_64_(CoNi)_26_Cr_10_ alloy [40]. Under biaxial stretching, martensite-assisted strain accommodation can instead increase deformation resistance [40].

### 2.2. Composition-Dependent Tuning of SFE and Transformation Tendency

Following the conceptual map in Table 1, composition tunes metastability beyond conventional solute strengthening, but the effect of each alloying element should be interpreted as matrix- and processing-dependent rather than universal. The Mn-Ni balance is representative: in Fe_45_Co_30_Cr_10_V_10_Ni_5−x_Mn_x_, replacing Ni with Mn lowers the Gibbs free-energy difference between FCC and HCP and decreases the calculated SFE [6]. The deformation pathway changes accordingly; the 5Mn alloy develops extensive BCC and HCP martensite at cryogenic temperature while reaching tensile strength above 1.6 GPa [6]. A related stability shift occurs when Co replaces Ni in Fe_50_Mn_25_Cr_15_Ni_10−x_Co_x_: Ni_10_ remains single-FCC, Ni_5_Co_5_ contains a limited HCP fraction, and Co10 becomes HCP-rich [7]. This confirms the opposing roles of Ni and Co: Ni stabilizes FCC, whereas Co lowers SFE and promotes FCC→HCP transformation [7]. At 77 K, Ni10 retains the best ductility and strength–ductility product, whereas Co_10_ loses ductility because its HCP-rich initial constitution leaves insufficient transformable FCC for gradual deformation-induced transformation [7]. The Fe_x_Mn_75−x_Ni_10_Cr_15_ alloy series provides a lower-cost route: Fe_50_Mn_25_Ni_10_Cr_15_ remains single-FCC before deformation, contains abundant twin boundaries, and reaches about 0.98 GPa tensile strength with about 83% ductility at 77 K [11]. Its cryogenic response arises from coupled FCC→HCP TRIP and TWIP rather than a single mechanism [11].

In (Fe_50_Mn_25_Ni_10_Cr_15_)_100−x_Al_x_, increasing Al content shifts the phase constitution from single FCC toward FCC + BCC [16]. This transition reflects the lattice distortion introduced by the relatively large Al atoms and the reduction in VEC, both of which favor BCC formation [16]. At up to 5 at.% Al, the alloy strengthens while retaining an FCC-dominated matrix; beyond 5 at.%, the BCC fraction increases and ductility decreases, although the Al7 alloy still retains measurable tensile ductility at both 298 and 77 K [16]. A recent MA+SPS-processed Al_x_Cr_1−x_CoFeNi HEA study further supports this Al-controlled destabilization route. Replacing Cr with Al progressively increased the BCC fraction from 0 to 32.9 wt.% as x increased from 0.1 to 0.5, raising the UTS to 1163.14 MPa in Al_0.5_Cr_0.5_CoFeNi, while reducing the elongation to 26.98%. These results illustrate the strength–ductility trade-off associated with Al-promoted FCC→BCC transformation and compositional heterogeneity [43].

**Figure 4:**
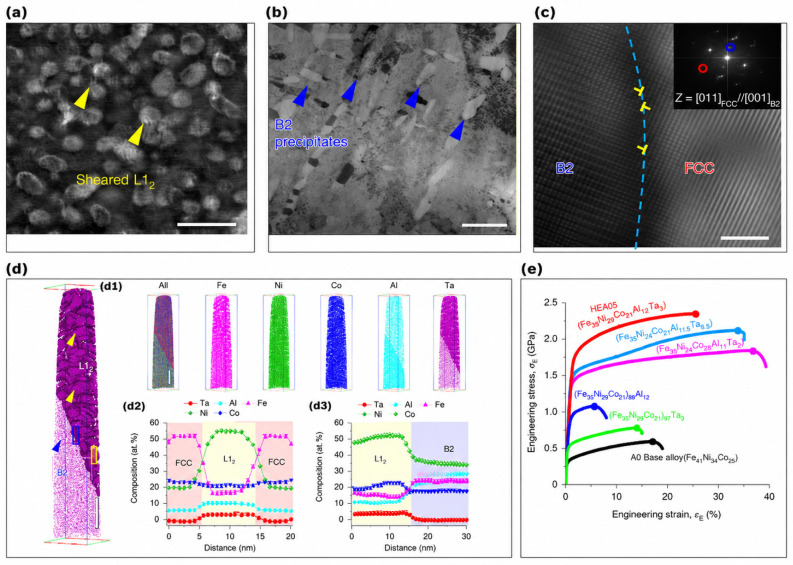
Al/Ta-enabled ordered precipitation and mechanical response in the FeNiCoAlTa multi-principal-element alloy. (**a**) STEM image showing sheared coherent L1_2_ nanoprecipitates in the FCC matrix. (**b**) STEM image showing B2 precipitates distributed within the FCC matrix. (**c**) Atomic-resolution image of the FCC/B2 interface, together with the corresponding fast Fourier transform, revealing the orientation relationship [011]_FCC_//[001]_B2_. (**d**) Atom-probe tomography (APT) characterization: (**d1**) three-dimensional reconstruction and elemental distributions; (**d2**) composition profile across the FCC/L1_2_ region; and (**d3**) composition profile across the L1_2_/B2 region, showing preferential partitioning of Ta, Al, and Ni to the L1_2_ precipitates and enrichment of Al and Ni in the B2 phase. (**e**) Engineering stress–strain curves of the Al-free base alloy, Al-only, Ta-only, and Al–Ta-containing alloys. Al addition markedly increases strength but reduces ductility, whereas the coordinated formation of high-volume-fraction L1_2_ nanoprecipitates and deformable B2 particles enables an improved strength–ductility combination. Adapted and recomposed from Ref. [44].

Beyond changing the FCC/BCC phase balance, Al can also regulate ordered precipitation, elemental partitioning, and matrix–precipitate coupling. This complementary effect is illustrated by the FeNiCoAlTa multi-principal-element alloys shown in Figure 4. Relative to the Al-free Fe_41_Ni_34_Co_25_ base alloy, adding 12 at.% Al increased the yield strength from approximately 300 to 800 MPa, but markedly reduced uniform ductility. In contrast, the combined addition of Al and Ta in ${Fe}_{35}{Ni}_{29}{Co}_{21}{Al}_{12}{Ta}_{3}$ generated a high-volume fraction of coherent L1_2_ nanoprecipitates together with deformable, incoherent B2 particles in the FCC matrix. The representative aged alloy reached a yield strength of approximately 1.75 GPa while retaining approximately 25% uniform elongation [44]. Atom-probe results further show that Al and Ni preferentially partition into the B2 phase, whereas Ta, Al, and Ni are enriched in the L1_2_ precipitates [44]. Therefore, the effect of Al should not be interpreted only as FCC destabilization or BCC promotion; it also depends on the chemistry, volume fraction, coherency, and deformability of the resulting ordered phases.

C, Si, Mo, and Fe complicate metastability control by altering matrix chemistry, precipitation, and local heterogeneity. In carbon-doped CoCrFeNiMo MEAs with nominal compositions Co_17.5_Cr_12.5_Fe_55_Ni_10_Mo_4_C_1_ (Mo_4_C_1_) and Co_17.5_Cr_12.5_Fe_55_Ni_10_Mo_3_C_2_ (Mo_3_C_2_), Kwon et al. showed that carbide precipitation changes the FCC matrix composition and lowers the Gibbs free-energy difference for the FCC→BCC transformation (ΔG_FCC→BCC_), making BCC more favorable relative to FCC [18]. The Mo_4_C_1_ and Mo_3_C_2_ alloys contain Cr-rich M_23_C_6_ and (Fe,Mo)-rich M_6_C carbides; these precipitates strengthen the matrix and shift the residual matrix toward lower FCC stability [18]. At 77 K, the Mo_3_C_2_ alloy reaches about 1.01 GPa YS and about 1.98 GPa ultimate tensile strength (UTS) with more than 50% uniform elongation (UE), showing that precipitation strengthening and TRIP can coexist [18]. A direct comparison in the Fe_40_Mn_40_Co_10_Cr_10_ MEA shows that 0.5 at.% C and 1.7 at.% Mo affect SFE, lattice-friction stress, and the FCC→HCP driving force differently: C stabilizes FCC and gives a 77 K strength/ductility combination of about 1022 MPa/~110%, whereas Mo increases metastability and promotes martensite formation at 77 K [45]. Fe enrichment directly destabilizes FCC. In Fe_60_Co_15_Ni_15_Cr_9.5_C_0.5_ and Fe_65_Co_12.5_Ni_12.5_Cr_9.5_C_0.5_, the Fe_65_ MEA contains a less stable FCC phase and a much larger BCC martensite fraction after cold rolling and annealing [46]. Both alloys undergo FCC→BCC TRIP during tension, but the effect is more pronounced in the Fe-rich alloy [46]. Si also reduces cost while tuning stability: Fe_65_Co_6_Ni_6_Cr_15_Si_8_ forms an ultrafine FCC + BCC structure with an average grain size of about 0.34 μm and about 64% FCC phase [17]. At 77 K, its UTS reaches about 1.94 GPa while the UE remains about 31% [17]. Mo-rich alloys further show that composition and processing can act cooperatively; in Co_21_Cr_12.5_Fe_55_Ni_4_Mo_7.5_, short annealing after cold rolling produced μ-phase nanoprecipitates, ultrafine recrystallized grains, and non-recrystallized regions, thereby regulating Lüders-band propagation and DIMT [14]. These examples show that alloying effects in Fe-based MEAs should not be treated as fixed or universal. Instead, each element can produce different primary and secondary effects depending on the base matrix, phase constitution, segregation behavior, precipitation path, and processing history. A concise summary of the matrix- and processing-dependent roles of representative alloying elements is provided in Table 2.

Therefore, alloying design in Fe-based MEAs should focus on compatibility among phase stability, precipitation behavior, and deformation mode, rather than assigning a fixed strengthening or destabilizing role to a single element.

### 2.3. Microstructural Factors Affecting Metastability

Following the conceptual map in Table 1, microstructure should be treated as a kinetic regulator of apparent metastability rather than as an independent thermodynamic descriptor. While composition defines the intrinsic thermodynamic tendency for FCC→HCP or FCC→BCC transformation, grain size, defects, shear bands, precipitates, local segregation, cell boundaries, and recrystallization state determine the nucleation sites, onset strain, transformation rate, and spatial distribution of martensite during deformation. In Fe_50_Mn_30_Co_10_Cr_10_, Li et al. compared coarse- and fine-grained dual-phase alloys from 298 K to 77 K [54]. Low temperature promotes FCC→HCP transformation, but transformation extent and ductility remain governed by the starting grain structure; Wei et al. reached the same conclusion in the Fe-Mn-Co-Cr system [8]. The coarse-grained Fe_50_Mn_30_Co_10_Cr_10_ alloy transformed too rapidly at 77 K and lost ductility, whereas grain refinement delayed transformation and extended it over a broader strain range, producing a superior cryogenic strength–ductility balance [8]. More martensite is therefore not necessarily beneficial; the useful microstructural state is one that distributes transformation over both strain and space. In Co_18.5_Cr_12_Fe_55_Ni_9_Mo_3.5_C_2_, annealing temperature simultaneously changes carbide precipitation, grain size, matrix chemistry, and martensite nucleation sites [13]. Carbide-rich fine-grained samples had lower calculated ΔG_FCC→BCC_ and faster DIMT kinetics, whereas coarse-grained samples nucleated martensite mainly along shear bands and their intersections [13]. These results show that precipitates do not only strengthen the matrix; they can also change the residual matrix composition and redistribute transformation sites. Partial recrystallization provides another route to tune this kinetic pathway. In Fe_60_Co_15_Ni_15_Cr_10_, a bimodal FCC microstructure containing fine recrystallized grains and unrecrystallized regions regulates DIMT and enables multi-stage hardening [28]. Retained deformation substructures modified FCC stability without changing nominal composition and enabled multi-stage hardening [28]. In partially recrystallized Fe_57.5_Co_18_Cr_13_Ni_7.5_Mo_3_C_1_, in situ synchrotron X-ray diffraction resolved this effect more directly [29]. Both recrystallized and non-recrystallized FCC domains underwent DIMT, but their roles changed with strain: early hardening was dominated by DIMT in the recrystallized domain, whereas the dominant contribution shifted to the non-recrystallized domain at larger strain [29]. A related HPT-processed and annealed Fe_65_Ni_15_Mn_8_Co_8_Ti_3_Si_1_ alloy further shows that changing the recrystallized FCC grain size can regulate cryogenic TRIP kinetics by modifying martensite nucleation and transformation progression, without changing the nominal alloy composition [55].

**Figure 5:**
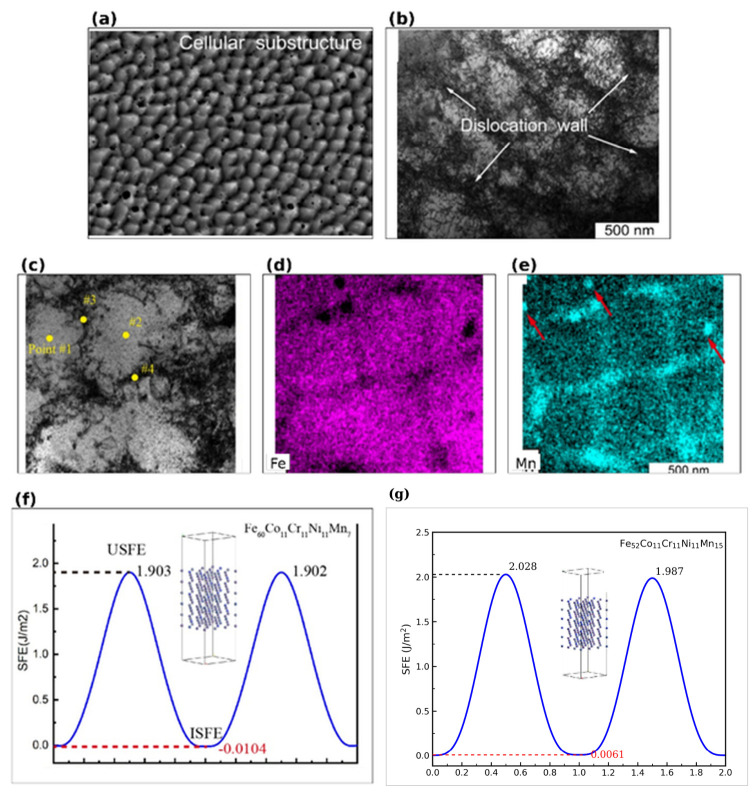
LPBF-induced cellular heterogeneity and composition-dependent stacking-fault energetics in Fe_60_(CoCrNiMn)_40_. (**a**) SEM image showing the cellular substructure generated by rapid solidification during LPBF. (**b**) TEM image revealing that the cellular boundaries consist of dense dislocation walls. (**c**) STEM image of the region selected for local compositional analysis. (**d**,**e**) Corresponding Fe and Mn EDS maps, respectively, showing Fe depletion and Mn enrichment at the cellular boundaries. (**f**,**g**) Generalized stacking-fault-energy curves calculated for Fe_60_Co_11_Cr_11_Ni_11_Mn_7_ and Fe_52_Co_11_Cr_11_Ni_11_Mn_15_, respectively. Increasing Fe content and decreasing Mn content reduce both the energy barrier for partial-dislocation glide and the intrinsic stacking-fault energy, whereas the Mn-enriched cellular boundaries exhibit higher local stacking-fault energy and enhanced FCC stability. These results demonstrate that LPBF-induced dislocation structures and elemental segregation generate a spatially heterogeneous distribution of stacking-fault energy and transformation susceptibility. Adapted and recomposed from Ref. [56] under the Creative Commons Attribution 4.0 International License.

Processing-induced heterogeneity extends this control beyond grain size and recrystallization state. Processing-induced heterogeneity extends metastability control beyond grain size and recrystallization state. In LPBF-fabricated Fe_60_(CoCrNiMn)_40_, rapid solidification and repeated thermal cycling generate cellular and columnar substructures with characteristic dimensions of approximately 500 nm, bounded by dense dislocation walls, as shown in Figure 5 [56]. High-resolution EDS mapping reveals pronounced local chemical heterogeneity across these substructures. The cell-wall regions are depleted in Fe and enriched in Mn, containing approximately 51 at.% Fe and 16 at.% Mn, whereas the cell interiors contain approximately 58 at.% Fe and 11 at.% Mn [56]. First-principles calculations further indicate that this Fe-Mn redistribution modifies the local generalized stacking-fault-energy landscape. The Fe-rich and Mn-lean regions exhibit a lower intrinsic SFE and reduced barriers for partial-dislocation motion, whereas the Mn-enriched cell walls exhibit a higher SFE and locally stabilize the FCC phase [56]. Therefore, LPBF-induced cellular heterogeneity does not simply destabilize the FCC matrix uniformly; instead, it creates a spatial distribution of transformation susceptibility, in which cell interiors, cell walls, dislocation networks, and locally formed Fe-rich BCC regions play different roles in deformation and phase transformation.

For additively manufactured Fe_65_Ni_15_Co_8_Mn_8_Ti_3_Si, Kwon et al. [57] linked cellular/dual-region heterogeneity to multi-stage hardening at room and liquid-nitrogen temperatures, using a semi-empirical model to relate DIMT to hardening. Surface severe plasticity supplies a localized version of the same principle: Gu et al. showed that modifying only the near-surface region of Fe-based MEAs can reduce apparent SFE and accelerate martensitic transformation [58]. The resulting gradient layer introduces refined grains, higher dislocation density, preferential martensite-nucleation sites, and stress partitioning across the surface-modified region [58]. Dislocation-engineered Fe_50_Mn_30_Co_10_Cr_10_ follows a related logic. Chen et al. used sequential cold–warm rolling to introduce incident dislocation boundaries and refined dislocation-cell structures [59]. The cold–warm-rolled alloy achieved a YS of about 985 MPa and maintained a hardening rate of about 3.5 GPa at 77 K [59]. Cryogenic pre-straining, surface modification, SLM, laser-directed energy deposition (laser-DED), and ultrafast induction heating tune local metastability through retained defects, cell boundaries, or solute segregation without changing nominal composition [30,60,61,62,63]. Laminate processing offers another route: DED-fabricated Fe_75_(CoCrNi)_25_/Fe_60_(CoCrNi)_40_ uses a mixed FCC/BCC transition layer and interface constraint to coordinate strain transfer and improve strength–ductility balance [64]. Overall, microstructure changes the apparent metastability of Fe-based MEAs: composition defines the thermodynamic tendency, whereas grain boundaries, shear bands, precipitates, cell walls, dislocation boundaries, and gradient interfaces determine transformation sites and their contribution to uniform plasticity.

## 3. Representative Composition Systems of Fe-Based MEAs

Representative Fe-based MEAs are not simply different composition lists, but can be classified according to their dominant design objectives and phase-stability routes. To avoid treating each alloy family as an isolated case, Table 3 summarizes the major composition families in terms of design objective, phase-stability control, processing route, representative property range, and remaining limitations. This classification provides a comparative framework for the following discussion and clarifies why similar tensile properties may arise from different combinations of metastability, precipitation, heterogeneity, and deformation mode.

### 3.1. Fe-Co-Ni-Cr-Based Systems

Fe-Co-Ni-Cr is a compositional framework rather than a single alloy family with fixed properties. It retains an FCC matrix over a broad composition range, while remaining sensitive to the Fe/(Co, Ni, Cr) balance. Compared with Mn-controlled low-SFE systems, Fe-Co-Ni-Cr-based MEAs mainly rely on the Fe/(Co, Ni, Cr) balance to regulate FCC stability and activate FCC→BCC deformation-induced martensitic transformation. Therefore, this family is particularly useful for understanding BCC-DIMT-assisted cryogenic hardening and phase-stress partitioning, but it is also sensitive to excessive FCC destabilization and localized martensite formation. In composition-gradient Fe_x_(CoCrNi)_100−x_ alloys, increasing Fe changes the constitution from FCC to FCC + BCC and then to BCC [66]. To clarify the Fe-content-dependent transition from FCC stability to BCC-DIMT-assisted deformation, the representative phase constitution and deformation behavior of Fe-Co-Ni-Cr-based MEAs are summarized in Table 4.

This comparison shows that increasing Fe content does not simply improve transformation hardening. Instead, Fe enrichment shifts Fe-Co-Ni-Cr-based MEAs from relatively stable FCC deformation toward metastable FCC-assisted FCC→BCC DIMT and, eventually, BCC-containing or over-destabilized structures. FCC→BCC DIMT is advantageous when the parent FCC phase remains sufficiently stable during early deformation but transforms progressively at larger strain. Under this condition, newly formed BCC martensite provides additional phase boundaries, promotes phase-stress partitioning, and sustains work hardening. In contrast, when Fe enrichment excessively lowers FCC stability, martensite can form too early, too rapidly, or too locally. This premature transformation exhausts the transformable FCC matrix, increases local hardening mismatch, and can lead to quicker necking, especially under uniaxial tension.

Fe_65_Ni_15_Cr_10_Co_10_ also develops a heterogeneous FCC/BCC structure after cold rolling and low-temperature annealing, offering a route to cryogenic strength–ductility synergy [69]. Recent studies on Fe_60_Co_15_Ni_15_Cr_10_ further show that thermally activated deformation, hot-rolling-induced heterogeneity, and strain-rate-sensitivity/DIMT coupling regulate cryogenic hardening [70,71]. In the related Fe_x_(CoCrMnNi)_100−x_ ferrous MEA series, Fe_60_ remains a single-FCC TWIP-dominant alloy, Fe_65_ combines TWIP and TRIP, and Fe_70_ contains BCC and shows stronger TRIP, higher strength, and lower ductility [67]. In Fe_65_Co_10_Ni_10_Cr_15_, lower FCC stability produces stronger TRIP and higher cryogenic strength [68]. In situ neutron diffraction shows that the transformed BCC phase is mechanically active: its stress contribution increases during low-temperature loading, while that of FCC decreases [10]. Constitutive models describe this response by coupling transformation initiation, BCC-fraction evolution, FCC hardening, and phase partitioning [72]. Kwon et al. [72] further used a critical initiation-stress criterion, martensite-fraction evolution, dislocation-based FCC hardening, and finite-element method (FEM) validation to describe cryogenic FCC→BCC DIMT in Fe_60_Co_15_Ni_15_Cr_10_. In the less stable Fe_64_(CoNi)_26_Cr_10_ alloy, stronger DIMT improves hardening but can trigger early necking and reduce UE under uniaxial tension, while remaining beneficial under biaxial stretching [40]. Therefore, Fe content defines the overall FCC stability window, but the mechanical benefit of FCC→BCC DIMT depends on whether transformation kinetics are delayed, progressive, and spatially distributed. Moderate Fe-induced metastability promotes cryogenic hardening and phase-stress partitioning, whereas excessive Fe-induced destabilization can trigger rapid martensite formation, strain localization, and premature necking [5,28,30,40].

### 3.2. Fe-Mn-Cr-Ni/Co-Based Systems

Fe-Mn-Cr-Ni/Co alloys follow a different route. Mn regulates FCC stability and SFE, thereby selecting slip, TWIP, FCC→HCP TRIP, or their combination [7,8,11,47]. Unlike Fe-Co-Ni-Cr-based systems, where FCC→BCC DIMT is often the central transformation route, Fe-Mn-Cr-Ni/Co-based alloys are governed mainly by SFE-mediated deformation competition. Their key design problem is not simply to increase the martensite fraction, but to maintain a low-SFE FCC matrix in which dislocation slip, TWIP, and FCC→HCP TRIP can operate progressively over a broad strain range. Co-free Fe-24Mn-22Ni-12Cr shows that cryogenic caliber rolling increases YS through higher dislocation density and twinning while retaining ductility [73]. Fe_40_Mn_20_Cr_20_Ni_20_ shows that partial recrystallization raises cryogenic strength, while twins and dislocation accumulation sustain ductility at 77 K [74]. A separate grain-refinement comparison between Cr_20_Mn_24_Fe_30_Co_20_Ni_6_ and Cr_20_Mn_15_Fe_34_Co_20_Ni_11_ clarifies the mechanism: short annealing after cold rolling preserved TWIP and improved the strength–ductility balance in the Cr_20_Mn_15_Fe_34_Co_20_Ni_11_ TWIP alloy, whereas nano-sized Cr-rich sigma precipitates strengthened the Cr_20_Mn_24_Fe_30_Co_20_Ni_6_ TRIP alloy but promoted micro-void dispersion and reduced ductility [75]. Fe_55_Mn_25_Co_10_Cr_10_ shows this tunability: deformation-induced ε-martensite and FCC twins accommodate strain at room temperature, whereas bi-directional ε-martensite bands and HCP twins dominate at 77 K, giving a multi-step TRIP/TWIP(FCC, HCP) response [47]. A recent low-Co design further demonstrates that phase stability in Fe-Mn-Co-Cr alloys can be regulated through coupled composition and processing control. In LMD-fabricated Fe_60_Mn_27_Co_3_Cr_10_, the reduction in Co content relative to Fe_50_Mn_30_Co_10_Cr_10_ is accompanied by increased Fe and slightly reduced Mn contents, producing a uniform FCC+HCP dual-phase structure [76]. The HCP fraction ranges from approximately 51.2% to 59.9% on different deposition planes, and the FCC and HCP phases maintain the Shoji–Nishiyama orientation relationship. Comparisons with Fe_50_Mn_30_Co_10_Cr_10_ processed through casting, powder metallurgy, SLM, and LMD show that the low-Co composition and rapid-solidification route jointly refine the prior-FCC grains, increase the HCP fraction, and reduce the FCC/HCP inter-lath spacing to approximately 0.8–0.9 μm [76]. These changes enhance grain- and phase-boundary strengthening while maintaining tensile elongations of approximately 25–32%. Because Fe, Mn, and Co contents change simultaneously, the resulting phase stability should be interpreted as a coupled compositional effect rather than an isolated Co effect. The accelerated martensitic transformation in V_10_Cr_10_Co_30_Fe_50_ and the grain-size-dependent response of Fe_50_Mn_30_Co_10_Cr_10_ together show that reduced FCC stability improves cryogenic strength only when transformation remains spatially distributed [8,54,77].

**Figure 6:**
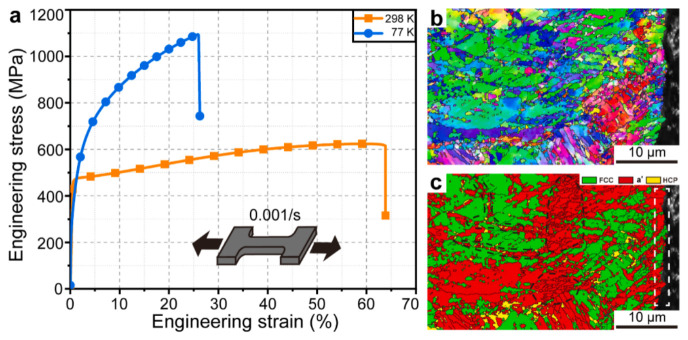
Temperature-dependent tensile response and cryogenic deformation-induced phase transformation in the LPBF-fabricated Fe_60_(CoCrNiMn)_40_ alloy. (**a**) Engineering stress–strain curves obtained at 298 and 77 K at a strain rate of 0.001 s^−1^. (**b**,**c**) EBSD inverse-pole-figure and phase maps, respectively, acquired adjacent to the tensile fracture surface at 77 K. Cryogenic deformation substantially increases strength and induces extensive dynamic grain refinement and transformation of the FCC matrix predominantly into BCC/BCT martensite, accompanied by a smaller fraction of HCP phase. Adapted from Ref. [56] under the Creative Commons Attribution 4.0 International License.

Fe_50_Mn_25_Ni_10_Cr_15_ remains single-FCC before deformation, contains abundant twin boundaries, and reaches about 0.98 GPa tensile strength and about 83% ductility at 77 K through coupled TWIP and TRIP [11]. Fe_49.5_Mn_25_Cr_15_Ni_10_C_0.5_ shows a related cryogenic advantage: ductility increases from 49% at 298 K to 71% at 77 K, and the UTS-ductility product reaches about 75 GPa·%, exceeding those of its subsystem steels [12]. Its deformation mode is distinct: TWIP dominates in the MEA, whereas the subsystem alloys favor HCP- or BCC-type martensitic transformation [12]. The temperature-dependent tensile response of an LPBF-fabricated Fe_60_(CoCrNiMn)_40_ alloy provides further evidence that cryogenic strengthening is closely coupled with deformation-induced phase transformation. As shown in Figure 6, the alloy exhibits an ultimate tensile strength of approximately 623 MPa and an elongation of about 63% at 298 K. When the temperature decreases to 77 K, the yield strength and ultimate tensile strength increase to approximately 665 MPa and 1.1 GPa, respectively, while about 25% tensile ductility is retained [56]. EBSD characterization near the cryogenic fracture surface reveals extensive dynamic grain refinement and transformation of the FCC matrix predominantly into BCC/BCT martensite, accompanied by a smaller amount of HCP phase. These results indicate that lowering temperature strongly increases transformation activity and strain hardening, but the progressive consumption of ductile FCC and the accumulation of BCC/BCT near the fracture region eventually reduce the remaining plastic accommodation capacity [56]. These results show that Mn places FCC in a low-SFE yet deformable regime where twinning and transformation cooperate [11,12,78]. Mo alloying offers an additional route to control secondary phases in the same Fe_50_Mn_27_Ni_10_Cr_13_ base: in (Fe_50_Mn_27_Ni_10_Cr_13_)_100−x_Mo_x_, cold rolling and annealing of the Mo_2_ alloy introduced secondary phases, ordered BCC nanoparticles, twins, and dislocation cells, increasing tensile strength from about 250 MPa to 665 MPa while retaining about 25% ductility [79].

Mn-induced metastability also supports heterostructure design. Fe_55_Mn_20_Cr_15_Ni_10_ gains strength after heterogeneous lamella construction and cryo-pre-straining by introducing HCP martensite, dislocations, twins, and stacking faults into the metastable FCC matrix [78]. In Cr_15_Fe_55_Mn_20_Ni_10_, cryorolling followed by short-time annealing produces a phase-reversion heterostructure that increases YS while retaining about 44% elongation [80]. Thus, grain size, pre-strain, and heterostructure design further regulate the low-SFE austenitic base [8,54,78,80].

Ni, Co, C, grain size, and pre-strain redistribute TWIP/TRIP contributions within this Mn-controlled framework. Thus, the same compositional family can show high cryogenic ductility in Fe_50_Mn_25_Ni_10_Cr_15_ [11], TWIP-dominant deformation in Fe_49.5_Mn_25_Cr_15_Ni_10_C_0.5_ [12], and reduced ductility when the initial HCP fraction is excessive [7].

### 3.3. Fe-Co-Cr-Ni-Mo-Based Systems

Mo-bearing Fe-Co-Cr-Ni-Mo alloys enable a substitutional-solute strengthening pathway. Unlike Fe-Co-Ni-Cr and Fe-Mn-Cr-Ni/Co systems, Mo-bearing alloys introduce precipitation and elemental partitioning as additional variables for metastability control. Mo not only increases lattice resistance, but can also promote μ-phase or carbide precipitation, modify the residual FCC matrix composition, and thereby change FCC→BCC transformation kinetics during deformation. In Co_17.5_Cr_12.5_Fe_55_Ni_10_Mo_5_, Mo enhances strength via solid-solution and precipitate hardening, achieving a yield strength of 1075 MPa at 4.2 K by grain-boundary and forest hardening [65]. High-throughput DED extends this concept across the Fe-Co-Cr-Ni-Mo composition space: in situ alloying produced 27 Fe-Co-Cr-Ni-Mo compositions and identified Fe_53.9_Co_16.8_Cr_11.7_Ni_11.5_Mo_6.0_ as an optimized composition [81]. Further strengthening can be achieved by HPT, annealing, UNSM, and hierarchical microstructure design, although these effects are superimposed on an already strengthened Mo-bearing matrix [14,15,82]. Fe_60_Co_20_Ni_15_Mo_5_ MEA, C/Mo/V co-doped (Fe_57.5_Co_20_Cr_12.5_Ni_5_Mo_3_V_2_)_99.8_C_0.2_ HEA, and Fe_60_Co_25_Ni_10_Mo_5_ maraging MEA demonstrate that Mo supports high-density nanoprecipitation, μ-phase/MC-type precipitates, and maraging-related transformation hardening [83,84,85]. In Co-Cr-Fe-Ni-Mo-C alloys, carbide precipitation, matrix partitioning, lattice-distortion/back-stress effects, and post-laser powder bed fusion (LPBF) cell restructuring tune DIMT kinetics and hardening [86,87,88]. A recent C/Mo microalloying study on hot-rolled [Co_40_Cr_25_(FeNi)_35−y_Mo_y_]_100−x_C_x_ HEAs further confirms this matrix-dependent strengthening route: C-free Mo3-Mo5 alloys exhibited FCC+HCP dual phases, whereas 0.5 at.% C addition promoted an FCC-dominated structure, refined the Mo_4_C_0.5_ microstructure, and enabled a favorable combination of 757 MPa YS, 1186 MPa UTS, and 69% elongation through coupled fine-grain strengthening, TWIP effect, and solid-solution strengthening [89]. Because Mo-bearing Fe-based MEAs can be strengthened through several coupled but distinct mechanisms, the Mo-specific strengthening and stability-control routes are separated in Table 5.

Table 5 shows that Mo does not act through a single strengthening mechanism. When Mo remains mainly in solid solution or forms fine, well-distributed precipitates, it can increase lattice resistance and precipitation strengthening while retaining useful plasticity. When Mo partitions strongly into μ-phase or carbide precipitates, the surrounding FCC matrix may become chemically modified and more susceptible to FCC→BCC DIMT. This partitioning-induced destabilization is beneficial only when martensitic transformation remains gradual and spatially distributed. In contrast, ductility loss occurs when coarse intergranular μ-phase, excessive carbide precipitation, or processing-induced local instability promotes strain localization. 

Mo also regulates FCC stability and deformation-induced transformation. In (Fe_62_Co_8_Ni_15_Cr_15_)_100−x_Mo_x_ medium-entropy alloys, Mo addition promotes Mo-rich nanoscale precipitation while modifying the phase transformation tendency of the FCC matrix. Tensile tests at cryogenic temperature reveal that all alloys remain FCC at 298 K but undergo FCC→BCC transformation at 77 K. Increasing Mo content progressively decreases the BCC fraction after fracture, indicating that Mo stabilizes the FCC phase and suppresses excessive transformation. This balance between precipitation strengthening and controlled TRIP activation is essential for achieving high strength–ductility synergy [91]. 

For Mo-bearing alloys, design depends on coupling solute strengthening, precipitation, and matrix destabilization. Mo increases FCC lattice resistance, forms μ or carbide precipitates, and modifies the surrounding matrix chemistry, explaining why Mo-containing systems show either high strength with limited ductility or balanced properties, depending on annealing path, defect state, and precipitate distribution [13,14,15,65,90]. Laser-treated Co_17.5_Cr_12.5_Fe_55_Ni_10_Mo_5_ illustrates the processing trade-off: single laser scanning increases YS from about 391 to 844 MPa but reduces UE from about 40% to about 8%, whereas the optimized laser-scanning condition retains a more balanced response [90]. UNSM-treated Fe_55_Co_19_Ni_8.5_Cr_12.5_Mo_5_ and Fe_55_Co_20_Ni_5_Cr_12.5_Mo_7.5_ show that lowering FCC stability from Fe_55_Co_19_Ni_8.5_Cr_12.5_Mo_5_ to Fe_55_Co_20_Ni_5_Cr_12.5_Mo_7.5_ shifts deformation from twinning toward transformation and strengthens HDI hardening [82]. Surface-treated FeCoNiCrMo MEAs show that UNSM-induced gradients and Co/Ni-controlled FCC stability enhance HDI hardening without providing direct μ- or carbide-precipitation evidence [82]. 

Therefore, the design of Mo-bearing Fe-based MEAs should distinguish four coupled but different effects: solid-solution strengthening by dissolved Mo, precipitation strengthening by μ-phase or carbides, partitioning-induced FCC destabilization, and processing-controlled heterogeneity. High strength is usually obtained when these effects operate cooperatively, whereas ductility decreases when coarse precipitates, carbide-rich regions, or overly rapid DIMT create local deformation incompatibility.

### 3.4. Modified Systems Containing Al, Ti, Si, and Interstitial C/B/N Additions

Al, Ti, Si, and interstitial C/B/N additions should be regarded as modification routes rather than a single composition family. Compared with the base Fe-Co-Ni-Cr, Fe-Mn-Cr-Ni/Co, and Fe-Co-Cr-Ni-Mo systems, these additions expand the design space by adjusting phase constitution, precipitation behavior, local chemical order, and interface stability. Their effects are strongly matrix- and processing-dependent: Al and Si mainly promote FCC + BCC/B2 phase control and low-cost transformation-assisted strengthening, Ti introduces ordered or carbide-related precipitation and stability changes, whereas C, B, and N regulate interstitial strengthening, local chemical order, carbide/nitride formation, and TWIP/TRIP activity.

#### 3.4.1. Al

Al primarily modifies phase constitution and elemental partitioning in ferrous MEAs. The beneficial effect of Al is therefore conditional. Al is most effective when it promotes controlled FCC/BCC or FCC/B2 heterogeneity while preserving an FCC-rich or FCC-connected matrix. In this case, BCC/B2 domains or ordered precipitates can contribute to load bearing, precipitation strengthening, and sustained strain hardening. In contrast, excessive Al addition may shift the alloy toward a BCC/B2-dominated or brittle intermetallic-containing structure, which weakens deformation compatibility and causes a ductility penalty. The Al-containing Fe_61.5_Cr_17.5_Ni_13_Al_8_ alloy exhibits a heterogeneous FCC + BCC + minor B2 structure, with a YS of 627 MPa, UTS of 890 MPa, and ductility of 27.3% at room temperature [92]. Ti co-alloying intensifies this effect: in (Fe_65_Ni_15_Cr_10_Co_10_)_92_Ti_5_Al_3_, Ti and Al form heterogeneous nanoprecipitates in a partially recrystallized matrix and reduce FCC stability; precipitation strengthening, HDI strengthening, and continuous FCC→BCC transformation produce 960 MPa YS, 1519 MPa UTS, and about 22% UE [93]. Fe_50_Mn_20_Al_15_Ni_10_Co_5_ demonstrates the role of Al in forming B2/BCC/FCC multiphase load-bearing structures [94]. FeMnAl(Cu) low-density MEAs further show that Al-containing Fe-Mn-Al matrices can be hardened over a wide range through β-Mn precipitation, thereby improving hardness and wear resistance [95]. Fe_47_Mn_25_Al_13_Cr_7_Ni_5_C_3_ highlights a lightweight Al-containing MEA design in which partial recrystallization, ultrafine grains, and M_23_C_6_/B2 precipitates provide different strength-elongation combinations [96].Fe_48−x_Mn_32_Co_10_Cr_10_Al_x_ shows that Al raises SFE and shifts the constitution and mechanism from FCC+HCP martensitic transformation to FCC twinning and then FCC + BCC dislocation gliding [97]. In the separate Fe-Cr-Ni-Al MEAs, CALPHAD-guided Al variation changes the constitution from FCC to FCC + BCC/B2 and finally BCC/B2; therefore, the as-cast alloy containing 11 at.% Al is better interpreted as a cost-effective BCC/B2 phase-control and precipitation-strengthening example, rather than as a ductile TRIP-type MEA [98].

**Figure 7:**
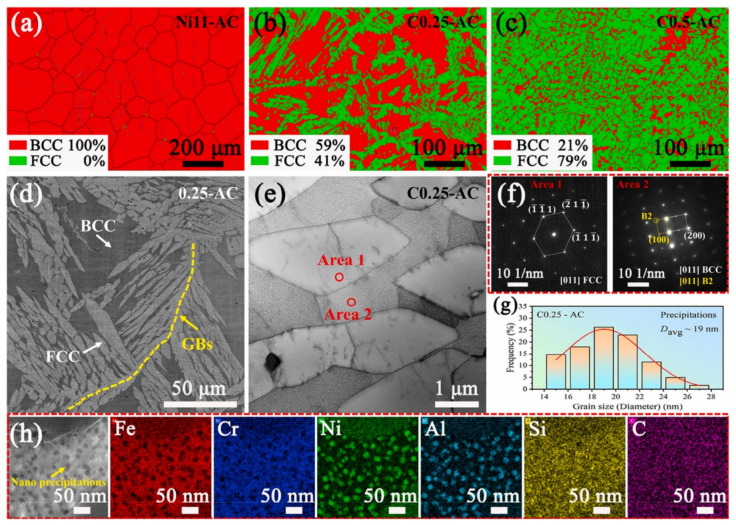
C-assisted phase balancing and B2 precipitation in the Al-containing Co-free (Fe_64_Ni_11_Cr_15_Si_7_Al_3_)_100−x_C_x_ medium-entropy alloys. (**a**–**c**) EBSD phase maps of the as-cast Ni11, C0.25, and C0.5 alloys, respectively, showing the evolution from a single BCC structure to FCC + BCC dual-phase structures with increasing C content. (**d**) BSE image of the C0.25 alloy showing the distribution of FCC and BCC regions. (**e**–**h**) TEM images, selected-area electron diffraction patterns, precipitate-size statistics, and HAADF elemental maps of the C0.25 alloy, revealing nanoscale Ni- and Al-rich B2 precipitates within the BCC matrix. The results demonstrate that interstitial C stabilizes FCC and regulates the FCC/BCC phase balance while retaining Al-associated B2 precipitation. Reproduced from Ref. [100].

Fe_62_Co_5_Ni_10_Cr_13_Si_7_Al_3_ provides a low-cost Al-containing route, reaching about 1640 MPa YS, 2109 MPa UTS, and 29.1% UE at 77 K through high-density dislocations, ultrafine grains, nanoprecipitates, Lüders deformation, HDI hardening, and DIMT [99]. A complementary Co-free strategy is to balance the strong BCC/B2-forming tendency of Al through interstitial C alloying. In (Fe_64_Ni_11_Cr_15_Si_7_Al_3_)_100−x_C_x_, the C-free Ni11 alloy exhibits a single BCC structure, whereas additions of 0.25 and 0.5 at.% C produce FCC + BCC dual-phase structures, as shown in Figure 7. The FCC fraction increases to approximately 41% in the C0.25 alloy and 79% in the C0.5 alloy. TEM and elemental mapping further reveal nanoscale B2 precipitates enriched in Ni and Al within the BCC matrix. These results demonstrate that the effect of Al cannot be considered independently: Al promotes BCC/B2 formation and ordered Ni-Al precipitation, whereas interstitial C stabilizes FCC and regulates the connectivity and volume fraction of the deformable FCC phase [100]. 

Fe_62_Co_5_Ni_10_Cr_13_Si_7_Al_2_Mo_1_ reaches about 1.1 GPa YS and 44% ductility at room temperature with an FCC/BCC heterostructure, annealing twins, and nanoprecipitates; the precipitated phase is inferred to be B2-NiAl [101]. These examples confirm that Al acts mainly through phase partitioning, BCC/B2 formation, and precipitation-assisted strengthening. Al can expand the lattice, reduce VEC, promote BCC/B2 formation, and interact with Ni or Ti to form ordered precipitates. However, the design target should be controlled FCC/BCC or FCC/B2 heterogeneity rather than a fully BCC/B2-dominated structure. Therefore, the useful Al pathway is a balanced architecture that strengthens the alloy while maintaining FCC connectivity, strain compatibility, and continued hardening during deformation [92,93,99,101].

#### 3.4.2. Ti

**Figure 8:**
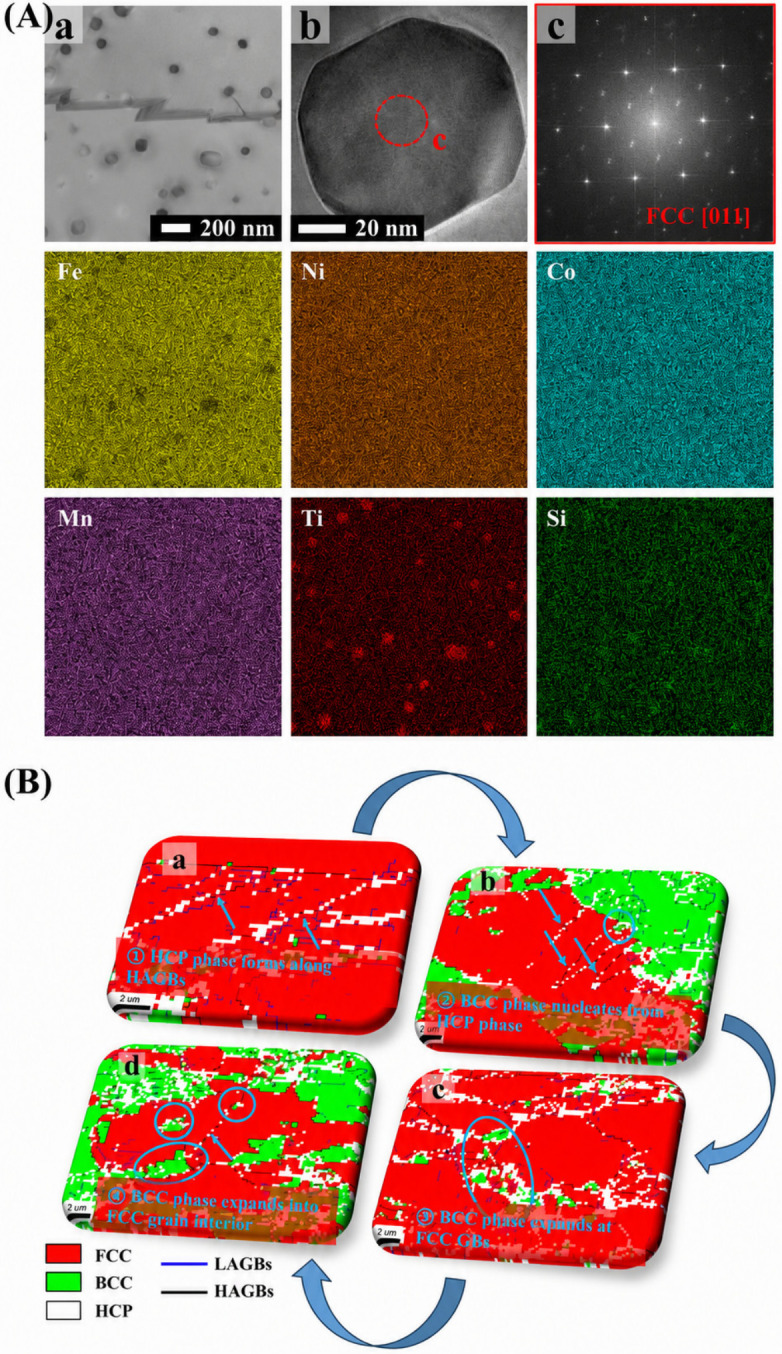
Ti-Si-containing nanoprecipitation and sequential martensitic transformation in the cryo-rolled and annealed Fe_62_Ni_16_Co_9_Mn_9_Ti_3_Si_1_ high-entropy alloy. (**A**) TEM characterization of spherical (Fe,Ni)_2_SiTi nanoprecipitates in the CR800 condition: (**a**) TEM image showing the distribution of spherical nanoprecipitates; (**b**) high-resolution TEM image of a representative nanoprecipitate; and (**c**) corresponding diffraction pattern, together with Fe, Ni, Co, Mn, Ti, and Si elemental maps. The precipitates are preferentially enriched in Ti and Si. (**B**) Local EBSD phase maps after tensile deformation, showing HCP formation along high-angle grain boundaries, subsequent BCC nucleation from HCP regions, and BCC growth along FCC grain boundaries and into grain interiors. The results reveal a progressive FCC→HCP→BCC transformation pathway coupled with precipitation and heterogeneous-structure strengthening. Adapted and recomposed from Ref. [107].

Ti controls precipitation, stability, and deformation mode, but its effect depends strongly on alloy system and processing. To avoid conflating different Ti effects, the following discussion separates Ti-related design routes into four categories: martensitic matrix design, precipitation strengthening, TiC-related matrix stabilization, and carbide strengthening with C. In the martensitic matrix design route, Ti works together with Al and Mo to regulate FCC stability, BCC martensite formation, dislocation storage, and μ-phase precipitation. Fe_50_Co_25_Ni_10_Al_5_Ti_5_Mo metastable alloy has a calculated ΔG_FCC→BCC_ of about -2800 J mol^−1^ at room temperature; the hot-rolled state contains retained FCC, thermally induced BCC martensite, high dislocation density, and Mo-rich μ precipitates [25,35,102]. It reaches about 1.5 GPa UTS at 298 K and about 2.3 GPa UTS at 77 K, with uniform elongations of about 15% and 11%, respectively [25]. In the precipitation-strengthening route, Ti promotes nanoscale or coherent precipitates and strengthens precipitate-matrix interfaces. Fe_61.75_Ni_14.25_Co_7.6_Mn_7.6_Ti_2.85_Si_0.95_Cu_4.5_Al_0.5_ demonstrates a Ti-bearing precipitation/spinodal-decomposition route involving Fe2SiTi and Ni3Ti nanoprecipitates [103], whereas the Fe_68_Ni_10_Mn_10_Co_10_Ti_1.5_Si_0.5_ series illustrates maraging/reversion design with reversion-induced austenite and Ti-containing nanoprecipitates such as (NiMn)_3−x_Ti_x_, η-Ni_3_Ti, and Ni_2_SiTi [32,104,105]. Ti microalloying in BCC-type Ti_x_Ni_0.6_CoFe_1.4_ MEAs shows that Ti can promote FCC/R phase precipitation, grain refinement, and coherent precipitate–matrix boundary strengthening [106]. The additively manufactured Fe_65_Ni_15_Co_8_Mn_8_Ti_3_Si system is cited as a DIMT and multi-stage strain-hardening modeling case rather than as a conventional cast/rolled precipitation-strengthened alloy [57]. A complementary Ti-Si precipitation route is demonstrated by the cryo-rolled and annealed Fe_62_Ni_16_Co_9_Mn_9_Ti_3_Si_1_ alloy. As shown in Figure 8, annealing at 800 °C produces high-density spherical (Fe,Ni)2SiTi nanoprecipitates enriched in Ti and Si. These particles have a volume fraction of approximately 2.94% and an average radius of about 15.24 nm [107]. Their formation modifies the residual matrix chemistry and contributes to precipitation strengthening while retaining a metastable FCC matrix. During subsequent tensile deformation, HCP martensite initially forms along high-angle grain boundaries, after which BCC martensite nucleates from the HCP regions and expands along grain boundaries and into FCC grain interiors. The gradual consumption of HCP during BCC growth indicates a sequential FCC→HCP→BCC transformation pathway [107].

This case demonstrates that Ti does not act solely through precipitate hardening. Through its coupling with Si, annealing state, defect density, and heterogeneous grain structure, Ti-containing precipitation also modifies matrix chemistry and transformation kinetics. In the CR800 condition, the coexistence of ductile nanoprecipitates, bimodal grains, and progressive FCC→HCP→BCC transformation sustains strain hardening and contributes to a uniform elongation of approximately 53% [107].

Ti can also suppress transformation by stabilizing austenite. In DED-processed Fe_60_Co_15_Ni_15_Cr_10_ reinforced with TiC nanoparticles, partial decomposition releases Ti and C into the matrix, stabilizes γ-austenite, suppresses DIMT, and shifts deformation toward slip [27]. This case should be treated as a warning for TiC-reinforced DED materials: TiC particles may provide particle-related strengthening or microstructural refinement, but partial TiC decomposition can overstabilize the FCC matrix and suppress beneficial transformation-assisted hardening. In the carbide-strengthening-with-C route, Ti should be distinguished from TiC-particle reinforcement because Ti is introduced together with C and thermomechanical processing to form a carbide-containing, defect-rich FCC structure. In C-Ti microalloyed Fe-Mn-Cr-Ni-based MEAs, Ti should be distinguished from TiC-particle reinforcement because Ti is introduced together with C and thermomechanical processing to form carbide-containing, defect-rich FCC structures. One representative cryogenic-rolling/low-temperature-annealing route produces an unrecrystallized FCC matrix containing dislocations, nanoscale carbides, nanotwins, and microbands, achieving YS/UTS values of 1256/1329 MPa at 298 K and 1682/1802 MPa at 77 K while retaining ductility above 10% [26]. A related Co-free (Fe_40_Mn_40_Cr_10_Ni_10_)_96.6_C_3_Ti_0.4_ MEA further demonstrates that combined Ni/Cr optimization, C addition, Ti microalloying, cold rolling, and annealing can deliver YS/UTS values of 1013/1171 MPa at 298 K and 1339/1526 MPa at 77 K with elongation not less than 15% [108]. Ti is therefore a microstructural regulator whose effect depends on co-alloying, precipitate type, and retained strain-hardening capacity. Its role should be interpreted according to the specific design route: coherent or ordered precipitation, B2/μ-assisted martensitic matrix design, TiC-related matrix stabilization, or Ti-C carbide-strengthened FCC structures. Therefore, Ti is best viewed as a phase- and interface-control element rather than a simple strengthener [25,26,27,102,109].

#### 3.4.3. Si

Si mainly lowers FCC stability and promotes transformation-assisted deformation, but its engineering benefit depends on whether Lüders-band propagation and DIMT can be spatially controlled. Fe_65_Co_6_Ni_6_Cr_15_Si_8_, designed by partly replacing Co and Ni in Fe_65_Co_10_Ni_10_Cr_15_ with Si, forms an ultrafine FCC + BCC microstructure with an average grain size of about 0.34 μm and about 64% FCC [17]. At 298 K, it reaches 902 MPa YS, 1214 MPa UTS, and 26% UE; at 77 K, these values increase to 1140 MPa, 1938 MPa, and 31%, respectively [17]. Lüders deformation occurs at both temperatures. However, Lüders deformation should not be interpreted as universally beneficial. Although it can coincide with transformation-assisted hardening in tensile specimens, Lüders-band propagation may be undesirable for forming operations and structural reliability if it is not properly controlled. The localized propagation of a deformation band can introduce strain heterogeneity, surface marking, and nonuniform plastic flow, thereby reducing formability and increasing the risk of local damage initiation.

**Figure 9:**
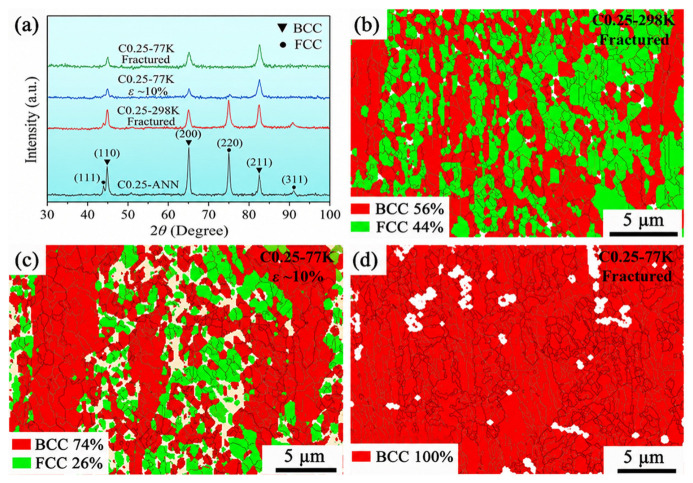
Temperature- and strain-dependent FCC→BCC transformation in the C0.25-ANN (Fe_64_Ni_11_Cr_15_Si_7_Al_3_)_100−x_C_x_ medium-entropy alloy. (**a**) XRD patterns of the annealed alloy and specimens deformed at 298 and 77 K. (**b**) EBSD phase map adjacent to the fracture region after tensile deformation at 298 K, showing an essentially unchanged FCC/BCC phase balance. (**c**) EBSD phase map at approximately 10% strain at 77 K, showing an increase in the BCC fraction to approximately 74%. (**d**) EBSD phase map after fracture at 77 K, showing nearly complete transformation of FCC into BCC. The results reveal progressive cryogenic martensitic transformation during and after the extended yield-platform stage. Reproduced from Ref. [100].

A complementary Co-free Si-bearing alloy illustrates that the cryogenic response of Si-containing Fe-MEAs is strongly dependent on interstitial alloying and the initial FCC/BCC phase balance. In the C0.25-ANN alloy of the (Fe_64_Ni_11_Cr_15_Si_7_Al_3_)_100−x_C_x_ system, the annealed microstructure initially contains approximately 45% FCC and 55% BCC. As shown in Figure 9, tensile deformation at 298 K produces almost no measurable phase transformation, with the fractured specimen retaining approximately 44% FCC and 56% BCC. At 77 K, however, the BCC fraction increases to approximately 74% at a strain of about 10%, near the end of the extended yield plateau, and the fractured region becomes almost fully BCC [100].

The cryogenic stress–strain response is characterized by an extended yield platform of approximately 8.4% and a subsequent multi-stage strain-hardening response, with the strain-hardening rate increasing to approximately 6115 MPa after the yield-platform stage [100]. These results demonstrate that the transformation behavior of a Si-bearing matrix cannot be attributed to Si alone. Instead, it reflects the coupled effects of Si-Al matrix chemistry, interstitial C, initial FCC connectivity, B2-containing heterogeneity, and deformation temperature. Thus, the useful role of Si-containing designs lies in retaining sufficient transformable FCC and distributing FCC→BCC transformation over the yield and strain-hardening stages, rather than simply maximizing the initial BCC fraction.

Heterostructured Fe-rich MEA studies further show that Si- and Al-modified alloy design can be combined with microstructural heterogeneity to improve cryogenic strength–ductility synergy [110].

From a practical perspective, Si-induced Lüders deformation should be treated as a processing- and microstructure-sensitive response. It is useful only when band propagation is accompanied by distributed DIMT, GND accumulation, and HDI hardening rather than persistent strain localization. Potential mitigation strategies include refining or adjusting grain size, retaining a sufficient FCC-connected matrix, and designing heterogeneous FCC/BCC or gradient architectures that distribute strain partitioning over multiple microstructural domains. Therefore, the useful Si pathway is not simply to maximize Lüders deformation or BCC fraction, but to control the onset, propagation, and spatial distribution of the Lüders band so that transformation-assisted hardening does not compromise formability or surface quality.

#### 3.4.4. Interstitial C, B, and N Additions

Although C, B, and N are all interstitial or small-radius additions, their dominant mechanisms are different and should not be treated as equivalent. C mainly operates through interstitial strengthening, carbide precipitation, SFE/FCC-stability tuning, and TWIP/TRIP modification depending on matrix chemistry. B is usually effective at trace levels through solid-solution and grain-boundary or interface-related effects rather than boride precipitation. N can act through solid-solution strengthening, nitride precipitation, local chemical order, SFE tuning, and suppression or modification of FCC→HCP TRIP. Therefore, the following discussion separates C, B, and N according to their dominant design roles.

##### C

For C additions, the dominant effect can shift among interstitial strengthening, carbide precipitation, FCC-stability/SFE tuning, and TWIP/TRIP modification. C can provide interstitial strengthening, modify FCC stability, promote carbide precipitation, or support twinning-dominant deformation depending on the matrix. In Fe_60_Co_15_Ni_15_Cr_9.5_C_0.5_ and Fe_65_Co_12.5_Ni_12.5_Cr_9.5_C_0.5_, both alloys contain recrystallized FCC and Cr-rich carbides after cold rolling and annealing, but Fe_65_ contains a much larger BCC martensite fraction [46]. After annealing at 800 °C, Fe_65_ reaches 360/1060 MPa at 293 K and 1100/1900 MPa at 77 K in YS/UTS, with uniform elongations of 20% and 26%, respectively [46]. 

**Figure 10:**
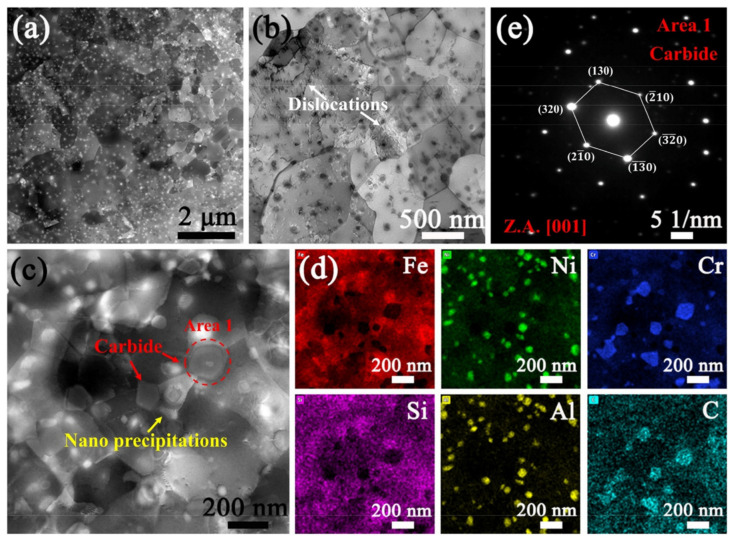
Excess-C-induced carbide precipitation in the annealed (Fe_64_Ni_11_Cr_15_Si_7_Al_3_)_100−x_C_x_ medium-entropy alloy. (**a**) Low-magnification HAADF image showing the distribution of second-phase particles in the dual-phase matrix. (**b**) Bright-field TEM image showing retained dislocations and nanoscale particles. (**c**) High-magnification HAADF image showing carbides and neighboring nanoscale precipitates. (**d**) Fe, Ni, Cr, Si, Al, and C elemental maps, revealing Cr–C enrichment in the carbides and Ni–Al enrichment in adjacent precipitates. (**e**) Selected-area electron diffraction pattern identifying the carbide phase. The formation of brittle Cr_23_C_6_ carbides at the higher C content adversely affects cryogenic deformation compatibility and causes premature fracture during the yield-platform stage. Reproduced from Ref. [100].

A complementary concentration-dependent example is provided by the Co-free (Fe_64_Ni_11_Cr_15_Si_7_Al_3_)_100−x_C_x_ system. After cold rolling and annealing at 1073 K for 10 min, both the C0.25-ANN and C0.5-ANN alloys retain FCC + BCC dual-phase structures. Increasing the C content from 0.25 to 0.5 at.% raises the FCC fraction from approximately 45% to 57% and reduces the average grain size from approximately 910 to 770 nm. The finer structure is partly associated with carbide-induced Zener pinning [100]. However, higher C content also changes the precipitation behavior. As shown in Figure 10, the C0.5-ANN alloy contains pronounced Cr_23_C_6_ carbides in close spatial association with Ni–Al-rich nanoscale precipitates, together with retained dislocations in the dual-phase matrix [100].

This carbide formation produces a pronounced cryogenic penalty. At 77 K, the C0.25-ANN alloy reaches a yield strength of 1550 ± 34 MPa, an ultimate tensile strength of 1926 ± 33 MPa, and a uniform elongation of 25 ± 6%. In contrast, the C0.5-ANN alloy fractures abruptly during the yield-platform stage, so its ultimate tensile strength and uniform elongation cannot be reliably determined. This premature failure is attributed to the limited deformation compatibility between the strong matrix and the brittle carbides at cryogenic temperature [100]. Therefore, increasing C does not produce a monotonic mechanical benefit: moderate C stabilizes FCC and contributes interstitial strengthening, whereas excessive C promotes carbide precipitation and can severely reduce cryogenic damage tolerance.

C does not universally promote martensite or carbides. The cold-rolled Fe_65_(CoNi)_25_Cr_9.5_C_0.5_ alloy shows strong cryogenic performance through FCC→BCC transformation, microstructure refinement, interphase strengthening, and solute strengthening, reaching 1360 MPa YS, 2070 MPa UTS, and 26% ductility [111]. In Fe_49.5_Mn_25_Cr_15_Ni_10_C_0.5_, 0.5 at.% C mainly avoids carbide formation, and the alloy remains single FCC; ductility increases from 49% at 298 K to 71% at 77 K, and TWIP dominates instead of DIMT [12]. Thus, C should be evaluated by matrix chemistry and processing route rather than nominal content alone. In Fe-Mn-Cr-Ni-C alloys, C can remain compatible with a single-FCC, TWIP-dominant pathway [12]. C/Si-alloyed FeMnCoCr provides a different interstitial/metalloid example: C and Si remain dissolved in a single-FCC matrix, and low-temperature strengthening arises mainly from solute strengthening, GND accumulation, and primary/secondary nano-twinning rather than martensitic TRIP [112]. Other C-containing systems further show that C can participate in carbide-assisted heterostructure design, carbide-morphology control, substitutional/interstitial strengthening, boundary strengthening, and processing-tuned FCC→BCC TRIP. FeMnCoCrCuC and Nb-added FeMnCoCrC should be interpreted as M_23_C_6_ dual-heterostructure and Nb_2_C/carbide-morphology examples, respectively [22,23]. Al/C alloying in Co-free Cr_12_Fe_60_Mn_16_Ni_12_ MEAs should be read as substitutional/interstitial, boundary, precipitation, and lattice-distortion/lattice-strain strengthening rather than B/N or nitride evidence [24]. The laser powder bed fusion (PBF-LB) Fe_65_Co_12.5_Ni_12.5_Cr_9.5_C_0.5_ case shows that C-containing Fe-MEAs fabricated by additive manufacturing can be tuned by heat treatment or cold rolling/annealing to exploit FCC→BCC TRIP at cryogenic temperature [113]. Beyond tensile properties, recent studies also show that C can be used to jointly tune phase stability, precipitation, and wear resistance. In a dual-phase Fe_58_Cr_15_Ni_15_V_7_Al_5_C_x_ alloy, C addition shows a non-monotonic effect on phase stability and wear behavior: 0.5 at.% C produces a balanced FCC/BCC matrix reinforced by B2 and VC nanoprecipitates, achieving a UTS of ~1.3 GPa, elongation of 28%, and the best wear resistance, whereas 1 at.% C leads to an FCC-dominant structure and inferior wear resistance [114].

##### B

B should be distinguished from C and N because its reported effect in Fe-based MEAs is mainly trace-level solid-solution and deformation-sequence modification rather than bulk precipitation strengthening. Trace-B-doped Fe_50_Mn_30_Co_10_Cr_10_ is best viewed as solid-solution B plus sequential FCC twinning, FCC→HCP transformation, HCP twinning, and HCP→FCC reverse transformation, not as boride precipitation [21]. A related grain-boundary-engineering example was reported in 30 ppm B-doped Fe_40_Mn_40_Cr_10_Co_10_, where B addition improved the room-temperature tensile response while the dominant deformation mechanism remained essentially unchanged; the alloy reached about 450 MPa YS, 880 MPa UTS, and 73% TE after annealing at 800 °C [48].

When the B content increases, Fe-Mn-Co-Cr-B alloys follow a different route. In arc-melted Fe_50−x_Mn_30_Co_10_Cr_10_B_x_ alloys with x = 0, 0.3, 0.6, and 1.7 wt.% B, B addition changes the microstructural design from a mainly matrix-controlled deformation route toward a boride-containing route [49]. This distinction is important because higher B contents can promote M_2_B-type borides, usually involving Cr and Fe, whereas trace B does not necessarily form a strengthening boride population. Thus, B-containing Fe-based MEAs should not be universally described as either solid-solution-strengthened or boride-strengthened alloys; the primary mechanism depends on the B level and the resulting matrix/boride balance.

The effect of B on deformation mode can be further understood through stacking-fault-energy (SFE) regulation. Experimental and computational analysis of B-doped Fe_50−x_Mn_30_Co_10_Cr_10_B_x_ alloys showed that the average SFE increased from 23.9 ± 2.4 mJ m^−2^ in the B-free alloy to 50.1 ± 14.1 mJ m^−2^ at 5.4 at.% B [50]. This increase indicates that higher B contents may shift the dominant deformation mode from partial-dislocation-mediated twinning and ε-HCP martensitic transformation toward full-dislocation-glide-dominated plasticity. In this sense, B affects not only strengthening but also FCC phase stability, Shockley partial dislocation motion, and the tendency for ε-HCP martensitic transformation.

Processing route further changes the apparent role of B. In laser-cladded Fe_50_Mn_30_Co_10_Cr_10_-based alloys containing 0.1–5.4 at.% B, B addition produced M_2_B-type boride eutectic regions along interdendritic areas, and the microhardness increased from 291 HV to 445 HV as the B content increased to 5.4 at.% [51]. The same study also reported that the high-boride-fraction coating reduced the abrasive wear rate by more than 30% compared with the B-free alloy [51]. This case demonstrates the usefulness of B for surface hardening and abrasive-wear resistance, but it should be interpreted as a coating-specific boride-hardening route rather than as direct evidence for bulk tensile strengthening in wrought Fe-based MEAs.

Overall, B should be regarded as a concentration- and processing-sensitive microalloying element in Fe-based MEAs and related Fe–Mn–Co–Cr-based multi-principal-element alloys. Trace B can improve boundary/interface stability and deformation compatibility without necessarily forming borides. By contrast, moderate or high B contents can promote M_2_B-type borides, increase hardness, and improve wear resistance, especially in coating or surface-modified systems, but they may also suppress transformation-assisted plasticity or introduce brittle interfaces. Therefore, the design target for B-containing Fe-based MEAs is not simply to increase B content, but to maintain a regime in which boundary cohesion, local stability tuning, and TWIP/TRIP compatibility are improved without excessive boride-induced embrittlement.

##### N

N additions can modify phase stability, interstitial strengthening, TWIP, and TRIP behavior, but their mechanisms are strongly dependent on N content, matrix chemistry, and processing route [19,20,52,53]. A B- and N-co-doped CoCrFeMnNi alloy further shows that severe plastic deformation can redistribute BN-related precipitates and generate precipitate-rich/precipitate-lean domains, producing HDI strengthening with a YS of ~1020 MPa and elongation of ~34% [115]. Heavy N-doped FeMnCoCr is a distinct multi-heterostructure case: 2.6 at.% N produces α-martensite laths, deformed austenite, recrystallized ultrafine grains, and nano-nitride precipitates, with HDI strengthening and twinning-dominant deformation [19]. N-supersaturated Fe_50_Mn_30_Co_10_Cr_10_ coatings represent massive N solid-solution strengthening and FCC/HCP stability tuning without nitride formation [20]. For Fe_50_Mn_30_Co_10_Cr_10_, 1.15 at.% N remains in solid solution, suppresses FCC→HCP TRIP, shifts deformation toward TWIP, and increases YS and UE to about 326 MPa and 53.57%, respectively [52]. Heavy N-doped Fe_46.8_Mn_30_Co_10_Cr_10_N_3.2_ shows that interstitial alloying can create interstitial-driven local chemical order (LCO) in fine FCC laths after cold rolling and partial recrystallization, giving about 1.34 GPa YS while retaining UE; this is a LCO/heterostructure-strengthening example rather than a simple carbon-addition case [53]. Therefore, the main effects of N include solid-solution strengthening, nitride precipitation, LCO strengthening, FCC/HCP stability tuning, and TWIP/TRIP pathway modification.

#### 3.4.5. Summary of Al, Ti, Si, C, B, and N Additions

Across these modified systems, Al, Ti, Si, C, B, and N are useful only when stability, precipitation, and deformation mode remain compatible with sustained hardening. They should preserve an FCC-rich or FCC-connected matrix, introduce controllable slip barriers, and allow TRIP, TWIP, HDI hardening, or precipitation strengthening to develop in sequence [17,25,26,99]. Al/Ti/Si/C-modified systems show that minor elements are valuable when they reshape microstructure without destroying strain compatibility [17,25,26,46,109]. To clarify these matrix-dependent effects, the practical elemental design rules for Al, Ti, Si, C, B, and N additions are summarized in Table 6.

Therefore, minor-element modification should not be treated as simple alloying addition, but as a matrix-specific design tool for coordinating phase constitution, precipitation, interstitial effects, and deformation compatibility.

### 3.5. Summary Design Rules for Balancing Stability and Strengthening

The representative systems discussed in Section 3 indicate that composition design in Fe-based MEAs should be treated as stability-window design rather than a simple search for the highest tensile strength. First, the FCC matrix should retain sufficient stability for processing and early plastic deformation, while remaining metastable enough to activate TWIP, FCC→HCP TRIP, or FCC→BCC DIMT during subsequent loading. If transformation occurs too rapidly, cryogenic ductility can be reduced, whereas grain refinement and related microstructural regulation can delay and prolong TRIP [8]. Second, transformation kinetics should be controlled rather than maximized. A gradual and spatially distributed transformation is more useful than a high final martensite fraction, because it continuously introduces new interfaces, sustains work hardening, and delays plastic instability. In Fe-Co-Ni-Cr-Mo systems, the shift from twinning-dominated deformation to transformation-assisted deformation further shows that FCC stability, transformation tendency, and HDI hardening must be balanced rather than optimized separately [82].

Third, alloying additions should be evaluated by their compatibility with the matrix rather than by a fixed elemental role. Fe content, Mn content, Mo partitioning, and minor Al, Ti, Si, and C additions modify the same coupled variables: FCC stability, SFE, phase constitution, precipitation behavior, and sustainable strain-hardening capacity. Al additions can shift Fe-Mn-Ni-Cr-based matrices from single FCC toward FCC + BCC structures, but the strengthening benefit depends on whether sufficient FCC connectivity and deformation compatibility are retained [16]. Si-modified Fe-rich MEAs further show that low-cost FCC + BCC design can activate Lüders deformation, DIMT, and HDI hardening, although excessive strain localization or BCC enrichment may impair formability and damage tolerance [17]. C- and Mo-containing systems indicate that carbide precipitation, interstitial strengthening, and solute partitioning can strengthen the matrix while also modifying residual FCC stability and transformation kinetics [12,18,46,82]. Ti-containing systems and high-pressure-processed FeCrNiTiAl-type MEAs further show that precipitation or TiC-related strengthening is beneficial only when precipitate size, distribution, and matrix deformation compatibility are properly controlled [25,26,27,117]. Therefore, minor-element modification should be treated as a matrix-specific strategy for coordinating phase stability, precipitation, and deformation compatibility rather than as a simple additive effect.

Finally, composition design should be connected to the microstructural and strengthening analyses in the following sections rather than repeated as additional composition-property catalogues. Section 3 defines the main composition-dependent stability routes, whereas Section 4 discusses how grain size, recrystallization state, precipitates, defects, cell structures, and heterogeneous architectures regulate local metastability and deformation pathways. Section 5 further explains how these microstructural states contribute to solute strengthening, precipitation strengthening, TRIP/TWIP-mediated hardening, HDI strengthening, strain-rate response, and service-oriented performance. Therefore, the role of Section 3 is to define composition-based stability windows, while Section 4 and Section 5 explain how these windows are activated, strengthened, or limited by microstructural evolution and deformation mechanisms.

## 4. Microstructural Characteristics and Evolution

**Figure 11:**
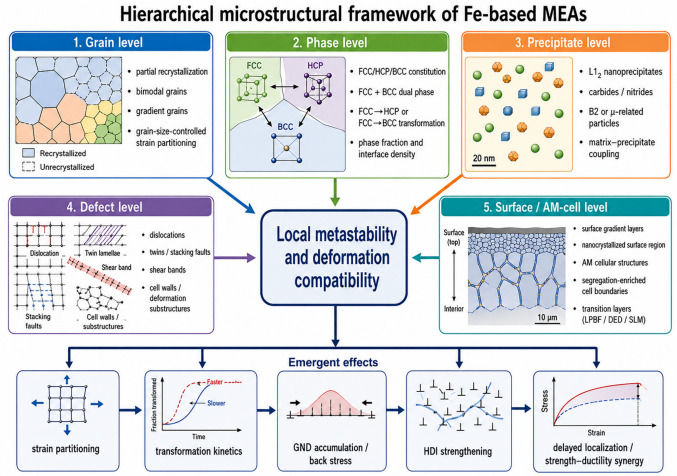
Hierarchical microstructural framework of Fe-based MEAs. The schematic summarizes five structural levels that regulate local metastability and deformation compatibility: grain level, phase level, precipitate level, defect level, and surface/AM-cell level. Grain-level features include partial recrystallization, bimodal grains, gradient grains, and grain-size-controlled strain partitioning. Phase-level features include FCC/HCP/BCC constitution, FCC + BCC dual-phase structures, and FCC→HCP or FCC→BCC transformation. Precipitate-level features include L1_2_ nanoprecipitates, carbides, nitrides, B2 or μ-related particles, and matrix–precipitate coupling. Defect-level features include dislocations, twins, stacking faults, shear bands, cell walls, and deformation substructures. Surface/AM-cell-level features include surface gradient layers, nanocrystallized surface regions, AM cellular structures, segregation-enriched cell boundaries, and LPBF/DED/SLM transition layers. These structural levels collectively affect strain partitioning, transformation kinetics, GND accumulation, back stress, HDI strengthening, delayed localization, and strength–ductility synergy.

Microstructural regulation in Fe-based MEAs operates across multiple structural levels rather than through a single descriptor such as grain size or phase fraction alone. To clarify the relationship between Section 2.3 and Section 5.2, this section discusses the characteristic microstructures of Fe-based MEAs according to a hierarchy of structural levels: grain level, phase level, precipitate level, defect level, and surface/AM-cell level. At each level, the key issue is not only the morphology itself, but also how that structural feature modifies local metastability, strain partitioning, interface density, and deformation compatibility. This hierarchical framework is schematically summarized in Figure 11. For consistency throughout this review, a heterogeneous structure refers to a microstructure containing domains with different mechanical responses, such as differences in grain size, phase constitution, precipitate density, or defect density, so that strain partitioning develops during deformation. A hierarchical structure refers to a special case of heterogeneous structure in which heterogeneity exists simultaneously at two or more structural levels. Partial recrystallization refers specifically to the coexistence of recrystallized grains and unrecrystallized or deformed regions. HDI strengthening refers to hetero-deformation-induced strengthening arising from deformation incompatibility between soft and hard domains, which generates strain gradients, geometrically necessary dislocations (GNDs), back stress, and sustained strain hardening.

### 4.1. Grain-Level Heterogeneity and Partial Recrystallization

**Figure 12:**
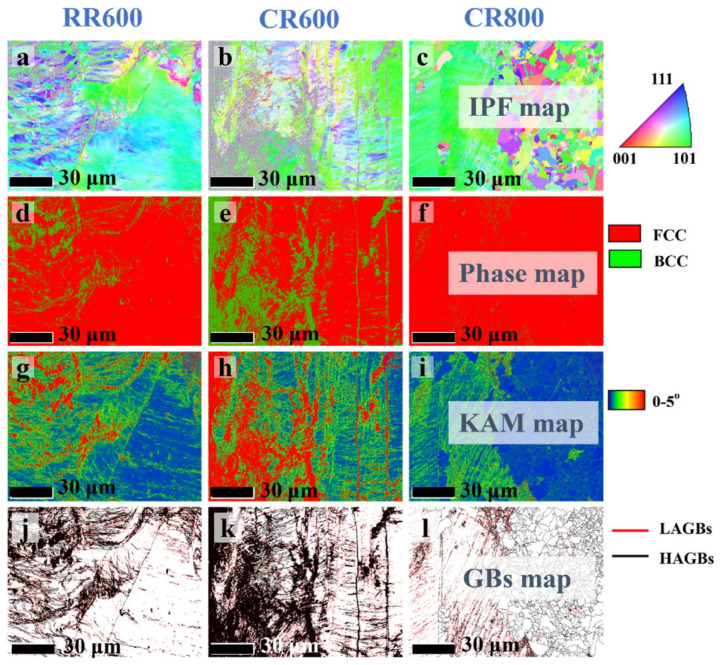
Processing-dependent grain-level heterogeneity and partial recrystallization in the Fe_62_Ni_16_Co_9_Mn_9_Ti_3_Si_1_ alloy. (**a**–**c**) EBSD inverse-pole-figure maps, (**d**–**f**) phase maps, (**g**–**i**) kernel average misorientation maps, and (**j**–**l**) grain-boundary maps of the RR600, CR600, and CR800 conditions before tensile deformation, respectively. RR600 and CR600 retain predominantly deformation-affected microstructures with low recrystallized fractions and relatively high local misorientation, whereas annealing the cryo-rolled alloy at 800 °C produces a partially recrystallized bimodal structure containing fine recrystallized grains and coarse deformed grains. This grain-size and defect-density heterogeneity provides the structural basis for strain partitioning and hetero-deformation-induced strengthening. Reproduced from Ref. [107].

At the grain level, partial recrystallization, bimodal grain-size distributions, and soft-hard grain domains provide a direct route for regulating local metastability, strain partitioning, and HDI strengthening in Fe-based MEAs. Because grain-level heterogeneity often couples with precipitate or phase-level heterogeneity, some examples discussed here also contain nanoscale precipitates or phase-reversion structures. When neighboring regions have different flow strengths, early strain partitioning produces interfacial strain gradients, GND accumulation, and back stress [78,80,93,118]. In partially recrystallized alloys, coarse unrecrystallized regions combined with ultrafine recrystallized grains raise YS while preserving multistage hardening [28,118]. A direct processing comparison is provided by the cryo-rolled and annealed Fe_62_Ni_16_Co_9_Mn_9_Ti_3_Si_1_ alloy, as shown in Figure 12. The RR600 and CR600 conditions remain predominantly unrecrystallized, with recrystallized fractions of approximately 3.5% and 5.4%, respectively. In contrast, increasing the post-cryogenic-rolling annealing temperature to 800 °C raises the recrystallized fraction of the CR800 alloy to approximately 53.1% and produces a pronounced bimodal grain structure. The average sizes of the fine recrystallized grains and coarse deformed grains are approximately 5.3 and 61.7 μm, respectively [107]. The CR800 condition also exhibits a lower average KAM value of approximately 0.69°, compared with 1.36° for RR600 and 2.27° for CR600, indicating substantial recovery and recrystallization while retaining spatial differences in defect density. This coexistence of fine recrystallized grains and coarse deformation-retaining regions provides the soft–hard domain contrast required for strain partitioning, GND accumulation, and HDI strengthening [107].

Similar effects occur when heterogeneous nanoprecipitates are introduced [93] or when phase reversion generates multiscale grain structures [80]. In heterogeneous lamellar structures, soft–hard incompatibility adds HDI stress during plastic flow [78]. Thus, partial recrystallization and bimodal design do more than refine grains; they divide deformation between soft recrystallized grains and hard non-recrystallized domains, thereby sustaining strain partitioning, GND storage, back-stress hardening, and transformation-assisted hardening during deformation [28,78,80,93,118].

More specifically, in a partially recrystallized microstructure, the recrystallized grains and non-recrystallized domains do not contribute to deformation in the same way. The recrystallized grains are relatively soft and contain fewer stored defects, so they accommodate plastic strain at the early deformation stage and provide deformable FCC regions for progressive twinning or martensitic transformation. In contrast, the non-recrystallized domains retain high dislocation density, subgrain boundaries, and deformation bands, making them mechanically harder and more resistant to early plastic flow. The mechanical mismatch between these two domains drives strain partitioning at their interfaces, resulting in GND accumulation and back stress. As deformation proceeds, the constrained non-recrystallized domains can also act as delayed transformation sites because local stress concentration, retained defects, and shear bands promote martensite nucleation. Therefore, the contribution of partial recrystallization is strain-stage dependent: recrystallized grains mainly support early plastic accommodation and progressive transformation, whereas non-recrystallized domains provide constraint, defect storage, back stress, and later-stage transformation-assisted hardening [28,29].

Gradient and multiscale heterostructures extend this principle by increasing interface density and the length scale of strain partitioning [33,58,60,119,120]. In dual-graded heterostructures, GNDs preferentially accumulate at core–shell interfaces, producing higher HDI stress than in homogeneous counterparts [33]. Surface-nanocrystallized gradient structures improve the strength–ductility balance by combining heterogeneous deformation with additional hardening [60], whereas localized gradient layers modify plastic flow through dynamic stress partitioning [58]. In banded multiscale heterostructures containing dense B2 nanoprecipitates, interfacial dislocation pile-up confirms active stress-strain partitioning during deformation [119]; analogous behavior has been observed in fine/coarse grain domains decorated with nanoprecipitates [120]. These architectures mainly maintain strain partitioning, GND accumulation, and back-stress-related hardening over extended strain, delaying strain localization [33,58,60,119,120].

### 4.2. Phase-Level FCC + BCC Dual-Phase Structures

At the phase level, the coexistence or evolution of FCC, HCP, and BCC phases provides a direct way to balance strength and ductility through phase-fraction control, interface-mediated deformation, and strain partitioning. To avoid ambiguity, BCC is used here as a crystallographic descriptor, whereas the origin of the BCC constituent is specified whenever possible. Pre-existing BCC refers to a BCC phase already present in the microstructure before the mechanical test. Thermally induced BCC martensite refers to BCC martensite formed during cooling or heat treatment without external plastic deformation. Deformation-induced BCC martensite refers to BCC martensite formed from metastable FCC during tensile loading, rolling, cryogenic deformation, or other imposed plastic deformation. The term ferrite is used only when the cited work identifies the BCC constituent as a ferritic or thermodynamically stable BCC phase rather than martensite. This subsection focuses mainly on pre-existing FCC + BCC dual-phase structures, whereas dynamically formed deformation-induced BCC martensite is discussed in Section 4.3.

**Figure 13:**
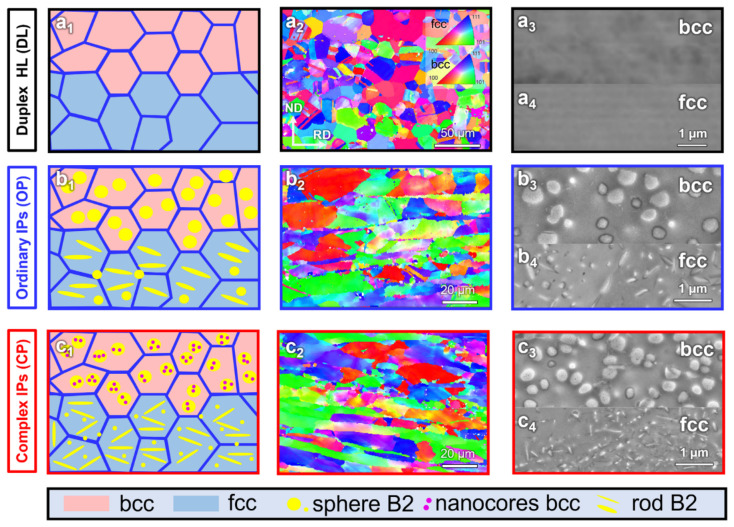
Pre-existing laminated FCC/BCC architectures and precipitate distributions in the Fe_58_Ni_16_Cr_16_Al_10_ medium-entropy alloy. (**a1**–**c1**) Schematic representations of the duplex heterogeneous micro-lamellar structure without pronounced multicomponent intermetallic precipitates (DL), the structure containing ordinary B2 multicomponent intermetallic precipitates (OP), and the structure containing structurally complex core–shell B2 precipitates with dispersed BCC nanocores (CP), respectively. (**a2**–**c2**) Corresponding EBSD inverse-pole-figure maps showing the laminated FCC/BCC grain structures. (**a3**–**c3**) SEM images showing the absence or presence of spherical B2 precipitates within the BCC constituent. (**a4**–**c4**) SEM images showing the absence or presence of rod-like and spherical B2 precipitates within the FCC constituent. The three conditions retain pre-existing FCC/BCC duplex matrices while exhibiting different phase fractions, grain scales, and precipitate morphologies. Reproduced from Ref. [122].

FCC + BCC dual-phase microstructures are widely used to balance strength and ductility in Fe-based MEAs. The FCC/BCC ratio, however, is system-dependent rather than intrinsic. In Fe_65_Co_6_Ni_6_Cr_15_Si_8_, the initial microstructure contains about 64 vol.% FCC together with a pre-existing BCC constituent, whereas Fe_65_Co_10_Ni_10_Cr_15_ has an initial FCC fraction of about 93–94% and therefore a much smaller pre-existing BCC fraction before deformation. Thus, the initial FCC/BCC phase ratio is an adjustable design variable controlling deformation compatibility, not a fixed structural descriptor [17,68,121]. Within these dual-phase structures, phase fraction, morphology, interface density, and local strain partitioning all matter mechanistically. A more direct example of pre-existing FCC + BCC architectural control is provided by the Fe_58_Ni_16_Cr_16_Al_10_ medium-entropy alloy shown in Figure 13. Through different annealing treatments, three laminated duplex microstructures were obtained: a duplex heterogeneous micro-lamellar structure without pronounced multicomponent intermetallic precipitates (DL), a structure containing ordinary B2 multicomponent intermetallic precipitates (OP), and a structure containing complex core–shell B2 precipitates with dispersed BCC nanocores (CP) [122]. All three conditions retain a pre-existing laminated FCC/BCC matrix before mechanical loading, but their phase fractions, grain dimensions, and precipitate populations differ substantially. The FCC fractions are approximately 55%, 72%, and 60% in the DL, OP, and CP conditions, respectively, while the corresponding average FCC grain sizes decrease from approximately 19.2 to 15.1 and 11.8 μm. The BCC grain size is also refined from approximately 29.7 μm in DL to approximately 6.3 and 6.5 μm in OP and CP, respectively [122]. These results demonstrate that FCC + BCC dual-phase design involves not only phase-fraction control, but also phase morphology, grain scale, interface density, and the distribution of strengthening particles within each constituent.

A related but distinct response occurs in Fe_65_Co_6_Ni_6_Cr_15_Si_8_. This alloy contains a pre-existing FCC + BCC structure before deformation, but the increase in BCC fraction and KAM value during loading indicates additional deformation-induced BCC martensite formation and defect accumulation [17].

The mechanical role of BCC depends strongly on its origin. Pre-existing BCC or retained BCC martensite raises flow strength, restricts slip, and provides FCC/BCC interfaces for strain partitioning, whereas the FCC matrix accommodates plastic strain. In metastable Fe-rich systems, additional FCC→BCC transformation may occur during cryogenic or applied loading; this newly formed BCC should be described specifically as deformation-induced BCC martensite because it generates fresh interfaces and additional dislocation obstacles during deformation [17,68,69,111]. In the low-cost (FeNiCr)_94_Ti_2_Al_4_ MEA, Ti and Al additions transform the initial structure from single FCC to FCC + BCC dual phase. The optimized Ti_2_Al_4_ alloy contains about 79% FCC and 21% BCC, and its cryogenic properties are mainly attributed to solid-solution strengthening, BCC phase strengthening, grain-boundary strengthening, dislocation strengthening, and deformation twinning rather than TRIP [121]. In the cold-deformed Fe_65_(CoNi)_25_Cr_9.5_C_0.5_ alloy, the BCC martensite should be interpreted as deformation-induced during prior cold deformation and pre-existing at the beginning of the subsequent mechanical response; the extended strain-hardening stage indicates that a balanced FCC/BCC structure can delay hardening saturation and plastic instability [111]. Therefore, the value of FCC + BCC microstructures lies in phase-fraction control, interface-mediated deformation, and back-stress strengthening rather than simple two-phase coexistence. Whenever BCC forms during the mechanical test itself, it should be identified separately as deformation-induced BCC martensite [17,68,69,111,121].

### 4.3. Phase-Level Transformation-Assisted Structures

In addition to initial FCC + BCC phase coexistence, phase-level heterogeneity can also develop dynamically through deformation-induced transformation. This subsection focuses on metastable FCC-based structures in which FCC→HCP or FCC→BCC transformation changes phase fraction, interface density, and strain partitioning during deformation. Because FCC→HCP TRIP and FCC→BCC DIMT differ in crystallographic pathway, temperature sensitivity, hardening intensity, and ductility risk, they should be distinguished rather than treated as a single transformation-assisted mechanism. Table 7 summarizes their main differences and the corresponding characterization methods used in Fe-based MEAs.

**Figure 14:**
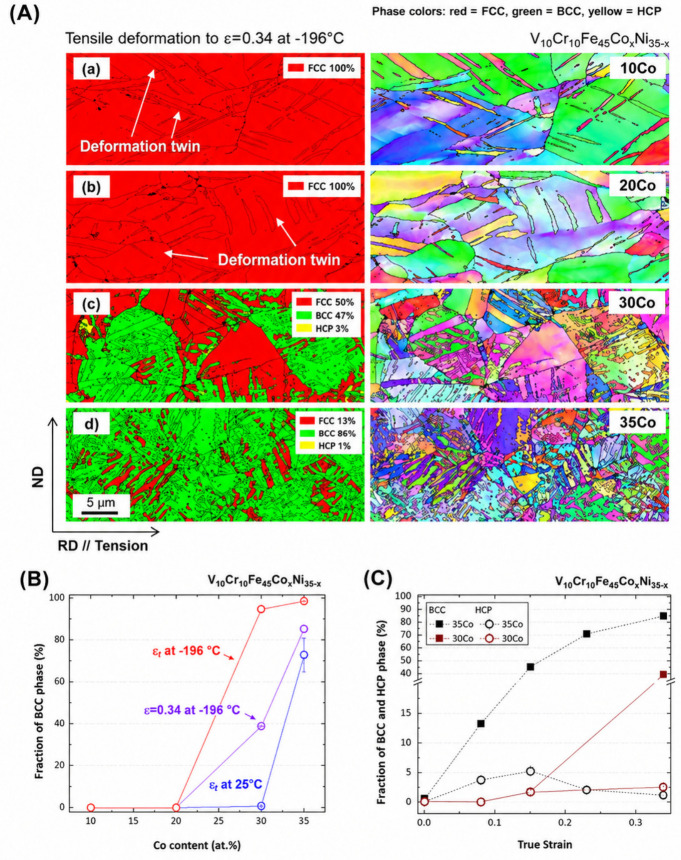
Co-Ni-balance-dependent cryogenic deformation mechanisms and martensitic transformation in the V_10_Cr_10_Fe_45_Co_x_Ni_35−x_ medium-entropy alloys. (**A**) EBSD phase and inverse-pole-figure maps after tensile deformation to a true strain of approximately 0.34 at −196 °C: (**a**) 10Co, (**b**) 20Co, (**c**) 30Co, and (**d**) 35Co alloys. The 10Co and 20Co alloys remain essentially FCC and exhibit deformation twinning, whereas the 30Co and 35Co alloys develop BCC-rich transformed microstructures. (**B**) BCC phase fraction as a function of Co content after room-temperature and cryogenic tensile deformation. (**C**) Evolution of BCC and HCP phase fractions with true strain in the 30Co and 35Co alloys. Increasing Co at the expense of Ni lowers FCC stability, advances the onset of transformation, and promotes a sequential FCC→HCP→BCC transformation pathway. Adapted and recomposed from Ref. [41].

In Fe_50_Mn_25_Cr_12.5_Co_5_Ni_5_Si_2.5_, Lin et al. [123] identified a stable single-FCC starting phase by XRD; yet Si-induced SFE reduction increases transformation susceptibility under cryogenic loading. Under these conditions, twinning is accompanied by TEM-observed ε-martensite laths [123]. This transition matches the broader behavior of metastable Fe-based MEAs. A complementary Co-Ni balance study in the V_10_Cr_10_Fe_45_Co_x_Ni_35−x_ medium-entropy alloy system provides a direct view of the transition from twinning-dominated deformation to BCC-rich transformation-induced plasticity. As shown in Figure 14, the 10Co and 20Co alloys remain essentially FCC after cryogenic deformation to a true strain of approximately 0.34, with deformation twinning serving as the principal additional deformation mode. In contrast, the 30Co and 35Co alloys undergo extensive martensitic transformation, reaching BCC fractions of approximately 47% and 86%, respectively [41]. The strain-dependent phase-fraction curves further show that transformation begins earlier in the 35Co alloy: approximately 13.3% BCC and 3.8% HCP are already present at a true strain of 0.08, whereas pronounced transformation in the 30Co alloy begins near a true strain of 0.15 [41]. The transient increase and subsequent reduction of the HCP fraction indicate that HCP acts primarily as an intermediate nucleation pathway for the progressive FCC→HCP→BCC transformation.

The key low-temperature change is not only the amount of martensite, but also the transformation pathway. In Fe_50_Mn_25_Cr_12.5_Co_5_Ni_5_Si_2.5_, room-temperature deformation is governed mainly by dislocation activity and twinning, whereas cryogenic straining activates FCC→HCP transformation [123]. This FCC→HCP TRIP route is beneficial when ε-HCP formation occurs progressively and cooperates with twinning, because HCP/FCC interfaces and twin boundaries subdivide slip paths and sustain work hardening. A stronger transformation sequence occurs in the V_10_Cr_10_Fe_45_Co_x_Ni_35−x_ series as Co progressively replaces Ni. At cryogenic temperature, the 30Co alloy reaches a tensile strength of approximately 1291 MPa with an elongation of about 81.7%, whereas the more metastable 35Co alloy reaches approximately 1623 MPa but shows a lower elongation of about 65% [41]. This comparison demonstrates that earlier and more extensive BCC formation increases strength, but excessive transformation can reduce the remaining FCC reservoir and diminish ductility. By comparison, Fe_x_Mn_75−x_Ni_10_Cr_15_ also begins from a single-FCC matrix, demonstrating that Co is not required to obtain cryogenic transformation-assisted plasticity. In Fe_50_Mn_25_Ni_10_Cr_15_, FCC→HCP TRIP and TWIP operate cooperatively, producing a tensile strength of approximately 1 GPa while retaining more than 80% ductility at 77 K [11]. Taken together, these alloys represent different regions of the transformation map summarized in Table 7: the Fe-Mn-Ni-Cr system primarily exploits cooperative FCC→HCP TRIP and TWIP, whereas the V_10_Cr_10_Fe_45_Co_x_Ni_35−x_ system progressively shifts toward sequential FCC→HCP→BCC transformation as Co replaces Ni.

By contrast, FCC→BCC/α′ DIMT usually provides stronger hardening but also carries a higher localization risk. Related studies indicate direct strengthening by α′-martensite in Fe-Co-Cr-V- and Fe-Co-Ni-Cr-based alloys [5,67,124], whereas Si accelerates α′ formation and DIMT kinetics in a recrystallized FCC matrix [9]. Deformation-induced BCC/α′ martensite is harder than the parent FCC matrix and can promote phase-stress partitioning and rapid strain hardening. However, if α′ martensite forms too early or too locally, the transformable FCC reservoir is exhausted, stress and strain concentrate around martensitic regions, and uniform elongation decreases. Thus, the design goal is not only to promote transformation, but to control the transformation path, onset strain, and spatial distribution so that FCC→HCP TRIP, FCC→BCC/α′ DIMT, or sequential FCC→HCP→BCC transformation can sustain hardening without causing premature localization [5,6,9,11,67,123,124].

### 4.4. Precipitate-Level Heterogeneity and Matrix–Precipitate Coupling

At the precipitate level, ordered nanoprecipitates, carbides, nitrides, B2 particles, or μ-related particles should be considered not only as strengthening obstacles, but also as microstructural regulators of residual matrix stability, interface density, and transformation behavior. Therefore, this subsection focuses on matrix–precipitate coupling rather than precipitation strengthening alone. Because different precipitates differ greatly in morphology, location, interface character, and matrix effect, their roles should be distinguished before discussing individual alloy examples. Table 8 summarizes the common precipitate types reported in Fe-based MEAs and related Fe-rich concentrated alloys, including their typical morphology, preferred location, strengthening contribution, and potential microstructural risks.

**Figure 15:**
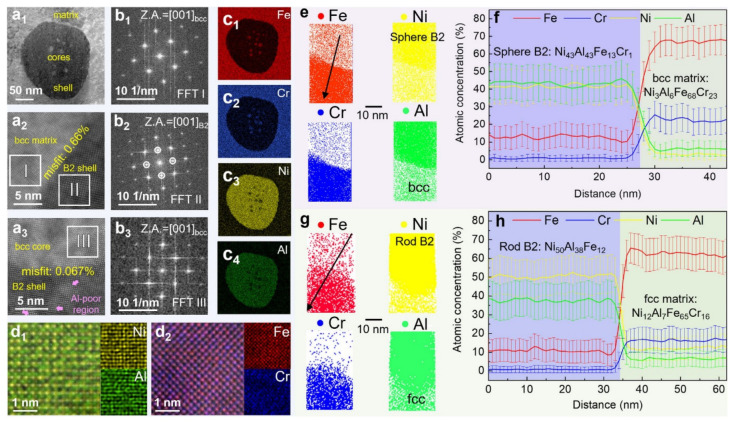
Structurally complex core–shell B2 multicomponent intermetallic precipitates in the BCC constituent of the Fe_58_Ni_16_Cr_16_Al_10_ medium-entropy alloy. (**a1**–**a3**) HAADF-STEM images showing a spherical precipitate, the BCC matrix/B2 shell interface, and the B2 shell/BCC nanocore interface. (**b1**–**b3**) Corresponding FFT patterns identifying the disordered BCC matrix, ordered B2 shell, and disordered BCC nanocore. (**c1**–**c4**) Elemental maps showing an Fe-Cr-rich BCC matrix, a Ni-Al-rich B2 shell, and Fe–Cr-rich internal nanocores. (**d1**,**d2**) Atomic-resolution chemical characterization of the ordered NiAl-type shell and Fe-Cr-rich nanocore. (**e**–**h**) Atom-probe reconstructions and concentration profiles of spherical B2 precipitates in the BCC matrix and rod-like B2 precipitates in the FCC matrix. The core–shell architecture introduces coherent internal interfaces and compositional heterogeneity that enhance load transfer, dislocation storage, and precipitate self-hardening. Reproduced from Ref. [122].

As summarized in Table 8, precipitation-strengthened microstructures in Fe-based MEAs and related Fe-rich concentrated alloys are introduced by aging, annealing, microalloying, or additive-manufacturing-related reactions, but their effectiveness depends on precipitate type, size, distribution, location, and interface character rather than second-phase presence alone. Co-free equiatomic FeCrNiMn provides an annealing-induced precipitate-network example: annealing reduces coarse dendrites and introduces ultrafine precipitate networks, giving about 398 MPa YS and 60.2% elongation at room temperature [130]. Effective precipitation-strengthened designs usually rely on nanoscale ordered precipitates or hard secondary phases with high number density and controlled dispersion. Representative examples include Co-free high-temperature L1_2_-strengthened MEAs [131] and hierarchical FCC + L1_2_ + Cr-rich BCC precipitation-strengthened Co-free MEAs [132]. Fe_40_Ni_33_Co_11_Cr_5_Al_7_Ti_4_ provides a direct Fe-based case, where nanosized L1_2_ precipitates embedded in a heterogeneous grain distribution generate high YS and HDI-assisted hardening, making this alloy a precipitation-plus-heterogeneous-grain-distribution example [133]. A separate Fe_57.88_(CrNi)_32.08_Al_10.04_ study shows that solution treatment followed by aging increases B2 precipitation in an FCC matrix and raises YS, representing heat-treatment-driven B2 precipitate strengthening in Fe-Cr-Ni-Al MEAs [127]. A more structurally complex precipitation route is demonstrated by the Fe_58_Ni_16_Cr_16_Al_10_ medium-entropy alloy. As shown in Figure 15, the BCC constituent contains spherical core–shell B2 multicomponent intermetallic precipitates composed of a Ni-Al-rich ordered B2 shell and dispersed Fe-Cr-rich disordered BCC nanocores [122]. High-resolution characterization confirms a cube-on-cube orientation relationship across both the BCC matrix/B2 shell and B2 shell/BCC nanocore interfaces, with lattice misfits of approximately 0.66% and 0.067%, respectively. Elemental mapping and atom-probe analysis further reveal pronounced compositional partitioning among the BCC matrix, B2 shell, and internal nanocores [122]. This hierarchical architecture generates multiple coherent interfaces, local elastic-strain fields, and chemical-energy fluctuations, allowing the precipitates to act not only as dislocation obstacles but also as internal dislocation-storage and multiplication sites. 

Unlike conventional chemically homogeneous ordered precipitates, the core–shell B2 particles provide a hierarchical internal structure that modifies both strengthening and deformation behavior. The coherent interfaces obstruct dislocation motion and contribute back stress, while the disordered nanocores and chemical heterogeneity within the B2 shell promote dislocation emission, multiplication, and tortuous slip. Consequently, the precipitates can retain high load-bearing capacity without exhibiting the severe glide-plane softening commonly associated with uniformly ordered intermetallic particles [122].

Although not a classical precipitate-strengthened case, a recent Mo-doped FCC Fe-based MEA provides a useful contrast by showing that matrix chemistry can also couple strength, FCC→BCC transformation, and passive-film stability. The (Fe_62_Co_8_Ni_15_Cr_15_)_97_Mo_3_ alloy achieves a balanced strength–ductility–corrosion response, with 600 MPa YS and 26% UE at 298 K, 990 MPa YS and 42% UE at 77 K, and a pitting potential of ~0.83 V in 3.5 wt.% NaCl solution [91]. In Fe_45_Co_35_Cr_10_V_10_, phase reversion and partial recrystallisation produce ultrafine recrystallized FCC grains, twinning, and DIMT [134], whereas controlled aging of the recrystallized FCC state provides BCC/B2 and σ precipitation control [129]. In Fe_41.9_Ni_34.4_Co_11.3_Cr_2.3_Al_6.9_Ti_3.2_, nanolamellar L1_2_ precipitation and solute partitioning left a metastable FCC matrix, giving about 1.44 GPa YS, 1.64 GPa UTS, and useful ductility through precipitation plus TRIP [125]; this response reflects precipitation hardening coupled with TRIP [125].

The examples above mainly represent coherent or semi-coherent nanoscale precipitation in an FCC matrix. The following examples further show that precipitate morphology and location can change from intragranular spherical particles to grain-boundary rods, lamellae, B2 particles, or σ-related products, leading to different combinations of precipitation strengthening, matrix partitioning, and ductility risk. Joseph et al. [135] showed that Fe-rich alloys can be designed within a γ + γ′-only phase field, namely an FCC γ matrix containing ordered L1_2_ γ′ precipitates; after aging, the alloy contained both spherical intragranular γ′ precipitates and rod-like grain-boundary γ′ precipitates. Kim et al. [126] showed that, in a harmonic core–shell structure, continuous precipitation forms spherical intragranular L1_2_ precipitates, whereas discontinuous precipitation forms rod-like or lamellar L1_2_ precipitates at shell grain boundaries [126]. In the B2-strengthened route, Gao et al. [128] studied a Fe-18Ni-16Cr-5Al-1Mn wt.% microlaminated FCC/BCC duplex MEA and identified two B2 nanoprecipitate populations of ~43.1 nm in BCC and ~38.9 nm in FCC; the BCC/B2 interface was nearly coherent (misfit ~0.19%), whereas the FCC/B2 interface showed a higher misfit (~3.19%) and a Kurdjumov–Sachs (K-S) orientation relationship [128]. A recent structurally complex B2-precipitate design further shows that core–shell nanoprecipitates can suppress glide-plane softening and improve strength–ductility synergy by acting as both dislocation obstacles and dislocation sources [122]. More broadly, precipitate-level design should balance three factors: obstacle strength, matrix compatibility, and chemical side effects. L1_2_ and B2 precipitates are beneficial when coherency or low interface misfit allows dislocation storage without severe cracking; μ phase, σ phase, and coarse carbides can provide high strength but may also introduce brittle interfaces; and carbide or nitride formation can alter matrix chemistry through Cr, Mo, Ti, C, or N partitioning. Therefore, precipitate-strengthened Fe-based MEAs should be evaluated not only by yield-strength increments, but also by whether precipitation preserves FCC connectivity, controlled metastability, corrosion resistance, and ductility.

### 4.5. Hierarchical Heterogeneity and Defect-Assisted HDI Strengthening

The preceding subsections separately discuss grain-level, phase-level, and precipitate-level microstructures for clarity, but many Fe-based MEAs contain coupled heterogeneity across several structural levels. Such microstructures are referred to here as hierarchical structures. In these cases, HDI strengthening is usually assisted by defect-level responses, including GND accumulation, dislocation pile-up, back stress, and deformation incompatibility across soft and hard domains, Heterogeneous Fe-Ni-Co-Mn-Ti-Si states show that HDI strengthening and mechanically induced martensite/austenite incompatibility improve strength–ductility balance [136]. In related precipitation-strengthened Fe-rich systems, heterogeneous L1_2_ precipitation, harmonic core–shell architecture, and multiscale soft–hard partitioning can combine strengthening with large elongation [55,126]. HPT and annealing of Fe-15Ni-8Mn-8Co-3Ti-1Si produce a metastable FCC matrix decorated with Ti-Si-rich precipitates, where grain-boundary engineering and precipitate strengthening delay DIMT and sustain multistep hardening [137]. The hierarchical Co_21_Cr_12.5_Fe_55_Ni_4_Mo_7.5_ alloy demonstrates that Lüders-band-assisted deformation can regulate the timing of massive hardening in an ultrastrong Fe-rich MEA [14].

**Figure 16:**
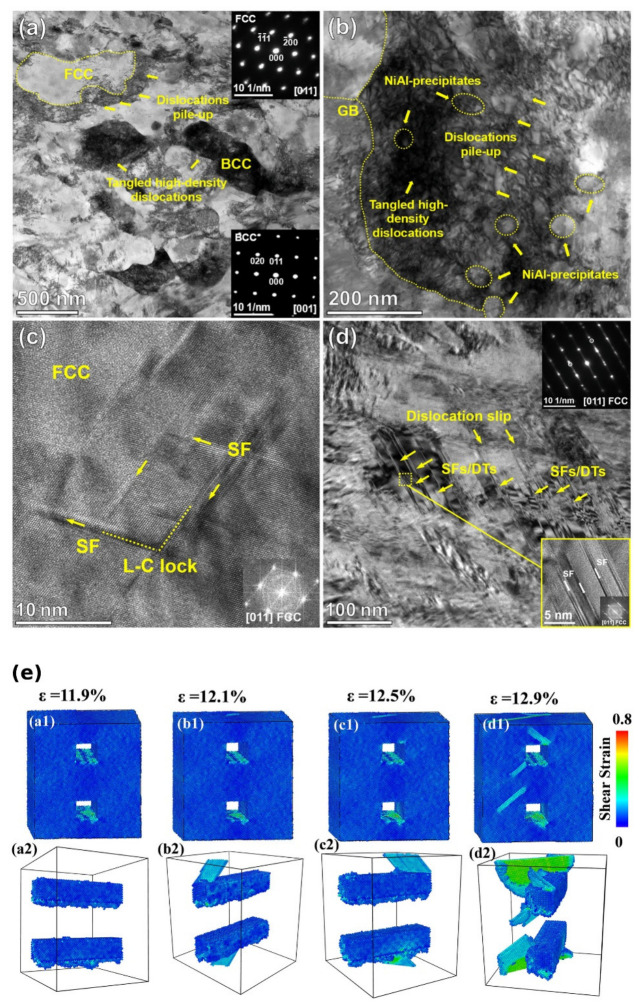
Interfacial defect accumulation and local stress partitioning in the hierarchically heterogeneous Fe_62_Ni_15_Cr_13_Si_7_Al_3_ medium-entropy alloy during cryogenic deformation. (**a**) TEM image showing high-density dislocation entanglement and dislocation pile-up near the FCC/BCC interface. (**b**) NiAl-rich B2 nanoprecipitates and associated dislocation pile-up within the heterogeneous matrix. (**c**) Lomer-Cottrell locks accompanied by stacking-fault networks. (**d**) Stacking-fault and deformation-twin bundles formed during tensile deformation at 77 K. (**e**) Molecular-dynamics results at different tensile strains: (**a1**–**d1**) shear-strain distribution maps at ε = 11.9%, 12.1%, 12.5%, and 12.9%, respectively, and (**a2**–**d2**) the corresponding microstructural-evolution maps, showing local shear-strain concentration and the progressive formation and propagation of stacking faults and deformation twins around the B2/matrix interfaces. The interfacial pile-up, defect interactions, and local stress concentration provide the microstructural basis for back-stress strengthening and sustained strain hardening. Adapted and recomposed from Ref. [110].

Gradient and multiscale heterostructures extend this principle by increasing interface density and distributing strain partitioning across scales. A direct interface-scale example is provided by the partially recrystallized Fe_62_Ni_15_Cr_13_Si_7_Al_3_ medium-entropy alloy. Its hierarchical microstructure combines recrystallized and non-recrystallized regions, FCC/BCC phase heterogeneity, high-density defects, annealing twins, and NiAl-rich B2 nanoprecipitates [110]. As shown in Figure 16, tensile deformation at 77 K produces pronounced dislocation pile-up and entanglement at FCC/BCC and B2/matrix interfaces, together with Lomer–Cottrell locks, stacking-fault networks, and deformation-twin bundles. Molecular-dynamics simulations further reveal local shear–strain concentration around the B2/matrix interfaces and the subsequent nucleation and propagation of stacking faults and deformation twins [110]. These observations demonstrate that multilevel interfaces do not merely obstruct slip; they redistribute local stress, store dislocations, and activate additional defect-mediated deformation mechanisms, thereby supporting back-stress strengthening and sustained strain hardening.

Consistent with this interface-controlled response, the back-stress contribution at yielding was estimated to be approximately 360 MPa at 298 K. Including this contribution increases the calculated yield strength from approximately 720 to 1080 MPa, close to the experimentally measured value of about 1 GPa [110]. These quantitative results confirm that back stress is a major strengthening contribution rather than a secondary correction in this hierarchical microstructure.

Surface severe plasticity and related surface-modification strategies further show that near-surface grain refinement, high dislocation density, and locally enhanced transformation tendency can introduce additional strain gradients and back-stress/HDI strengthening [58]. Other processing-induced heterogeneous or gradient-like structures also demonstrate that spatially heterogeneous deformation can promote strain partitioning and delay strain localization [60,120]. In the banded multiscale heterostructure [119], dense B2 nanoprecipitates are superimposed on structural heterogeneity; superior properties were attributed to combined HDI strengthening, ultrafine-grain strengthening, and precipitation strengthening, and interfacial dislocation pile-up confirmed active stress–strain partitioning. The key advantage of gradient and multiscale heterostructures is dynamic rather than static: they prolong strain partitioning, GND accumulation, back-stress/HDI strengthening, and work hardening, thereby delaying strain localization [33,58,60,119,120]. Partially recrystallized Fe_50_Mn_30_Co_10_Cr_10_ with HDI, FCC→HCP transformation, and twins [138] and NbC-reinforced FeMnCoCr with back-stress strengthening and TRIP [139] both show excellent mechanical properties.

**Figure 17:**
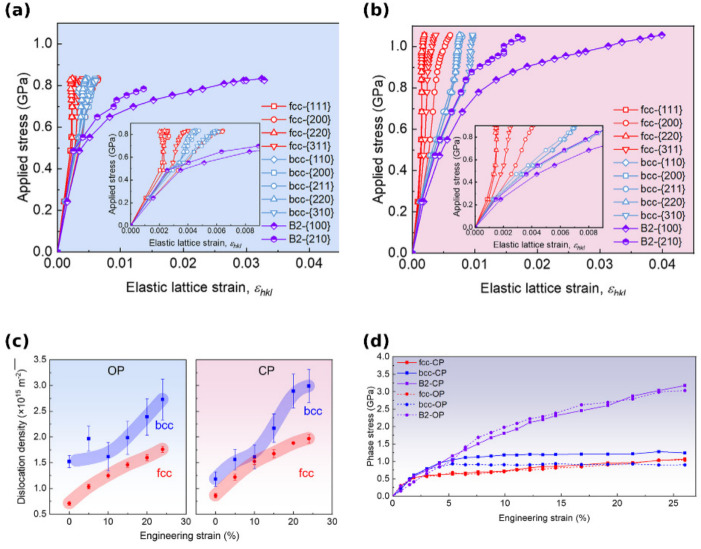
In situ evolution of lattice strain, dislocation density, and phase-stress partitioning in the laminated FCC/BCC Fe_58_Ni_16_Cr_16_Al_10_ medium-entropy alloy. (**a**,**b**) Applied stress as a function of elastic lattice strain for representative FCC, BCC, and B2 crystallographic plane families in the ordinary-precipitate (OP) and structurally complex core–shell-precipitate (CP) conditions, respectively. (**c**) Evolution of FCC and BCC dislocation densities with engineering strain in the OP and CP conditions. (**d**) Macroscopic stress–strain response and corresponding stress partitioning among the FCC, BCC, and B2 constituents. The FCC phase yields first and transfers load to the BCC matrix and B2 precipitates, while the enhanced BCC dislocation accumulation in the CP condition sustains phase-level load partitioning and strain hardening. Adapted and recomposed from Ref. [122].

A quantitative view of dynamic stress partitioning is provided by in situ neutron diffraction of the laminated FCC/BCC Fe_58_Ni_16_Cr_16_Al_10_ medium-entropy alloy. As shown in Figure 17, both the ordinary-precipitate condition (OP) and the core–shell-precipitate condition (CP) exhibit sequential yielding among the FCC, BCC, and B2 constituents. The FCC phase yields first and progressively transfers load to the relatively hard BCC matrix and B2 precipitates [122]. Although the two conditions exhibit similar initial phase-partitioning behavior, the CP alloy develops a markedly stronger dislocation-storage response in the BCC constituent. Its BCC dislocation density increases sharply at engineering strains of approximately 15–20% and reaches about 2.9 × 10^15^ m^−2^ [122]. The corresponding phase-stress evolution confirms that the B2 precipitates and BCC matrix continue to sustain high stresses after FCC yielding, thereby maintaining load transfer and strain hardening.

These results demonstrate that HDI-related strengthening in hierarchical alloys is a dynamic process rather than a fixed strengthening increment. At the early stage, elastic and plastic incompatibility between the relatively soft FCC phase and the harder BCC/B2 constituents initiates load redistribution. With increasing strain, continued BCC dislocation multiplication and the high load-bearing capacity of the B2 precipitates preserve the phase-strength contrast and delay strain localization. Thus, hierarchical FCC/BCC/B2 architectures can sustain hardening through the coordinated evolution of phase stress, dislocation storage, and interface-mediated load transfer [122].

High performance usually comes from mechanisms activated across different scales and strains. Related examples include multiphase FeNiCoAlTaC with FCC, LCO, B2, and TaBC across temperatures [31], and Fe_60_Ni_23.2_Cr_10_Al_3_Ti_3_Nb_0.5_C_0.3_ with harmonic grains, heterogeneous L1_2_ precipitation, and HDI hardening [126]. In situ characterization clarifies this temporal hierarchy. In partially recrystallized Fe_57.5_Co_18_Cr_13_Ni_7.5_Mo_3_C_1_, synchrotron XRD shows that the dominant hardening contribution shifts from DIMT in the recrystallized domain to DIMT in the non-recrystallized domain as deformation proceeds [29].

### 4.6. Surface/AM-Cell Level Heterogeneity and Processing-Dependent Evolution

At the surface/AM-cell level, processing introduces localized heterogeneity through surface gradient layers, nanocrystallized surface regions, segregation-enriched cell boundaries, AM cellular structures, and transition layers. These features tune local metastability and deformation compatibility without necessarily changing the nominal bulk composition. Therefore, processing-dependent microstructural evolution is discussed here mainly as a route for creating surface- and AM-cell-level heterogeneity. To replace a route-by-route narrative with a clearer comparison, Table 9 summarizes the major processing routes in terms of microstructural signatures, metastability effects, mechanical benefits, and remaining limitations. 

As summarized in Table 9, processing routes regulate Fe-based MEAs by changing defect density, grain morphology, phase fraction, chemical segregation, and local transformation tendency. Cold rolling provides a direct way to track γ→ε→α′ and γ→α′ pathways, martensite variant selection, shear banding, and micro-texture evolution during deformation [140]. Warm rolling of Fe_50_Mn_30_Co_10_Cr_10_ improves wrought-sheet integrity by promoting dislocation slip and suppressing excessive martensitic transformation or twinning at intermediate temperatures, while optimized two-stage rolling can refine grains, promote FCC→HCP transformation and Σ3 twins, and preserve elongation [141,142]. SLM processing of Si-added Fe_50_Mn_30_Co_10_Cr_10_ illustrates an AM-based composition/printability route in which dislocation activity, TWIP/TRIP, and phase-transformation-assisted hardening jointly improve strength–ductility balance [145]. Multi-directional forging followed by annealing modifies FCC/HCP phase fractions and grain size, providing a grain-boundary-strengthening and TRIP/TWIP route [143]. Warm cross-rolling followed by critical recrystallization annealing introduces hierarchical nanotwins that store dislocations, refine grains, and delay TRIP activation [144]. Cold rolling followed by partial recrystallization of Fe_50_Mn_30_Co_10_Cr_10_ produces a heterostructure in which HDI strengthening, FCC→HCP transformation, and deformation twins jointly improve strength–ductility balance [138]. DED-fabricated Fe_45_Mn_35_Co_10_Cr_10_ further shows that Mn-segregation-induced chemical boundaries create SFE heterogeneity, coordinating dislocation slip, TRIP, and TWIP to obtain high ductility [146].

AM and laser-based routes provide a more localized version of the same principle. LPBF and laser surface treatment can tailor deformation behavior by introducing cell-boundary segregation, post-AM cell restructuring, and surface cellular structures; when local metastability is properly activated, these heterogeneities enhance back-stress hardening, strain-hardening capacity, and coupled TRIP/HDI response [57,88,147,148]. LPBF can also fabricate periodically layered Fe40/Fe80 FCC/BCC heterostructures in a FeCoCrNiMn/Fe mixed-powder system, where Fe40 (Fe_40_Co_15_Cr_15_Ni_15_Mn_15_) forms FCC-rich soft layers and Fe80 (Fe_80_Co_5_Cr_5_Ni_5_Mn_5_) forms BCC-rich hard layers. The soft/hard layer interfaces generate stress gradients, HDI strengthening, and additional strain hardening [149]. In LPBF Fe_60_(CoCrNiMn)_40_, tuning the volume energy density (VED) adjusts the FCC/BCC phase fraction and produces a printable FCC/BCC heterostructure with high elongation; this case concerns AM processability and VED-controlled phase heterogeneity rather than conventional wrought Fe-MEAs [150]. Therefore, processing should be evaluated not only by whether it increases strength, but also by whether it distributes strain, controls transformation kinetics, and avoids processing-induced defects or localized instability.

Taken together, Section 4 shows that microstructural design in Fe-based MEAs should be understood as hierarchical control of deformation compatibility. Grain-level structures regulate the spatial distribution of strain and transformation; phase-level structures determine FCC/HCP/BCC compatibility and transformation pathways; precipitate-level structures couple obstacle strengthening with matrix-stability modification; defect-level structures control dislocation storage, GND accumulation, and martensite nucleation; and surface/AM-cell-level structures introduce localized metastability and strain partitioning. These levels are not independent. A hierarchical structure is beneficial only when heterogeneity at different scales cooperates to distribute plastic strain, sustain work hardening, and delay damage localization. This framework provides the microstructural basis for the strengthening mechanisms discussed in Section 5.

## 5. Strengthening–Deformation–Property Relationships

In Fe-based MEAs, structure, mechanism, and property are tightly linked: composition and processing set phase stability, grain scale, defect state, and precipitation; these features govern solid-solution, boundary, dislocation, precipitation, TRIP/TWIP, HDI, and back-stress hardening, thereby determining tensile, cryogenic, impact, fatigue, and corrosion performance.

### 5.1. Solute and SFE Engineering

Solutes strengthen the lattice and shift phase stability. In Fe-based MEAs, stacking-fault energy should be regarded as a local, temperature-dependent, and composition-dependent descriptor rather than a universal predictor of deformation mode. A single nominal SFE value can be misleading because the effective stability of the FCC matrix is also affected by local chemical fluctuation, short-range order, elemental partitioning, temperature, strain state, grain size, and defect density. Recent crystal-plasticity simulations on CrCoNi MEAs further support this view: short-range order can be incorporated as a local resistance term affecting slip-system hardening, slip resistance, and saturation stress; increasing the degree of SRO raises indentation hardness and enhances the heterogeneity of shear strain and geometrically necessary dislocation distributions, indicating that local chemical order should be treated as an important microstructural variable in SFE- and slip-mediated deformation analysis [151]. Therefore, SFE is most useful when interpreted together with Gibbs free-energy difference, local matrix chemistry, phase constitution, and transformation kinetics. In practical alloy design, the key question is not whether the calculated SFE falls within a fixed numerical range, but whether the local FCC matrix remains stable enough for early plastic deformation and metastable enough to activate TWIP, FCC→HCP TRIP, or FCC→BCC DIMT progressively during loading. In Fe_52−x_Mn_27_Cr_15_Co_6_Si_x_, the Si-induced strength increment reflects lattice-distortion strengthening coupled with metastability control [152]. Mo and W further illustrate that the balance between dissolved solutes and second-phase partitioning controls the dominant strengthening route. In Fe-Mn-Al-Ni-C ferrous MEAs, Mo contributes through solid-solution and carbide strengthening [153]. In[Fe_64_(CoCrNi)_36_]_100−x_W_x_, the calculated solid-solution strengthening contribution increases from 89 MPa in the 0W alloy to 194 MPa in 1W and 253 MPa in 2.5W, but decreases slightly to 240 MPa in 4W as an increasing fraction of W partitions into W-rich precipitates [154]. This trend indicates that alloying additions cannot be classified as purely solute or precipitation strengtheners because their dominant role changes with matrix solubility and partitioning behavior.

**Figure 18:**
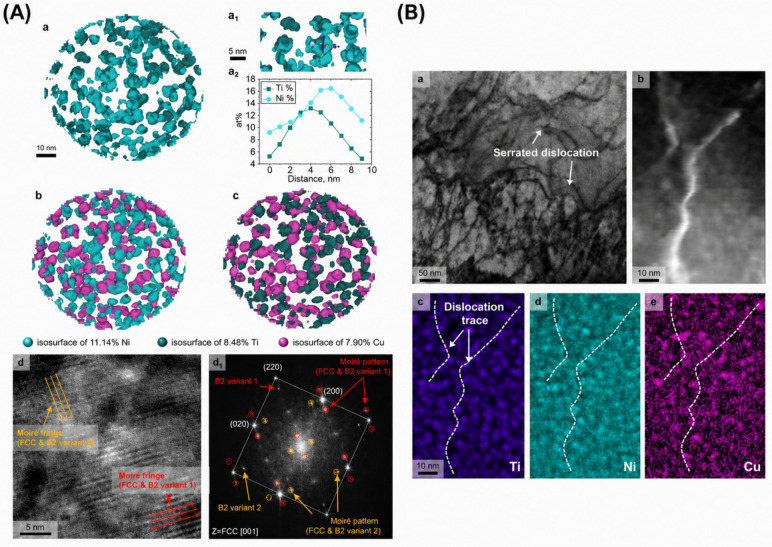
Periodic chemical modulation and dislocation–spinodal-structure interactions in the aged Fe_61.75_Ni_14.25_Co_7.6_Mn_7.6_Ti_2.85_Si_0.95_Cu_4.5_Al_0.5_ medium-entropy alloy. (**A**) Atom-probe tomography (APT) and high-resolution TEM characterization of the periodically spinodally decomposed FCC/B2 nanostructure: (**a**) top-down reconstructed APT map of the Ni–Ti distribution; (**a1**) magnified view of the region in (**a**); (**a2**) Ni–Ti composition profile obtained from the cylindrical region in (**a1**); (**b**) reconstructed APT map of the Ni–Cu distribution; (**c**) reconstructed APT map of the Cu–Ti distribution; (**d**) HRTEM image of the matrix showing moiré fringes; and (**d1**) corresponding FFT pattern of the FCC matrix along the [001] zone axis, showing moiré patterns associated with overlapping B2 variants. (**B**) Dislocation interactions with the chemically modulated nanostructure at a local true strain of approximately 2%: (**a**) STEM bright-field image showing wavy and serrated dislocations; (**b**) STEM dark-field image; and EELS elemental maps of (**c**) Ti, (**d**) Ni, and (**e**) Cu, revealing the dislocation trace through the periodically modulated structure. The periodic compositional fluctuations and associated coherency-strain fields impede dislocation motion and provide a substantial spinodal-hardening contribution while limiting strain localization. Adapted and recomposed from Ref. [103].

A more direct atomic-scale example of chemical-fluctuation strengthening is provided by the aged Fe_61.75_Ni_14.25_Co_7.6_Mn_7.6_Ti_2.85_Si_0.95_Cu_4.5_Al_0.5_ medium-entropy alloy. As shown in Figure 18, aging produces a periodic spinodally decomposed structure containing Fe-rich and Cu-rich FCC regions together with Ni(Ti,Si)-enriched B2 regions [103]. Atom-probe tomography reveals sinusoidal compositional fluctuations with a characteristic wavelength of approximately 11 nm, while high-resolution microscopy confirms the coherent nanoscale modulation. During subsequent deformation, dislocations develop wavy and serrated configurations as they interact with the periodic chemical and elastic-strain fields. The calculated spinodal-hardening contribution is approximately 327 MPa, substantially larger than the approximately 138 MPa contribution from conventional precipitation strengthening [103]. Consequently, aging increases the yield strength from approximately 583 to 1087 MPa while retaining a total elongation of approximately 28.5%.

Interstitial additions also couple strengthening and stability. In B-doped Fe_60_Co_15_Ni_15_Cr_10_, B forms Cr_2_B and depletes Cr from the FCC matrix; the direct precipitation increment is small, whereas matrix destabilization enhances room-temperature TRIP. Thus, this case is better interpreted as B/Cr_2_B-induced matrix modification than as simple boride strengthening [155]. Overall, solutes set the chemical and mechanical basis for TRIP, TWIP, precipitation, and heterostructure hardening, but their effects should be interpreted through local matrix stability rather than through a single nominal SFE value.

### 5.2. Defect, Interface, and Precipitation Strengthening

In Fe-based MEAs, defect, interface, and precipitation strengthening often operate together, but their roles should be separated before discussing individual examples. In this section, direct strengthening refers to mechanisms that directly impede dislocation motion, including grain-boundary strengthening, dislocation strengthening, interface strengthening, and particle strengthening. Indirect effects refer to microstructural or chemical changes that modify deformation pathways, such as matrix destabilization, altered SFE or ΔG, martensite-nucleation-site redistribution, and enhanced TRIP/TWIP activity. This distinction is important because some precipitates or defects contribute only a small direct strengthening increment but strongly change the residual matrix stability and transformation kinetics. Grain boundaries raise yield strength by impeding slip transfer, with boundary character also important. In Fe-25Mn-5Co-12.5Cr-5Ni-2.5Si (in at.%), Lin et al. [123] obtained an intrinsic strengthening term of about 350 MPa and a Hall-Petch coefficient of about 640 MPa·μm^−1/2^. In Fe_50_Mn_30_Co_10_Cr_10_, increasing low-Σ coincidence site lattice (CSL) and Σ3 boundaries disrupts the random high-angle network, suppresses grain-boundary sliding/dynamic recrystallization, and raises 1073 K elongation from about 100% to about 235% without strength loss [156].

Refined grains also modify apparent stability, nucleation-site density, and transformation kinetics. TRIP after grain refinement depends on SFE and matrix stability; a strong TRIP response can persist when the initial SFE is appropriate [157]. Fe_50_Mn_30_Co_10_Cr_10_ shows this structure-mechanism link [54]. Reducing grain size from about 200 μm to about 4 μm raises cryogenic UTS from 1133 to 1342 MPa, while the HCP fraction increases from about 39% at 298 K to about 79% at 77 K. A related study shows that A600 (Fe_50_Mn_30_Co_10_Cr_10_ annealed at 600 °C) is fine-grained single FCC with higher YS, whereas A900 (Fe_50_Mn_30_Co_10_Cr_10_ annealed at 900 °C) is coarser FCC/HCP; both retain TE above 50%, but harden through different combinations of FCC→HCP transformation, slip, reverse transformation, and kink-band formation [158].

Grain size also controls martensite nucleation sites. In Co_18.5_Cr_12_Fe_55_Ni_9_Mo_3.5_C_2_, fine-grained microstructures favor martensite nucleation in non-recrystallized regions and along grain boundaries, whereas coarse-grained states shift nucleation to in-grain shear bands and their intersections [13]. Grain refinement therefore triggers a structural cascade rather than only a Hall–Petch increment.

Dislocation strengthening depends on defect architecture, not only density. In Co_21_Cr_12.5_Fe_55_Ni_4_Mo_7.5_, short annealing after cold rolling preserved non-recrystallized substructures, ultrafine recrystallized grains, and high dislocation content; grain boundaries and martensite interfaces then supplied additional deformation obstacles, so hardening did not rely only on stored defects [14]. DED-processed Fe_60_(CoNi)_30_Cr_10_ confirms the role of local defect architecture. The cellular state has higher average KAM (1.11° versus 0.85°), higher low-angle grain boundary (LAGB) fraction (55.7% versus 34.5%), and higher dislocation density than the cell-free state, linking dense cell networks to stronger HDI strengthening [63].

**Figure 19:**
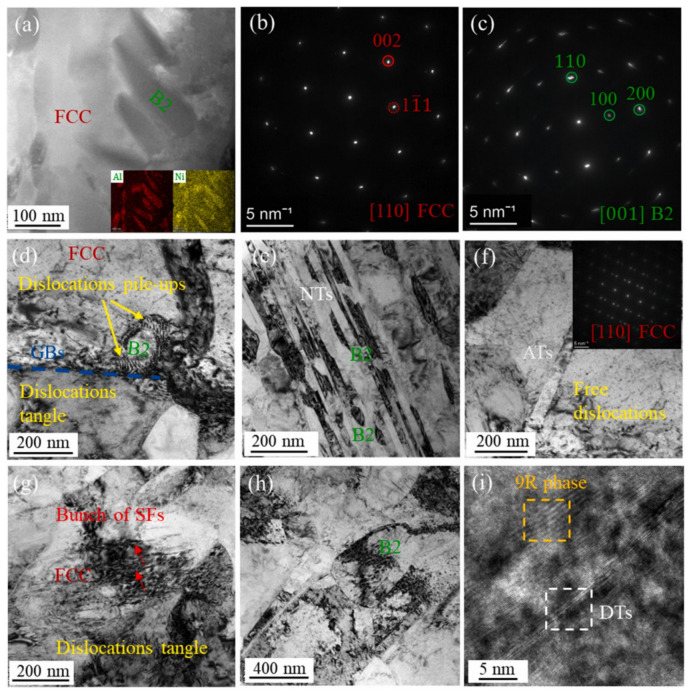
Strain-dependent defect and interface interactions in the cryogenically rolled and annealed Fe_34.95_Ni_28_Co_17.5_Al_11.5_Cr_8_B_0.05_ high-entropy alloy. (**a**–**f**) Microstructural evolution of the CTR90-800 alloy at a tensile strain of 5% at room temperature: (**a**) HAADF-STEM image and corresponding Ni and Al elemental maps showing the FCC/B2 dual-phase structure; (**b**,**c**) selected-area electron diffraction patterns of the FCC matrix and B2 phase, respectively; (**d**) dislocation pile-ups and tangles around FCC/B2 grains; (**e**) retained nanotwins; and (**f**) annealing twins. (**g**–**i**) Deformation structures at a tensile strain of 15%: (**g**) stacking-fault bundles and dislocation entanglement in the FCC matrix; (**h**) high-density dislocations in and around B2 grains; and (**i**) newly formed deformation twins associated with a 9R phase. The progressive interactions among dislocations, twin boundaries, stacking faults, and phase interfaces provide defect storage, interface strengthening, and sustained strain hardening. Reproduced from Ref. [159].

A complementary interface-resolved example is provided by the cryogenically rolled and annealed Fe_34.95_Ni_28_Co_17.5_Al_11.5_Cr_8_B_0.05_ alloy. The CTR90-800 condition contains an ultrafine dual-phase heterostructure composed of approximately 80% recrystallized FCC grains and 20% B2 phase, with average grain sizes of about 0.5 and 0.3 μm, respectively [159]. As shown in Figure 19, deformation at a tensile strain of 5% produces pronounced dislocation pile-ups and tangles around FCC/B2 phase boundaries, while pre-existing nanotwins and annealing twins provide additional barriers to dislocation motion. At a higher strain of 15%, dislocation entanglement becomes more extensive in both the FCC and B2 constituents, stacking-fault bundles develop within the FCC matrix, and newly formed deformation twins coexist with the 9R phase [159]. These observations demonstrate that defect strengthening is controlled by the spatial arrangement and sequential evolution of dislocations, twin boundaries, stacking faults, and phase interfaces rather than by the total dislocation density alone.

The above examples mainly represent boundary-, defect-, and interface-controlled strengthening. Their primary contribution is to restrict slip transfer, increase dislocation storage, generate GNDs, and produce back stress through deformation incompatibility. Precipitation-strengthened alloys follow a related but distinct logic. In these alloys, particles can directly strengthen the matrix by impeding dislocation motion, but they can also indirectly modify matrix chemistry, local phase stability, and martensite nucleation behavior. Therefore, the following precipitation examples are discussed by separating the main strengthening mechanism from secondary interactions.

To clarify the primary and secondary strengthening contributions in representative systems, Table 10 summarizes the dominant strengthening mechanism, secondary interactions, and direct or indirect effects for key Fe-based MEAs discussed in this section.

Precipitation is most effective when particle chemistry, spacing, interface character, and matrix stability are co-designed. The primary strengthening mechanism should first be identified as particle strengthening, solute strengthening, interface strengthening, or transformation-assisted hardening, and secondary interactions should then be discussed separately. In Fe-Mn-Al-Ni-C, Kwon et al. [153] used non-shearable Mo-rich M_6_C carbides; the primary contribution comes from M_6_C carbide strengthening and Mo-related solid-solution strengthening. The Mo-added alloy annealed at 800 °C for 7.5 min reached 1274 MPa YS and 1550 MPa UTS, while Mo-rich M_6_C and Mo in solution also contributed secondarily to back-stress hardening [153]. In Co_18.5_Cr_12_Fe_55_Ni_9_Mo_3.5_C_2_, the direct strengthening contribution arises from carbide precipitation and grain refinement, whereas the indirect contribution comes from matrix destabilization. Carbide precipitation lowers ΔG_FCC→BCC_ and accelerates DIMT at high carbide fraction [13]. The A900 and A1000 states combine carbides and fine grains to reach UTS values up to about 1.8 GPa, whereas coarse precipitate-free states exceed 100% elongation [13]. This comparison shows that carbides should not be interpreted only as strengthening particles; they also change the residual matrix chemistry and transformation kinetics. In B-doped Fe_60_Co_15_Ni_15_Cr_10_ [155], the main strengthening mechanism is not direct boride precipitation strengthening. The 1.48% Cr_2_B phase gives only about 7.5 MPa direct precipitation strengthening, whereas Cr depletion from the FCC matrix indirectly destabilizes FCC and raises UTS to about 775 MPa through stronger room-temperature TRIP [155]. This is therefore a matrix-destabilization-assisted TRIP case rather than a conventional boride-strengthened alloy. In W-containing Fe_64_(CoCrNi)_36_ [154], the main strengthening mechanism changes with W partitioning. W atoms first provide solid-solution strengthening when they remain dissolved in the matrix, whereas W-rich precipitates contribute particle strengthening at higher W contents. The 4W alloy reaches 810 MPa YS, 891 MPa UTS, and 26.3% elongation, but the ductile FCC matrix remains essential to arrest microcracks from hard precipitates [154]. This case illustrates the need to balance direct solute/precipitate strengthening with damage tolerance provided by the ductile matrix.

Overall, precipitate effects must be separated into direct and indirect contributions. Direct contributions include particle strengthening, solute strengthening, grain-boundary strengthening, dislocation strengthening, and interface strengthening. Indirect contributions include matrix destabilization, altered SFE or ΔG_FCC→BCC_, redistributed martensite nucleation sites, and improved or degraded damage tolerance. Distinguishing these contributions is essential because the highest-strength condition is not always the condition with the best ductility or service reliability.

### 5.3. TRIP/TWIP-Mediated Plasticity

TRIP directly links stability to hardening. In Fe_45_Co_30_Cr_10_V_10_Ni_5−x_Mn_x_, Ni-to-Mn replacement decreases ΔG_FCC→HCP_ and SFE, reduces the critical strain for transformation, and accelerates martensite formation; the 5Mn alloy forms about 60% BCC martensite at room temperature and approaches 100% BCC+HCP under cryogenic deformation, with tensile strength above 1.6 GPa [6]. Fe_50_Mn_30_Co_10_Cr_10_ shows a similar temperature effect, with HCP fraction rising from about 39% at 298 K to about 79% at 77 K and improved cryogenic strength–ductility balance [54]. At room temperature, grain-size and initial phase constitution further change the phase-specific deformation mechanisms in Fe_50_Mn_30_Co_10_Cr_10_, as shown by the contrast between fine-grained single-FCC A600 and coarser FCC/HCP A900 states [158]. TWIP follows a narrower SFE window. In Fe-25Mn-5Co-12.5Cr-5Ni-2.5Si (in at.%), room-temperature deformation involves planar/wavy dislocations and twins, whereas cryogenic straining adds ε-martensite laths [123]. 

**Figure 20:**
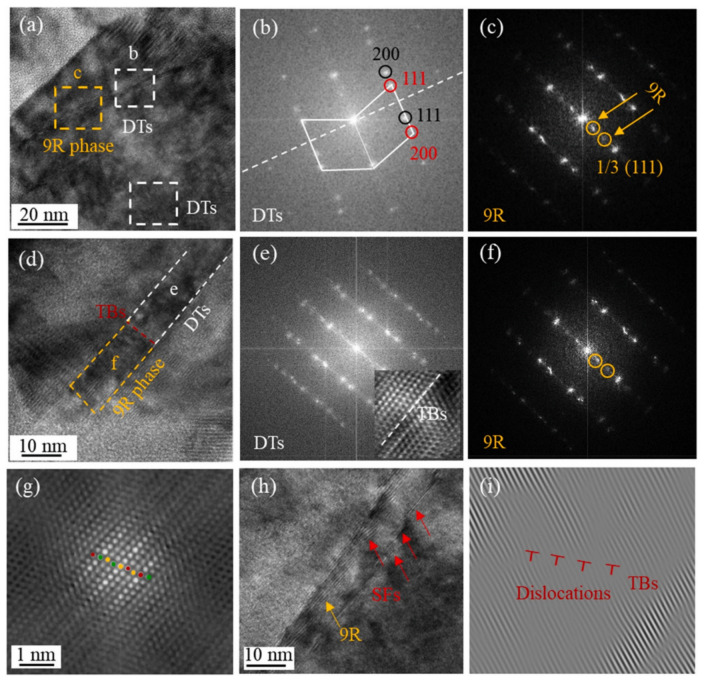
Atomic-scale evidence of deformation-twin-assisted 9R transformation in the cryogenically rolled and annealed Fe_34.95_Ni_28_Co_17.5_Al_11.5_Cr_8_B_0.05_ alloy. (**a**,**d**) High-resolution TEM images showing deformation twins and adjacent 9R transformed regions. (**b**,**c**) FFT patterns of the deformation-twin and 9R regions marked in (**a**), respectively. (**e**,**f**) Corresponding FFT patterns of the deformation-twin and 9R regions marked in (**d**). (**g**) Atomic-resolution image showing the periodic stacking arrangement of the 9R structure. (**h**) Deformation twins containing a 9R region and multiple stacking faults. (**i**) Inverse-FFT image showing dislocation accumulation near the deformation-twin/9R interface. The results demonstrate that deformation twinning provides both additional slip barriers and preferential nucleation sites for subsequent 9R transformation, enabling sequential TWIP–TRIP hardening. Reproduced from Ref. [159].

An atomic-scale illustration of sequential TWIP-TRIP coupling is provided by the cryogenically rolled and annealed Fe_34.95_Ni_28_Co_17.5_Al_11.5_Cr_8_B_0.05_ alloy. As shown in Figure 20, high-resolution TEM and FFT analyses identify deformation-twin regions adjacent to 9R transformed domains. The 9R structure exhibits a periodic stacking sequence associated with stacking-fault ribbons, while dense dislocations accumulate near the deformation-twin/9R interfaces [159]. These observations indicate that deformation twinning first generates additional boundaries and local stress concentration, after which the twin boundaries act as preferred sites for 9R-phase nucleation and propagation. Therefore, TWIP and TRIP operate sequentially rather than independently: newly formed twins dynamically refine the effective slip length and increase defect-storage capacity, whereas subsequent 9R transformation introduces additional phase boundaries and sustains secondary strain hardening [159].

**Figure 21:**
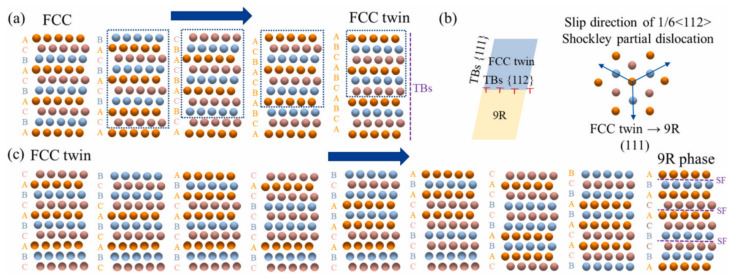
Schematic illustration of the sequential FCC twinning and twin-assisted 9R transformation mechanism in the cryogenically rolled and annealed Fe_34.95_Ni_28_Co_17.5_Al_11.5_Cr_8_B_0.05_ alloy. (**a**) Formation of an FCC deformation twin through the successive glide of 1/6⟨112⟩ Shockley partial dislocations on adjacent (111) planes, converting the original FCC stacking sequence into a mirror-symmetric twinned sequence. (**b**) Schematic representation of a twin-boundary region and the preferential nucleation of the 9R phase through Shockley partial-dislocation activity. (**c**) Transformation of the FCC twin into the long-period 9R structure through the periodic emission of partial dislocations on every third (111) plane. The sequence demonstrates that TWIP-generated twin boundaries provide preferential sites for subsequent 9R transformation, thereby enabling sequential TWIP-TRIP hardening. Reproduced from Ref. [159].

The crystallographic sequence underlying this sequential TWIP-TRIP coupling is summarized schematically in Figure 21. In the cryogenically rolled and annealed Fe_34.95_Ni_28_Co_17.5_Al_11.5_Cr_8_B_0.05_ alloy, the successive glide of 1/6⟨112⟩ Shockley partial dislocations on adjacent (111) planes first converts the original FCC stacking sequence into an FCC twin [159]. With continued deformation, incoherent twin-boundary segments provide preferential sites for the periodic emission of Shockley partial dislocations on every third (111) plane, producing the characteristic long-period 9R stacking sequence [159]. Therefore, deformation twins are not only products of TWIP but also structural templates and local stress-concentration sites for subsequent 9R transformation. The newly generated twin and 9R interfaces further shorten the effective slip length, increase defect-storage capacity, and sustain secondary strain hardening.

This sequential TWIP-TRIP coupling is not restricted to room-temperature deformation. The Fe_46_Mn_35.5_Co_9_Cr_9_N_0.5_ alloy tested at 400 °C provides an instructive elevated-temperature example: although elevated temperature would normally be expected to weaken both mechanisms, this alloy still exhibits sequential activation of TRIP and TWIP together with localized HCP→FCC reversion [160]. This behavior confirms that TRIP and TWIP should not always be treated as independent parallel mechanisms; under suitable local-stability conditions, they can be activated in sequence and sustain multi-stage strain hardening. A composition-controlled version of the same competition appears in Fe_52−x_Mn_27_Cr_15_Co_6_Si_x_. At low Si contents, the alloy remains more TRIP-dominant, and in the 0.3 at.% Si alloy, bct α′-martensite forms at the intersections of bidirectional strain-induced ε-martensite laths. With increasing Si content, the deformation mode shifts toward TWIP as enhanced σ-phase formation modifies the matrix composition and stabilizes fcc-γ [152].

Temperature controls both twinning and transformation. Lower temperatures generally reduce SFE and the barrier to martensitic transformation, so twinning may give way to HCP martensite, and HCP martensite may subsequently facilitate BCC formation, as observed in Fe_45_Co_30_Cr_10_V_10_Ni_5−x_Mn_x_ [6]. Grain refinement often suppresses TRIP, but not universally. In Fe_47_Co_30_Cr_10_Ni_5_V_8−x_Si_x_, a strong TRIP response was preserved after grain refinement in the 6Si alloy because the initial SFE remained in an appropriate window; the same study shows that excessively low SFE can over-accelerate martensitic transformation and reduce ductility [157]. 

**Figure 22:**
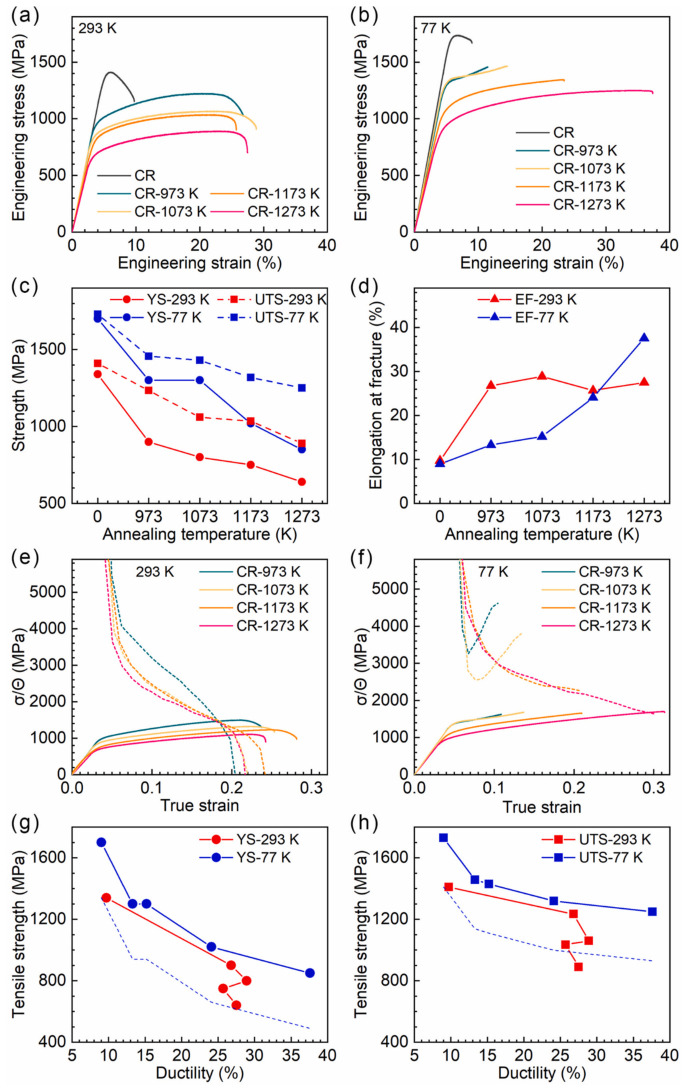
Temperature- and annealing-dependent tensile response of the cold-rolled and annealed Fe_61.5_Cr_17.5_Ni_13_Al_8_ high-entropy alloy. (**a**,**b**) Engineering stress–strain curves of the cold-rolled and annealed conditions tested at 293 and 77 K, respectively. (**c**,**d**) Yield strength, ultimate tensile strength, and elongation at fracture as functions of annealing temperature. (**e**,**f**) True stress-true strain curves and corresponding strain-hardening-rate curves at 293 and 77 K. (**g**,**h**) Relationships between tensile strength and ductility at the two testing temperatures. The low-temperature-annealed conditions exhibit additional FCC→BCC transformation-assisted hardening at 77 K, but their abrupt fracture and limited elongation demonstrate that increased transformation activity does not necessarily produce beneficial cryogenic TRIP when the BCC constituent becomes susceptible to low-temperature brittle failure. Reproduced from Ref. [161].

A further temperature-dependent limitation is demonstrated by the cold-rolled and annealed Fe_61.5_Cr_17.5_Ni_13_Al_8_ alloy. As shown in Figure 22, annealing between 973 and 1273 K produces markedly different strength, ductility, yielding, and strain-hardening responses at 293 and 77 K [161]. At 293 K, the annealed conditions retain elongations of approximately 26–29%, although their strength decreases with increasing annealing temperature. At 77 K, the yield strength similarly decreases as the annealing temperature increases, whereas the elongation rises substantially from 13.3 ± 3.4% in the CR-973 K condition to 37.6 ± 0.9% in the CR-1273 K condition [161]. The CR-973 K and CR-1073 K alloys exhibit a stress plateau followed by an increasing strain-hardening rate at 77 K, indicating an additional hardening contribution from FCC→BCC transformation. Nevertheless, these relatively BCC-rich conditions fracture abruptly before conventional necking, demonstrating that enhanced transformation activity does not necessarily generate a beneficial cryogenic TRIP response.

These results distinguish transformation-induced hardening from transformation-induced ductility. At 77 K, the FCC→BCC transformation in the CR-973 K and CR-1073 K conditions increases the strain-hardening rate, but the transformed and pre-existing BCC constituents undergo a pronounced loss of damage tolerance. Consequently, fracture occurs before the additional hardening can delay necking and extend uniform deformation [161]. Therefore, the mechanical benefit of TRIP depends not only on transformation onset and rate, but also on the intrinsic low-temperature toughness of the product phase, the retained FCC fraction, and the ability of phase interfaces to accommodate strain without initiating cleavage or interfacial cracking.

**Figure 23:**
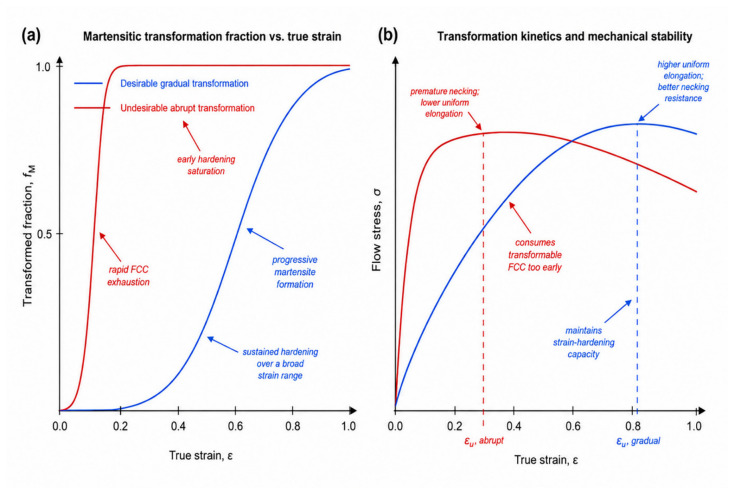
Schematic comparison between desirable gradual transformation and undesirable abrupt transformation in metastable Fe-based MEAs. In the desirable case, martensitic transformation is delayed and proceeds progressively over a broad strain range, continuously introducing new interfaces, sustaining work hardening, and improving uniform elongation and necking resistance. In the undesirable case, martensite forms too early and too rapidly, quickly exhausting the transformable FCC reservoir, reducing subsequent hardening capacity, and promoting premature necking. This schematic emphasizes that transformation kinetics, rather than the maximum final martensite fraction alone, governs the strength–ductility balance.

While the above examples show that SFE, temperature, and grain size regulate TRIP/TWIP activity, the mechanical benefit of transformation-assisted plasticity ultimately depends on transformation kinetics. A desirable transformation mode is not characterized by the fastest martensite formation, but by a delayed and gradual increase in transformed fraction over a broad strain range. This distinction is schematically illustrated in Figure 23.

This schematic also clarifies why TRIP/TWIP-mediated plasticity should be evaluated by transformation onset, transformation rate, and saturation strain rather than by final martensite fraction alone. Gradual transformation maintains a high strain-hardening rate over a longer strain interval, thereby delaying the Considère instability and increasing uniform elongation. In contrast, abrupt transformation may raise strength rapidly at the early stage, but it consumes metastable FCC too soon and causes hardening saturation, local stress concentration, and earlier necking. Therefore, the optimal TRIP/TWIP design is a kinetic balance: transformation should be sufficiently activated to sustain work hardening, but sufficiently delayed and distributed to preserve deformation compatibility and necking resistance.

### 5.4. Strain-Rate Response

As a background multiphase HEA case rather than a representative Fe-based MEA, Al_0.3_CoCrFeNiTi_0.3_ is discussed here only to illustrate general dynamic constitutive behavior under impact loading. In this related system, split Hopkinson pressure bar (SHPB) and Taylor impact tests reveal strain-rate hardening, thermal softening, and deformation involving dislocations, kink bands, stacking faults, Lomer–Cottrell locks, and nanotwins, and a modified Johnson–Cook–Cowper–Symonds model was used to describe dynamic plastic flow [162]. Therefore, the strain-rate response of Fe-based MEAs should be interpreted through coupled transformation kinetics, twinning activity, adiabatic heating, reverse transformation, and damage evolution, rather than through strain-rate hardening alone.

As summarized in recent dynamic-compression work on Fe_50_Mn_30_Co_10_Cr_10_, previous high-speed tensile and SHPB studies reported non-monotonic strength and uniform elongation with strain rate, a transition from martensitic transformation to twinning during dynamic deformation, and adiabatic-heating-induced HCP→FCC reverse transformation [163]. 

Taylor-impact tests on Fe_50_Mn_30_Co_10_Cr_10_ show that local strain gradients promote FCC→HCP transformation, higher boundary density, and, at high velocity, HCP→FCC reverse transformation with FCC twinning because of adiabatic heating [164]. 

Recent studies clarify how metastability can be used without sacrificing plastic stability. In pre-deformed Fe_60_Co_15_Ni_15_Cr_10_ MEAs, high dislocation density from hot rolling initially promotes strain localization, but localized deformation-induced martensitic transformation increases strain-rate sensitivity, redistributes strain, and enables strain delocalization [165]. Dynamic tensile tests on Fe_40_Mn_40_Co_10_Cr_10_ and its C-interstitial variant show a clear strain-rate effect. At high strain rates, the alloys exhibit increased strength but reduced uniform elongation. This reduction results from adiabatic temperature rise, which increases the stacking-fault energy, suppresses twinning, and lowers the strain-hardening rate. In contrast, C addition enhances twinning capability and improves resistance to shear-band formation [166]. Together, these findings show that the strain-rate response of Fe-based MEAs is governed by the coupling of metastability, adiabatic heating, dislocation slip, twinning, martensitic transformation, reverse transformation, and strain localization.

### 5.5. Service-Oriented Performance

Service-oriented performance should be evaluated across both property categories and service environments. For Fe-based MEAs, tensile strength and ductility represent only the first screening level. Engineering qualification further requires fatigue resistance, fracture toughness, impact toughness, corrosion and corrosion-fatigue resistance, hydrogen tolerance, creep and stress relaxation, weldability, formability, irradiation tolerance, and long-term microstructural stability. These properties should be separated according to room-temperature, cryogenic, elevated-temperature, and corrosive or hydrogen-containing environments, because the same metastable microstructure may be beneficial in one environment but detrimental in another. Therefore, this section discusses service performance by distinguishing well-studied properties from underreported or missing validation data. Fe_50_Co_25_Ni_10_Al_5_Ti_5_Mo_5_ represents an ultrahigh-strength cryogenic route based on retained FCC, thermally induced BCC martensite, high dislocation density, and Mo-rich μ precipitates [25]. Its low-temperature response further illustrates how deformation-induced BCC martensite and phase-stress partitioning contribute to strain hardening and load bearing. 

**Figure 24:**
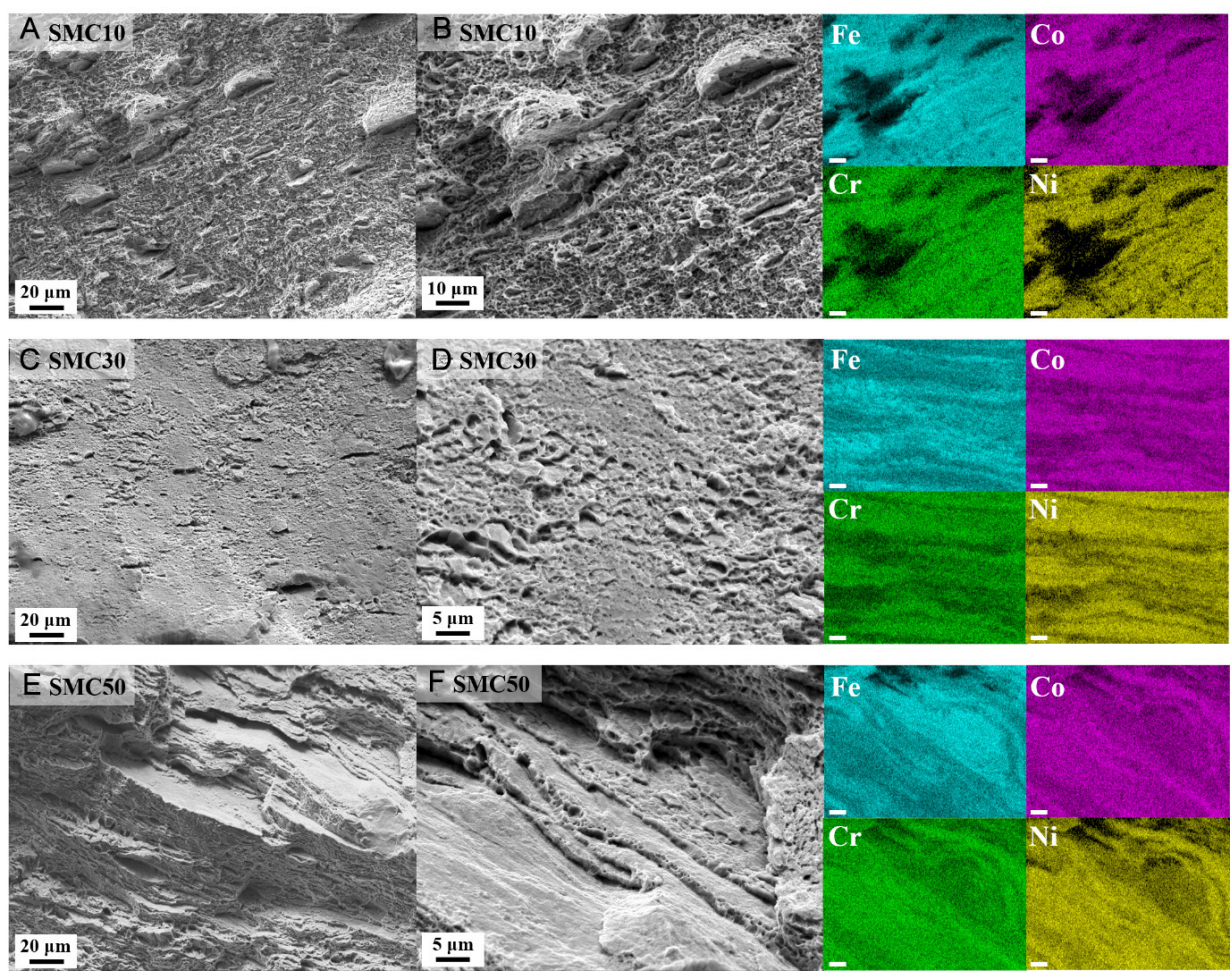
Cryogenic fracture behavior and interfacial damage characteristics of spatial-metastability-control ferrous medium-entropy-alloy/Fe heterostructures. (**A**,**C**,**E**) Low-magnification fracture-surface images of the SMC10, SMC30, and SMC50 conditions after tensile testing at 77 K, respectively. (**B**,**D**,**F**) Corresponding high-magnification fracture morphologies and Fe, Co, Cr, and Ni elemental maps. SMC10 and SMC30 exhibit predominantly ductile fracture characteristics, whereas SMC50 shows strip-like interfacial peeling and layered delamination. The comparison demonstrates that cryogenic service reliability depends not only on strength and transformation activity, but also on compositional balance, interfacial cohesion, and resistance to damage localization. Reproduced from Ref. [42].

A more service-oriented criterion is whether heterogeneous interfaces remain mechanically stable under cryogenic loading. A direct example is provided by the spatial-metastability-control (SMC) materials produced by integrating Fe-rich regions into a metastable Fe_60_Co_15_Cr_10_Ni_15_ ferrous medium-entropy alloy. The resulting architecture contains MEA regions, Fe-rich regions, and compositionally graded transition regions with different phase stabilities and local strengths [42]. As shown in Figure 24, the SMC10 and SMC30 conditions exhibit predominantly ductile fracture characteristics after tensile testing at 77 K. In contrast, SMC50 shows strip-like interfacial peeling and layered delamination, indicating that excessive compositional and phase contrast can reduce cryogenic interfacial damage tolerance [42]. The corresponding Fe, Co, Cr, and Ni elemental maps further associate the fracture morphology with the chemically heterogeneous constituent regions. Notably, SMC30 reaches a yield strength of approximately 1107.7 MPa and an ultimate tensile strength of approximately 1423.3 MPa at 77 K while avoiding the pronounced interfacial delamination observed in SMC50 [42].

These observations demonstrate that high cryogenic tensile strength and extensive TRIP activity are insufficient as stand-alone indicators of service readiness. In compositionally heterogeneous alloys, the integrity of transition regions and hard–soft interfaces determines whether local strain partitioning produces beneficial hardening or evolves into interfacial separation. Therefore, cryogenic alloy qualification should include fracture morphology, interfacial cohesion, crack-initiation location, and damage-propagation behavior in addition to conventional yield strength, ultimate tensile strength, and elongation.

Service stability also depends on non-mechanical environments. In FeCrMnNiC_x_ alloys, low-carbon FeCrMnNiC_0.1_ was reported as a single-phase FCC alloy, higher carbon contents produced two-phase microstructures, the equiatomic alloy showed the best corrosion resistance in 3.5% NaCl solution, and FeCrMnNiC_0.1_ retained phase and chemical stability after 600 keV Xe-ion irradiation to 50 dpa at 623 K [167]. Because tensile properties alone cannot determine engineering applicability, representative service-related properties and qualification implications of Fe-based MEAs are summarized in Table 11. These examples include cryogenic tensile/Charpy response, fatigue after surface treatment, low-cycle fatigue, cryogenic tension-corrosion coupling, corrosion behavior, welding response, and wide-temperature strength–ductility retention [7,12,36,37,38,168,169,170,171].

Although Table 11 lists representative service-related examples, a broader checklist is needed to identify the maturity of service–property validation across alloy families. Table 12 summarizes whether key service properties are relatively well studied, underreported, or largely missing for major Fe-based MEA families under room-temperature, cryogenic, elevated-temperature, and corrosive or hydrogen-containing environments.

Beyond single-temperature tensile properties, wide-temperature property retention is also important for service-oriented design. The L1_2_-strengthened Fe_49.6_Ni_28_Al_6_Ti_3_Zr_0.1_C_0.2_B_0.1_Cr_13_ medium-entropy alloy provides a representative route for retaining strength–ductility performance from cryogenic to elevated temperatures [38]. However, Table 12 shows that wide-temperature tensile retention is only one part of service qualification. For structural applications, the same alloy family should also be evaluated for fatigue resistance, fracture toughness, creep or stress relaxation, corrosion-fatigue, hydrogen tolerance, and long-term precipitate stability.

**Figure 25:**
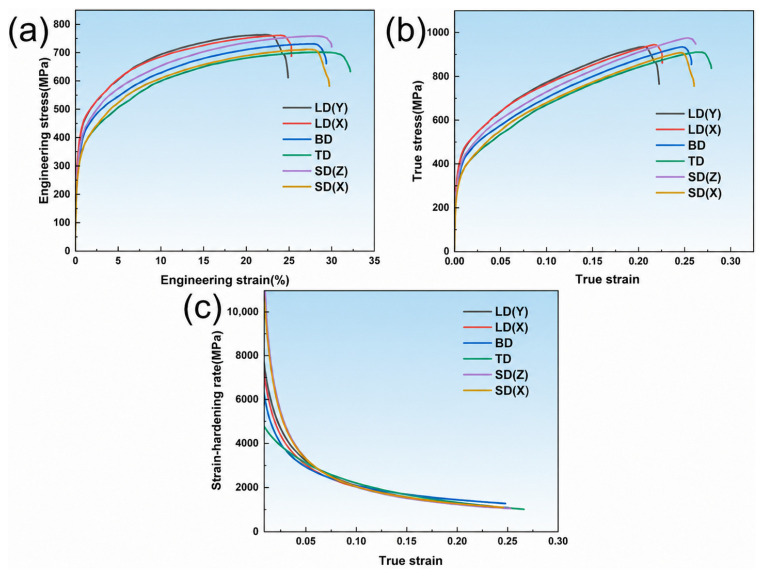
Direction-dependent tensile behavior of the laser-metal-deposited Fe_60_Mn_27_Co_3_Cr_10_ dual-phase high-entropy alloy. (**a**) Engineering stress–strain curves, (**b**) true stress–strain curves, and (**c**) strain-hardening-rate curves for specimens extracted along different directions and planes of the deposited block. LD(x) and LD(y) represent the two laser-deposition directions within the deposition plane, BD denotes the build direction, TD denotes the transverse direction, and SD(x) and SD(z) denote the secondary directions along the corresponding axes. The deposition-direction specimens exhibit higher yield and ultimate tensile strengths but lower elongations, whereas the other orientations show reduced strength and comparatively higher ductility. The results highlight the need to evaluate build-direction-dependent strength, ductility, and mechanical-property consistency when qualifying additively manufactured Fe-based medium-entropy alloys. Reproduced from Ref. [76].

Manufacturing-route-dependent anisotropy provides another important service-qualification criterion. In the laser-metal-deposited Fe_60_Mn_27_Co_3_Cr_10_ alloy, tensile properties vary systematically with the sampling plane and loading direction, as shown in Figure 25. Specimens loaded along the two laser-deposition directions, LD(x) and LD(y), exhibit the highest yield strengths of approximately 375 and 369 MPa and ultimate tensile strengths of approximately 761 and 763 MPa, respectively, but their elongations are limited to about 25% [76]. In comparison, specimens loaded along the build, transverse, and secondary-build directions show lower yield strengths of approximately 296–332 MPa but higher elongations of approximately 29.3–31.9% [76]. Despite these directional differences in strength and ductility, the six orientations exhibit broadly similar strain-hardening-rate evolution. These results demonstrate that service qualification of additively manufactured Fe-based medium-entropy alloys must consider not only nominal composition and average tensile properties, but also build orientation, sampling direction, microstructural anisotropy, and property reproducibility.

This directional response illustrates that a mechanically attractive alloy composition does not automatically guarantee component-level isotropy. In additively manufactured materials, solidification direction, columnar-grain morphology, phase morphology, and interlayer thermal history can produce location- and orientation-dependent yielding even when porosity and global phase constitution are similar. Accordingly, qualification should include specimens extracted from multiple build planes and loading directions, together with statistical evaluation of defect sensitivity, fatigue behavior, residual stress, and local fracture initiation.

Cyclic stability, forming reliability, weldability, wear resistance, and dimensional stability probe deformation compatibility from different directions. In Fe_40_Mn_40_Co_10_Cr_10_, Shams et al. [37] found slip-dominated cyclic deformation and high-amplitude twinning; UNSM raised YS but worsened fatigue resistance [37]. In Fe_50−x_Mn_30_Co_10_Cr_10_Si_x_, Shams et al. [168] showed that up to 4 at.% Si changes monotonic tension only slightly but strongly affects cycling by promoting reversible γ ↔ ε transformation, stress saturation, or secondary hardening, with fatigue lives above 10,000 cycles at 0.7% strain amplitude [168]. Choi et al. [40] confirmed that DIMT stabilizes or destabilizes deformation depending on loading mode. Low-thermal-expansion Co_22.2_Cr_6.2_Fe_48.8_Ni_17.8_Cu_5.0_ highlights cryogenic dimensional stability [173]. Cu-rich weld-metal phase separation adds a welding-related strengthening route [171,174]; in gas tungsten arc (GTA) welds of Fe_60_Co_15_Ni_15_Cr_10_, CrFeMnNiCu filler produces FCC_1_+Cu-rich FCC_2_, higher weld-metal hardness/strength, and cryogenic base metal/heat-affected zone (BM/HAZ) DIMT plus weld-metal twinning [171]. Wear and edge formability also require separate assessment [114,175], and low-cycle fatigue in Fe_50_Mn_30_Co_10_Cr_10_ shifts from FCC→HCP transformation at low strain amplitudes to HCP twinning and partial reverse transformation at high amplitudes [172].

Corrosion confirms that service readiness cannot be inferred from strength. Wei et al. [12] showed that Fe_49.5_Mn_25_Cr_15_Ni_10_C_0.5_ has the best 77 K strength-ductility product among the compared alloys but only intermediate corrosion resistance; Fe_74.5_Cr_15_Ni_10_C_0.5_ performs best electrochemically and Fe_74.5_Mn_25_C_0.5_ worst [12]. Co-for-Ni substitution in Fe_50_Mn_25_Cr_15_Ni_10−x_Co_x_ improves room-temperature strength but reduces cryogenic ductility and corrosion resistance as HCP fraction increases [7]. Dual-phase Fe_61.5_Cr_17.5_Ni_13_Al_8_ resists NaCl and H_2_SO_4_ through Fe/Cr oxide-hydroxide passive films [169]. Small Cu additions to FeMnCoCr improve strength-ductility and NaCl corrosion resistance by stabilizing the matrix and compacting the passive film [170]. In Fe_68_Ni_10_Mn_10_Co_10_Ti_1.5_Si_0.5_, the austenitic state shows the best corrosion resistance because it forms a dense TiO_2_/SiO_2_-containing layer [39]; selectively corroded dual-phase structures are less protective [39]. 

For service deployment, tensile strength must be considered together with crack tolerance, cyclic stability, passive-film durability, formability, welding response, processing sensitivity, and cost. These requirements have been separately addressed in studies on impact toughness and damage tolerance [36,40], fatigue response [168,172], corrosion/passivation behavior [167,169,170,176], stretch-flangeability [175], weldability [171,174], and processing-sensitive microstructural stability [63,140,143,148]. The useful design window is therefore defined not by peak strength, but by whether the same structure can resist localization, fracture, fatigue, corrosion, hydrogen-assisted damage, creep, and long-term microstructural degradation. At present, most Fe-based MEA studies provide relatively rich room-temperature and cryogenic tensile datasets, whereas fracture toughness, fatigue-crack growth, corrosion-fatigue, hydrogen embrittlement, creep, weld-joint efficiency, and long-term aging remain much less systematic. Future work should therefore move from isolated tensile-property optimization toward service-property matrices that evaluate alloy families under realistic thermal, mechanical, chemical, and joining conditions.

## 6. Current Challenges

Fe-based MEAs have progressed from equiatomic model alloys toward Fe-rich and low-cost compositions, but their transition from promising tensile materials to validated structural alloys remains incomplete. The key challenge is not the absence of strengthening or transformation mechanisms, but the lack of integrated evidence linking cost, metastability control, processing reproducibility, mechanism separation, and service reliability. To avoid repeating individual examples already discussed in Section 3, Section 4 and Section 5, the main challenges are summarized in Table 13 as a numbered checklist, together with the corresponding evidence gaps and recommended validation approaches.

Taken together, these challenges show that future Fe-based MEA research should move from isolated tensile-property optimization toward integrated qualification. The most promising alloys are not simply those with the highest strength, largest elongation, or lowest nominal cost, but those whose metastability, strengthening mechanisms, processing response, and service properties remain stable across realistic thermal, mechanical, chemical, and joining conditions. Therefore, Fe-based MEAs should be benchmarked against steels, Ni-based alloys, and conventional cryogenic alloys using the same service-property and manufacturing criteria.

## 7. Future Perspectives

Future development of Fe-based MEAs should move from mechanism discovery toward staged, service-oriented qualification. The immediate priority is to make existing data comparable, reproducible, and useful for alloy down-selection. The mid-term priority is to connect composition, processing, microstructure, deformation mechanisms, and service properties through standardized benchmark systems. The long-term priority is to establish validated predictive models and certifiable alloy families that can compete with steels, Ni-based alloys, and conventional cryogenic alloys. Therefore, the recommendations are arranged in Table 14 according to near-term, mid-term, and long-term deliverables.

This staged roadmap emphasizes that future Fe-based MEA research should produce reusable outputs rather than isolated property records. In the near term, the most valuable deliverables are open datasets, benchmark alloy families, and standard processing histories. In the medium term, the field should establish property matrices and mechanism maps that connect metastability, microstructure, processing, and service performance. In the long term, validated constitutive models and certifiable alloy families are required for engineering adoption. Such a staged strategy can convert Fe-based MEAs from promising laboratory alloys into reproducible structural materials.

## 8. Conclusions

1. Fe-based MEAs should be regarded as metastability-engineered structural alloys rather than entropy-stabilized solid solutions alone. Their central design principle is to place the FCC matrix near controlled HCP or BCC transformation boundaries, enabling transformation, twinning, solute strengthening, precipitation, and heterostructure hardening to act cooperatively.
2. Composition determines the thermodynamic and chemical basis of metastability by regulating FCC stability, stacking-fault energy, lattice friction, precipitation tendency, and elemental partitioning. However, nominal composition alone cannot predict performance, because alloying effects depend strongly on local chemistry, concentration, and processing history.
3. Microstructure controls how chemical metastability is activated during deformation. Grain size, recrystallized fraction, retained defects, precipitates, phase morphology, and compositional heterogeneity regulate martensite nucleation, twin formation, strain partitioning, and deformation uniformity.
4. Strengthening in Fe-based MEAs is coupled rather than simply additive. A favorable strength–ductility balance requires solute strengthening, grain refinement, dislocation storage, precipitation, TRIP/TWIP activity, and HDI hardening to be synchronized so that work hardening is sustained and localization is delayed.
5. Temperature, strain rate, and loading path must be treated as design variables. Cryogenic, room-temperature, elevated-temperature, and dynamic conditions can change the balance among slip, twinning, martensitic transformation, recovery, and damage accumulation; therefore, quasi-static tensile properties alone are insufficient for evaluating alloy reliability.
6. Processing and service conditions determine whether a promising metastable alloy can become an engineering material. Rolling, annealing, surface treatment, additive manufacturing, and welding can modify phase stability, defect density, segregation, residual stress, and local deformation behavior, while fatigue, fracture toughness, creep, corrosion, hydrogen tolerance, weldability, and long-term stability remain essential validation criteria.

Therefore, the development of Fe-based MEAs must shift from tensile-property optimization toward the deployment of validated structural alloys with reproducible processing routes, predictable deformation mechanisms, and verified service reliability.

## Figures and Tables

**Table 1:** Conceptual map linking SFE regime, phase stability, deformation mode, and failure risk in Fe-based MEAs [3,4,5,8,9,10,13,28,29,30,40].

SFE Regime	Phase-Stability Indicators	Dominant Deformation Mode	Beneficial Condition	Potential Failure Risk
Very low SFE/highly unstable FCC	Large driving force for FCC→HCP or FCC→BCC transformation; low FCC stability; favorable ΔG for martensite formation	Rapid TRIP or DIMT; early HCP/BCC martensite formation	Useful only when transformation is delayed and spatially distributed	Sudden transformation; rapid exhaustion of transformable FCC; strain localization; premature necking or fracture
Low-to-moderate SFE/controlled metastability	FCC remains transformable but not immediately unstable; suitable SFE/ΔG/VEC window	Progressive TRIP, TWIP-assisted plasticity, or combined TRIP/TWIP	Most favorable regime for sustained strain hardening and strength–ductility synergy	If local instability is excessive, transformation may concentrate around shear bands, defects, or segregated regions
Moderate SFE/relatively stable FCC	Reduced martensitic driving force; FCC stability retained over a larger strain range	TWIP, planar slip, or mixed slip-twinning deformation	Good ductility and delayed localization when strain hardening is sufficient	Limited TRIP/DIMT contribution; insufficient transformation strengthening
High SFE/over-stable FCC	High FCC stability; weak thermodynamic tendency for HCP/BCC transformation	Dislocation slip-dominated deformation	Useful for ductility or formability when high transformation hardening is not required	Low strain-hardening capacity and early plastic instability under high-strength design requirements
Locally unstable microstructure despite suitable nominal SFE	Nominal SFE may be acceptable, but grain size, defects, shear bands, precipitates, local segregation, or AM cell boundaries create local transformation sites	Spatially heterogeneous TRIP/DIMT and strain partitioning	Beneficial when local transformation is well distributed	Local hardening mismatch; microcrack initiation; damage accumulation; premature failure

Note: The SFE regimes are qualitative and system-dependent because the effective deformation mode also depends on ΔG, VEC, temperature, strain state, grain size, defect density, precipitates, and local chemical heterogeneity. The conceptual relationships summarized in this table were compiled from studies on metastability engineering, deformation-induced martensitic transformation, TRIP/TWIP behavior, transformation kinetics, and microstructure-regulated local metastability in Fe-based MEAs and related metastable HEAs [3,4,5,8,9,10,13,28,29,30,40].

**Table 2:** Matrix- and processing-dependent effects of representative alloying elements in Fe-based MEAs, with representative references.

Element	Primary and Secondary Effects	Typical Benefit	Main Risk
**Fe** [1,5,40,46]	Primary: low-cost base element for Fe-rich non-equiatomic design. Secondary: higher Fe can lower FCC stability and promote FCC→BCC transformation depending on Co/Ni/Cr balance.	Cost reduction; TRIP/DIMT-assisted cryogenic hardening.	Excessive FCC destabilization, rapid martensite formation, and ductility loss.
**Mn** [4,6,7,8,11,47]	Primary: lowers SFE and favors FCC→HCP transformation. Secondary: effect depends on Ni/Co balance, grain size, temperature, and initial HCP fraction.	TWIP/TRIP synergy and sustained cryogenic work hardening.	Excess HCP or rapid transformation can exhaust transformable FCC.
**Ni** [6,7,11,12]	Primary: stabilizes FCC. Secondary: counterbalances Mn/Co-driven destabilization and helps retain transformable FCC.	Ductility, processability, and stability-window control.	Over-stabilized FCC may limit TRIP/DIMT strengthening.
**Co** [7]	Primary: can lower SFE relative to Ni in Fe-Mn-Cr-Ni/Co substitution systems and shift FCC toward FCC+HCP or HCP-rich states. Secondary: Fe-Mn-Co-Cr systems can exhibit composition- and temperature-dependent multi-step TRIP/TWIP behavior [47].	SFE tuning and TWIP/TRIP balance.	Excess HCP-rich initial constitution can reduce transformable FCC and ductility.
**Cr** [7,12,18,38,39]	Primary: supports Fe-Cr-Ni/Co matrix design and passivation. Secondary: may participate in carbides or passive-film chemistry.	Corrosion resistance and matrix/solid-solution stabilization.	Cr-rich carbides or unfavorable phase separation may reduce strain compatibility.
**Mo** [13,14,15,18,45]	Primary: strong solute strengthening and μ/carbide precipitation. Secondary: partitioning/carbides modify residual FCC stability and DIMT kinetics.	High strength; precipitation/TRIP coupling; lattice resistance.	Coarse/excess precipitates may reduce ductility and processability.
**Al** [16,43]	Primary: promotes FCC→BCC or FCC + BCC transformation by reducing VEC and increasing lattice distortion in suitable matrices. Secondary: minor Al addition can also contribute to solid-solution and lattice-strain strengthening depending on matrix chemistry and processing [24].	BCC/B2 strengthening, density reduction, and precipitation-assisted hardening.	Excess BCC/B2 or brittle intermetallics can reduce ductility and toughness.
**Ti** [25,27,32,35]	Primary: participates in Ti-containing precipitates, μ-phase-related strengthening, or TiC-related phase-stability tuning depending on alloy design and processing. Secondary: C-Ti microalloying combined with cryogenic rolling/annealing can introduce deformation twins, dislocations, microbands, and nanoscale M_23_C_6_ carbides [26].	Precipitation strengthening and stability tuning.	Austenite overstabilization, brittle particles, or coarse precipitates may weaken transformation hardening.
**Si** [9,17]	Primary: reduces Co/Ni demand and promotes FCC + BCC, Lüders deformation, DIMT, and HDI hardening. Secondary: depends on Fe-rich matrix, Al co-addition, grain refinement, and processing.	Low-cost strengthening; TRIP/HDI-assisted hardening.	Excess BCC fraction or localized deformation may impair ductility/toughness.
**C** [12,18,22,23,24,45,46]	Primary: interstitial strengthening, FCC stabilization, or carbide formation. Secondary: can support TWIP-dominant FCC deformation or TRIP/carbide coupling depending on matrix chemistry and processing.	Interstitial/carbide strengthening and TWIP/TRIP-assisted hardening.	Excess carbides, brittle fracture, or loss of strain compatibility.
**B** [21,48,49,50,51]	Primary: trace B can provide solid-solution, grain-boundary, or interface-strengthening effects without necessarily forming borides [21,48] Secondary: higher B contents can promote M_2_B-type borides, increase hardness or wear resistance, and may raise SFE or suppress transformation-assisted plasticity [49,50,51].	Boundary/interface strengthening and sequential deformation compatibility.	Excess B or borides may embrittle boundaries.
**N** [19,20,52,53]	Primary: interstitial strengthening and local chemical-order effects. Secondary: can form nitrides or remain supersaturated; may suppress FCC→HCP TRIP and shift deformation toward TWIP.	Solid-solution/LCO strengthening; TWIP/HDI-assisted hardening.	Nitride embrittlement, suppressed beneficial TRIP, or local instability.

Note: The listed effects are qualitative tendencies rather than universal rules. The actual role of each element depends on the base matrix, phase constitution, SFE/ΔG/VEC, temperature, processing route, segregation behavior, and precipitation state. Representative references supporting the elemental effects are provided after each element. FCC, face-centered cubic; BCC, body-centered cubic; HCP, hexagonal close-packed; TRIP, transformation-induced plasticity; TWIP, twinning-induced plasticity; DIMT, deformation-induced martensitic transformation; HDI, hetero-deformation-induced strengthening; DED, directed energy deposition; LCO, local chemical order.

**Table 3:** Classification and comparison of representative Fe-based MEA families, with representative references.

Alloy Family	Design Objective and Phase-stability Route	Typical Processing and Representative Property Range	Unresolved Limitations
Fe-Co-Ni-Cr-based systems [5,28,30,40]	Develop Fe-rich metastable FCC matrices for transformation-assisted strength–ductility synergy, especially at cryogenic temperature. The Fe/(Co, Ni, Cr) balance regulates FCC stability; increasing Fe generally destabilizes FCC and can activate FCC→BCC deformation-induced martensitic transformation (DIMT).	Casting, homogenization, cold/hot rolling, annealing, partial recrystallization, additive manufacturing, and post-processing heat treatment. Representative Fe-rich systems can reach about 1.5–1.7 GPa tensile strength with large ductility at 77 K when FCC→BCC transformation is gradual and distributed.	Excessive FCC destabilization may cause rapid martensite formation, early necking, and strong processing sensitivity. Quantitative links among composition, local heterogeneity, and DIMT kinetics remain insufficient.
Fe-Mn-Cr-Ni/Co-based systems [7,8,11,47]	Tune low-SFE FCC matrices for balanced slip, TWIP, and FCC→HCP TRIP while reducing dependence on high Co or Ni contents. Mn, Ni, and Co jointly control SFE, FCC→HCP driving force, and initial FCC/HCP phase constitution; Ni stabilizes FCC, whereas Mn and Co can promote low-SFE deformation pathways.	Casting, homogenization, cold rolling, cryorolling, annealing, grain refinement, cryogenic deformation, and heterostructure processing. Many alloys show tensile strengths around or above 1 GPa at 77 K while retaining high ductility when TWIP and TRIP are progressively activated.	Excess initial HCP phase or rapid FCC→HCP transformation may exhaust transformable FCC and reduce ductility. Criteria distinguishing beneficial TWIP/TRIP synergy from premature localization are still needed.
Fe-Co-Cr-Ni-Mo-based systems [13,14,15,65]	Combine substitutional solid-solution strengthening, precipitation strengthening, and metastability control in Fe-rich matrices. Mo partitioning, μ-phase or carbide precipitation, and matrix-composition changes modify FCC stability and FCC→BCC DIMT kinetics.	Casting, cold rolling, annealing, high-pressure torsion, ultrasonic nanocrystal surface modification, laser powder bed fusion, directed energy deposition, and post-processing heat treatment. Strength can approach or exceed 1–2 GPa at low temperature when Mo-related strengthening and transformation-assisted hardening are coupled.	Coarse precipitates, excessive μ phase or carbide formation, and matrix depletion may reduce ductility, processability, and long-term stability. Precipitation, partitioning, and DIMT contributions remain difficult to separate quantitatively.
Al/Ti/Si-modified systems [16,17,25,26,27,32]	Expand the design space through dual-phase design, ordered precipitation, cost reduction, and controlled metastability. Al and Si tend to promote FCC + BCC/B2 constitution by reducing VEC or increasing lattice distortion, whereas Ti promotes L1_2_, B2, μ-phase, or TiC-related precipitation and alters FCC stability.	Casting, rolling, annealing, high-pressure torsion, precipitation heat treatment, directed energy deposition, additive manufacturing, and thermomechanical processing. High strength with useful ductility can be obtained when FCC/BCC balance, nanoscale precipitation, and transformation kinetics are controlled.	Excess BCC/B2 formation, brittle intermetallics, or coarse TiC-related particles may impair ductility and toughness. TiC decomposition or austenite overstabilization may suppress beneficial DIMT in some cases.
C/B/N-interstitially modified systems [18,19,20,21,46,52,53]	Introduce interstitial strengthening, local chemical-order effects, carbide/nitride precipitation, and boundary/interface regulation. C, B, and N alter SFE, lattice friction, local chemical order, precipitation behavior, and interface stability in a strongly matrix- and processing-dependent manner.	Casting, rolling, annealing, thermomechanical treatment, surface alloying, nitriding-related processing, coating preparation, and post-processing heat treatment. Some C- or N-modified alloys show strength around or above 1 GPa with large ductility when interstitial strengthening, precipitation, and metastability are balanced.	Excess carbide or nitride precipitation, grain-boundary embrittlement, and suppressed beneficial TRIP may reduce deformation compatibility. Contributions from interstitial strengthening, precipitation, local chemical order, and metastability remain insufficiently deconvoluted.

Note: FCC, face-centered cubic; BCC, body-centered cubic; HCP, hexagonal close-packed; SFE, stacking-fault energy; VEC, valence electron concentration; TRIP, transformation-induced plasticity; TWIP, twinning-induced plasticity; DIMT, deformation-induced martensitic transformation. The listed property ranges are representative values rather than fixed limits, because measured properties depend on composition, phase fraction, grain size, processing history, testing temperature, and loading condition.

**Table 4:** Effect of Fe content on phase constitution and deformation behavior in Fe-Co-Ni-Cr-based MEAs, with representative core references.

Fe Level/Representative Systems	Phase Constitution or Stability State	Dominant Deformation Behavior	Mechanical Implication
Moderate Fe content, such as Fe60-type alloys in Fe-rich Fe-Co-Ni-Cr or Fe-Co-Cr-Mn-Ni systems [5,67]	Mainly FCC or metastable FCC with retained transformable austenite	Slip/TWIP-dominated deformation or delayed FCC→BCC DIMT, depending on temperature and local microstructure	Good ductility can be retained, but transformation-assisted strengthening is limited when FCC is too stable
Intermediate-to-high Fe content, such as Fe_60_Co_15_Ni_15_Cr_10_ and Fe_65_Co_10_Ni_10_Cr_15_ [5,10,68]	Metastable FCC or heterogeneous FCC/BCC structure	Progressive FCC→BCC DIMT, phase-stress partitioning, and sustained strain hardening during cryogenic deformation	Beneficial strength–ductility synergy when martensite formation is gradual, spatially distributed, and accompanied by load transfer from FCC to BCC
Higher Fe content, such as Fe70-type alloys in Fe_x_(CoCrMnNi)_100−x_ or less stable Fe_64_(CoNi)_26_Cr_10_ [40,67]	Lower FCC stability, larger BCC fraction, or stronger tendency for rapid BCC martensite formation	Stronger TRIP/DIMT, but transformation can occur too early or too locally under uniaxial tension	Higher strength may be obtained at the expense of uniform elongation; excessive or localized DIMT can accelerate strain localization and lead to quicker necking
Excessively Fe-rich compositions in composition-gradient Fe_x_(CoCrNi)_100−x_ alloys [66]	Phase constitution shifts from FCC/FCC + BCC toward BCC-dominated structures	Reduced availability of transformable FCC; deformation increasingly controlled by BCC-containing structures	Transformation hardening becomes less effective once the FCC stability window is lost, and ductility or toughness may decrease

Note: FCC, face-centered cubic; BCC, body-centered cubic; TWIP, twinning-induced plasticity; TRIP, transformation-induced plasticity; DIMT, deformation-induced martensitic transformation. The Fe levels are summarized qualitatively because the exact phase boundary depends on Co/Ni/Cr balance, grain size, processing route, temperature, and local chemical heterogeneity. Core references directly supporting each Fe-content regime are provided after the representative systems.

**Table 5:** Mo-specific strengthening and metastability-control mechanisms in Fe-Co-Cr-Ni-Mo-based MEAs, with core references.

Mo-Related Route	Main Strengthening/Stability-Control Role	Ductility-Loss Location or Risk
Solid-solution strengthening [65,81]	Mo dissolved in the FCC matrix increases lattice distortion and lattice-friction stress, thereby raising yield strength while retaining FCC plasticity when precipitation and destabilization are limited.	Ductility loss is usually limited; risk increases only when high lattice resistance is not balanced by sufficient work hardening or strain compatibility.
μ-phase precipitation [14]	Fine intragranular μ-phase precipitates can strengthen the FCC matrix and interact with stored defects; longer annealing may produce coarser intergranular precipitates.	Ductility loss is most likely at coarse or intergranular μ-phase regions, which act as strain-localization and damage-initiation sites.
Carbide formation [13,18,84]	Cr-rich M_23_C_6_, Mo-rich M_6_C, MC-type carbides, or related carbides strengthen the matrix and can alter residual matrix chemistry, martensite nucleation sites, and DIMT kinetics.	Ductility decreases around excessive or coarse carbide-rich regions because particle/matrix decohesion, microvoid formation, and localized deformation become easier.
Partitioning-induced FCC destabilization [14,18]	Mo partitioning into precipitates chemically modifies or depletes the surrounding FCC matrix, reducing FCC stability and promoting FCC→BCC DIMT during deformation.	Beneficial only when DIMT is gradual and distributed; excessive matrix destabilization causes rapid/localized DIMT, early exhaustion of transformable FCC, and premature necking.
Processing sensitivity [14,88,90]	Cold rolling/annealing, LPBF-related cell-network restructuring, and laser surface treatment control defect density, grain size, precipitate distribution, cell structures, and transformation kinetics.	A direct ductility-loss case is laser-treated Co_17.5_Cr_12.5_Fe_55_Ni_10_Mo_5_, where single laser scanning increased YS but reduced UE from about 40% to about 8%; more broadly, overaged, over-strengthened, or locally destabilized states are risky.

Note: FCC, face-centered cubic; BCC, body-centered cubic; DIMT, deformation-induced martensitic transformation; HPT, high-pressure torsion; UNSM, ultrasonic nanocrystal surface modification; LPBF, laser powder bed fusion; DED, directed energy deposition; HDI, hetero-deformation-induced strengthening; YS, yield strength; UE, uniform elongation. The ductility-loss locations include both directly reported cases and mechanism-based risk regions associated with coarse/intergranular precipitates, carbide-rich zones, and localized martensitic transformation.

**Table 6:** Elemental design rules for Al, Ti, Si, C, B, and N additions in Fe-based MEAs.

Element	Benefits	Risks	Ideal Use
**Al** [16,93,96,101]	Promotes BCC/B2 formation, reduces density, expands the lattice, lowers VEC, and can assist ordered precipitation with Ni or Ti.	Excessive BCC/B2 formation, brittle intermetallics, or loss of FCC connectivity may reduce ductility and toughness.	Best for balanced FCC/BCC or FCC/B2 architectures that retain strain compatibility and continue hardening during deformation.
**Ti** [25,27,32,109]	Promotes L1_2_, B2, μ-phase, or TiC-related precipitation; strengthens interfaces; and can tune FCC stability through partitioning or TiC decomposition.	Coarse precipitates, brittle particles, or excessive austenite stabilization may suppress useful DIMT and weaken transformation-assisted hardening.	Best as a phase- and interface-control element in precipitation-strengthened, maraging/reversion, or TiC-assisted microstructures where retained hardening capacity is preserved.
**Si** [17,112]	Reduces Co/Ni demand, promotes FCC + BCC structures, activates Lüders deformation, enhances DIMT and HDI hardening, and supports low-cost Fe-rich design.	Excessive BCC fraction, localized Lüders deformation, or carbide-related brittle fracture in C-containing Si alloys may impair ductility.	Best in Fe-rich metastable systems where Si-induced FCC destabilization is paired with grain refinement, HDI hardening, or controlled DIMT.
**C** [12,18,46,116]	Provides interstitial strengthening, promotes carbide precipitation, modifies FCC stability, and can support TWIP- or TRIP-assisted deformation depending on matrix chemistry.	Excess carbides, carbide-rich brittle regions, particle/matrix decohesion, or loss of strain compatibility may reduce ductility.	Best when C remains compatible with an FCC-rich matrix or when fine carbides and controlled TRIP/TWIP operate together.
**B** [21,48,49]	Can strengthen interfaces or boundaries at trace levels and may assist sequential FCC twinning, FCC→HCP transformation, HCP twinning, and reverse transformation.	Excess B or boride formation may embrittle grain boundaries and reduce deformation compatibility.	Best at trace levels for boundary/interface regulation rather than as a major precipitation-forming addition.
**N** [19,20,52,53]	Provides strong interstitial strengthening, can promote local chemical order, may form nitrides or remain supersaturated, and can shift deformation toward TWIP/HDI hardening.	Nitride embrittlement, suppressed beneficial TRIP, or excessive local instability may reduce ductility and damage tolerance.	Best when solid-solution N or controlled nitride/LCO formation strengthens the FCC matrix without destroying uniform deformation.

Note: These rules are qualitative and matrix-dependent. The same element may either stabilize or destabilize FCC, promote or suppress DIMT, and strengthen or embrittle the alloy depending on base composition, SFE, phase fraction, segregation behavior, precipitate morphology, and processing history. FCC, face-centered cubic; BCC, body-centered cubic; VEC, valence electron concentration; DIMT, deformation-induced martensitic transformation; TRIP, transformation-induced plasticity; TWIP, twinning-induced plasticity; HDI, hetero-deformation-induced strengthening; LCO, local chemical order.

**Table 7:** Comparison between FCC→HCP TRIP and FCC→BCC DIMT in metastable Fe-based MEAs.

Comparison Item	FCC→HCP TRIP [6,11,123]	FCC→BCC/α’ DIMT [5,9,10]	Sequential FCC→HCP→BCC [6,124]
**Transformation path**	Metastable FCC transforms to ε-HCP during deformation.	Metastable FCC transforms to α’/BCC martensite during deformation.	FCC first transforms to HCP, followed by further transformation to α’/BCC martensite.
**Typical alloy family or system**	Low-SFE Fe-Mn-Cr-Ni/Co and Fe-Mn-Co-Cr MEAs; Si-modified low-SFE FCC alloys.	Fe-Co-Ni-Cr, Fe-Co-Cr-V/Ni, Fe-rich and Si-modified metastable FCC systems.	Highly metastable Fe-Co-Cr-V-Ni/Mn systems with strong transformation tendency.
**Temperature sensitivity**	Strongly promoted at cryogenic temperature because lower temperature reduces effective SFE and increases the driving force for ε-HCP formation.	Enhanced by cryogenic loading, Fe enrichment, reduced FCC stability, and accumulated plastic strain; can also occur during rolling or severe deformation.	Most evident at low temperature and high metastability, where both HCP and BCC/alpha martensite formation are activated.
**Hardening effect**	Introduces FCC/HCP interfaces, subdivides slip paths, cooperates with TWIP, and sustains work hardening when transformation is gradual.	Produces hard α/BCC martensite, fresh FCC/BCC interfaces, phase-stress partitioning, and strong transformation-assisted work hardening.	Provides multi-stage hardening by combining HCP interface formation with subsequent hard BCC/α martensite formation.
**Main ductility risk**	Excessive initial HCP or rapid FCC→HCP transformation can consume transformable FCC too early and reduce strain accommodation.	Early, excessive, or localized α/BCC formation can exhaust metastable FCC, concentrate stress/strain, reduce uniform elongation, and accelerate necking.	The sequence may become too rapid, leaving insufficient FCC for sustained plasticity and increasing premature localization risk.
**Typical characterization method**	XRD before/after deformation; EBSD phase maps; TEM observation of ε-martensite laths, stacking faults, and twins.	XRD phase analysis; EBSD phase maps; in situ neutron or synchrotron diffraction; magnetic measurements where applicable; post-deformation TEM.	Combined XRD, EBSD, and TEM; in situ diffraction preferred for tracking phase-fraction evolution and load partitioning.

Note: TRIP, transformation-induced plasticity; DIMT, deformation-induced martensitic transformation; α’ (or α), BCC martensite; FCC, face-centered cubic; HCP, hexagonal close-packed; BCC, body-centered cubic; SFE, stacking-fault energy. The distinction between pre-existing BCC and deformation-induced BCC/α martensite should be maintained when interpreting phase maps and post-deformation diffraction results.

**Table 8:** Summary of common precipitate types in Fe-based MEAs and related Fe-rich concentrated alloys.

Precipitate Type	Typical Morphology and Location	Main Strengthening or Matrix Effect	Potential Risk
L1_2_/γ′ precipitates [38,125,126]	Usually nanoscale, coherent or semi-coherent particles in the FCC matrix; can be spherical intragranular precipitates, rod-like grain-boundary precipitates, or lamellar/discontinuous precipitates depending on aging route and local composition.	Provide coherent precipitation strengthening, impede dislocation motion, promote dislocation storage, and can cooperate with heterogeneous grains, HDI strengthening, and TRIP when the FCC matrix remains metastable.	Coarsening, discontinuous grain-boundary precipitation, excessive volume fraction, or loss of matrix continuity may reduce ductility and strain compatibility.
B2 precipitates or B2 phase [127,128]	Can appear as nanoscale B2 precipitates within FCC or BCC regions, or as a B2-rich phase in FCC/BCC duplex or microlaminated structures; interface misfit depends strongly on the surrounding matrix.	Increase strength through ordered-particle strengthening, interface strengthening, and soft/hard phase partitioning; nearly coherent BCC/B2 interfaces can be especially effective.	Excessive B2 fraction, high interface misfit with FCC, or continuous brittle B2 networks may reduce ductility and toughness.
μ phase [14]	Usually Mo-rich or refractory-element-rich intermetallic precipitates; can form as intragranular nanoprecipitates after short annealing or as coarser intergranular precipitates after prolonged annealing.	Provides strong precipitation strengthening and can modify residual FCC matrix composition, thereby influencing FCC stability and DIMT kinetics.	Coarse μ phase or excessive intergranular precipitation may promote brittleness, reduce processability, and accelerate damage nucleation.
M_23_C_6_ carbides [18,46]	Typically Cr-rich carbides; may form at grain boundaries, phase boundaries, or within grains depending on C content and heat treatment.	Contribute to precipitation strengthening and can change residual matrix chemistry; Cr consumption may destabilize FCC and promote TRIP/DIMT in some matrices.	Cr depletion near carbides can impair corrosion resistance; coarse or continuous grain-boundary carbides may reduce ductility and promote intergranular damage.
M_6_C carbides [18]	Usually Mo-rich or Fe/Mo-rich carbides; often associated with Mo-containing Fe-based MEAs or CoCrFeNiMo-type systems.	Provide strong particle strengthening and can work with Mo solid-solution strengthening and matrix destabilization to enhance low-temperature hardening.	Coarse M_6_C particles may act as crack-initiation sites; excessive carbide formation may reduce matrix ductility and strain compatibility.
TiC particles [27]	TiC may appear as added ceramic reinforcement, in situ particles, or partially decomposed particles during additive manufacturing or high-temperature processing.	Can increase stiffness and strength through particle reinforcement; partial TiC decomposition may release Ti/C and alter γ-austenite stability, precipitation behavior, or DIMT tendency.	Coarse TiC particles, particle clustering, weak interfaces, or excessive austenite stabilization after TiC decomposition may weaken ductility and suppress beneficial transformation.
N-related structures [19,20,52,53]	Nitrides may form as nanoscale particles or local precipitates in N-containing alloys; in other cases, N may remain in solid solution or contribute to local chemical order instead of forming visible nitrides.	Can strengthen by precipitation, solid-solution/LCO effects, and defect interaction; may enhance HDI strengthening or modify TWIP/TRIP balance depending on matrix chemistry.	Nitride embrittlement, excessive local hardening, or suppression of beneficial TRIP may reduce ductility and deformation compatibility.
σ phase [75,129]	Usually Cr-rich or refractory-element-rich tetragonal intermetallic phase; can form during aging, annealing, or phase decomposition, often at boundaries or in chemically partitioned regions.	May increase strength by hard-phase reinforcement and grain-boundary pinning; in some thermomechanical routes, controlled σ-related structures may stabilize fine microstructures.	Generally brittle; excessive σ phase can reduce ductility, toughness, corrosion resistance, and strain compatibility.

Note: The listed effects are qualitative and matrix-dependent. The same precipitate type can be beneficial or detrimental depending on particle size, spacing, location, coherency, volume fraction, surrounding matrix stability, and processing history. References for representative examples are cited in the surrounding text rather than repeated in the table to avoid conflicts during reference-manager updates.

**Table 9:** Processing-property matrix for microstructural regulation in Fe-based MEAs.

Processing Route	Microstructural Signatures	Metastability Effects	Mechanical Benefits	Limitations or Risks
**Cold rolling/large thickness reduction** [140]	Shear bands, martensite variants, deformation texture, and γ→ε→α′ or γ→α′ transformation paths.	Activates strain-induced transformation and reveals transformation sequence under accumulated strain.	Increases work hardening and helps identify metastability-controlled deformation pathways.	Excessive reduction may cause strain localization, cracking, or rapid exhaustion of transformable FCC.
**Warm rolling and two-stage rolling** [141,142]	Enhanced dislocation slip, reduced cracking, refined grains, Σ3 twins, and controlled FCC→HCP transformation.	Suppresses premature transformation at moderate temperature while retaining transformation ability after optimized rolling/annealing.	Improves wrought-sheet processability and preserves strength–ductility balance.	Processing window is narrow; too low temperature may cause cracking, whereas too high temperature may suppress useful TRIP/TWIP.
**Multi-directional forging and annealing** [143]	Modified grain size, changed FCC/HCP phase fractions, and increased grain-boundary density.	Redistributes phase stability and changes the relative contributions of TRIP and TWIP.	Improves grain-boundary strengthening and transformation-assisted plasticity.	Process complexity and texture/phase heterogeneity may reduce reproducibility.
**Warm cross-rolling/critical recrystallization/partial recrystallization** [138,144]	Hierarchical nanotwins, retained defects, recrystallized and non-recrystallized domains, and deformation twins.	Delays or redistributes TRIP activation by controlling defect density and grain-scale heterogeneity.	Combines HDI strengthening, twin/dislocation storage, and transformation-assisted hardening.	Excessive soft–hard mismatch may concentrate strain at interfaces and trigger early damage.
**SLM processing of Si-added Fe-Mn-Co-Cr alloys** [145]	AM cellular structures, high dislocation density, Si-modified matrix, and TWIP/TRIP-active microstructure.	Introduces local metastability and printability-related heterogeneity through AM solidification and solute redistribution.	Improves strength–ductility balance through dislocation activity, TWIP/TRIP, and transformation-assisted hardening.	AM defects, segregation, anisotropy, and residual stress require control.
**DED processing** [146]	Mn-segregation-induced chemical boundaries, local SFE heterogeneity, and cellular structures.	Creates local metastability gradients that coordinate slip, TRIP, and TWIP.	Can improve ductility and strain hardening through chemically assisted deformation compatibility.	Chemical segregation may become harmful if it causes excessive local instability or cracking.
**LPBF and laser surface treatment** [88,147,148]	Cell-boundary segregation, post-AM cell restructuring, surface cellular structures, and localized defect networks.	Activates local TRIP/HDI coupling through cell-boundary chemistry and defect structures.	Enhances local deformation compatibility through cell-boundary chemistry, defect structures, back-stress hardening, and, in TRIP-sensitive systems, localized TRIP/HDI coupling.	Residual stress, lack-of-fusion defects, surface cracking, and unstable cell structures may reduce reliability.
**LPBF layered or VED-controlled FCC/BCC heterostructures** [149,150]	Periodically layered FCC-rich soft and BCC-rich hard regions; VED-controlled FCC/BCC phase fraction.	Uses process parameters to tune phase fraction, soft/hard contrast, and interface density.	Generates stress gradients, HDI strengthening, additional strain hardening, and printable high-elongation FCC/BCC heterostructures.	Strong process sensitivity; interlayer incompatibility, porosity, and phase imbalance may impair toughness and scalability.

Note: The table summarizes processing effects qualitatively. Representative references are listed in the processing-route column and further discussed in the surrounding text. The same processing route can be beneficial or detrimental depending on alloy chemistry, initial phase stability, deformation temperature, defect density, build quality, and post-processing history.

**Table 10:** Main strengthening mechanisms and secondary interactions in representative Fe-based MEAs.

Key Alloy/System	Main Mechanism	Secondary Interactions	Direct vs. Indirect Effects
**Fe-25Mn-5Co-12.5Cr-5Ni-2.5Si (in at.%)** [123]	Grain-boundary strengthening and intrinsic lattice resistance.	Boundary character affects slip transfer and deformation compatibility.	Direct strengthening arises from grain boundaries impeding dislocation motion and raising yield strength.
**Fe_50_Mn_30_Co_10_Cr_10_** [158]	Grain refinement and grain-boundary strengthening.	FCC→HCP transformation, reverse transformation, slip, and kink-band formation vary with grain size and annealing state.	Refined grains directly impede slip; grain size indirectly changes apparent stability and transformation kinetics.
**Co_18.5_Cr_12_Fe_55_Ni_9_Mo_3.5_C_2_** [13]	Carbide-related precipitation strengthening coupled with grain-size strengthening.	Carbide precipitation changes residual matrix chemistry, lowers ΔG_FCC→BCC_, and redistributes martensite nucleation sites.	Carbides and grain boundaries directly impede dislocations; matrix destabilization indirectly accelerates DIMT.
**Co_21_Cr_12.5_Fe_55_Ni_4_Mo_7.5_** [14]	Defect-architecture strengthening from retained non-recrystallized substructures, ultrafine grains, and high dislocation density.	Martensite interfaces and grain boundaries provide additional obstacles and strain-partitioning sites.	Stored defects and interfaces directly impede slip; heterogeneous domains secondarily sustain hardening beyond simple dislocation strengthening.
**DED-processed Fe_60_(CoNi)_30_Cr_10_** [63]	Cellular defect architecture and HDI strengthening.	Cellular structures increase KAM, LAGB fraction, dislocation density, and local strain gradients.	Dislocations and cell boundaries directly impede slip; GND accumulation and back stress indirectly enhance HDI strengthening.
**Mo-added Fe-Mn-Al-Ni-C** [153]	Mo-rich M_6_C carbide strengthening and Mo solid-solution strengthening.	Non-shearable carbides and Mo in solution also promote back-stress hardening.	Carbides and Mo solutes directly impede dislocation motion; heterogeneous deformation secondarily contributes to back stress.
**B-doped Fe_60_Co_15_Ni_15_Cr_10_** [155]	Matrix-destabilization-assisted TRIP strengthening rather than conventional boride precipitation strengthening.	Cr_2_B formation depletes Cr from the FCC matrix and enhances room-temperature TRIP.	Direct Cr_2_B precipitation strengthening is small; Cr depletion indirectly destabilizes FCC and increases transformation-assisted hardening.
**W-containing Fe_64_(CoCrNi)_36_** [154]	W solid-solution strengthening at lower W contents and hard-precipitate strengthening when W partitions at higher W contents.	The ductile FCC matrix is required to arrest microcracks generated near hard precipitates.	W solutes and W-rich precipitates directly strengthen the matrix; matrix ductility indirectly controls damage tolerance.

Note: Direct strengthening refers to obstacles that directly resist dislocation motion, such as grain boundaries, stored dislocations, interfaces, solute atoms, and precipitates. Indirect effects modify matrix stability, transformation kinetics, strain partitioning, or damage tolerance. References for representative examples are cited in the surrounding text rather than repeated in the table to avoid conflicts during reference-manager updates.

**Table 11:** Service-related performance and qualification implications of representative Fe-based MEAs.

Alloy/System	Service Aspect	Key Observation and Design Implication
**Cost-effective Fe-Co-Ni-Cr-Si-Al ferrous MEA** [36]	Cryogenic tensile/Charpy impact	Strength increases at 77 K, but Charpy absorbed energy decreases from 95.5 J at 298 K to 32.2 J at 77 K; tensile strength does not guarantee cryogenic toughness.
**Fe_40_Mn_40_Co_10_Cr_10_** [37]	UNSM-treated fatigue	UNSM increases YS by more than 70%, but accelerates fatigue-crack initiation and reduces crack-growth resistance; surface strengthening must be assessed with fatigue-damage tolerance.
**Fe_50−x_Mn_30_Co_10_Cr_10_Si_x_** [168]	Low-cycle fatigue	At 0.7% strain amplitude, up to 4 at.% Si changes monotonic tension only slightly but strongly affects cycling; fatigue life exceeds 10,000 cycles.
**Fe_49.5_Mn_25_Cr_15_Ni_10_C_0.5_** [12]	Cryogenic tension + corrosion	The MEA gives the highest UTS-ductility product at 77 K among compared alloys, but corrosion ranking differs from mechanical ranking.
**Fe_50_Mn_25_Cr_15_Ni_10−x_Co_x_** [7]	Cryogenic ductility + corrosion	Co-for-Ni substitution improves room-temperature strength but reduces cryogenic ductility and corrosion resistance as HCP fraction increases.
**Fe_61.5_Cr_17.5_Ni_13_Al_8_** [169]	NaCl and H_2_SO_4_ corrosion	Corrosion resistance is associated with Fe/Cr oxide-hydroxide passive films; passive-film chemistry is a key service variable.
**FeMnCoCr + small Cu** [170]	NaCl corrosion + mechanical response	Small Cu additions improve strength–ductility and NaCl corrosion resistance by stabilizing the matrix and compacting the passive film.
**GTA-welded Fe_60_Co_15_Ni_15_Cr_10_** [171]	Welding response	Weld metal contains FCC1 + Cu-rich FCC2; BM/HAZ shows DIMT and weld metal shows twinning; HAZ, residual stress, and local transformation require service qualification.
**L1_2_-strengthened Fe_49.6_Ni_28_Al_6_Ti_3_Zr_0.1_C_0.2_B_0.1_Cr_13_** [38]	Wide-temperature response	Shows strength–ductility retention from cryogenic to elevated temperatures with corrosion-resistant L1_2_-strengthened design; multi-temperature/multi-property evaluation is needed.

Note: RT, room temperature; LNT, liquid-nitrogen temperature; YS, yield strength; UTS, ultimate tensile strength; UE, uniform elongation; HCF, high-cycle fatigue; LCF, low-cycle fatigue; UNSM, ultrasonic nanocrystal surface modification; GTA, gas tungsten arc welding; BM, base metal; HAZ, heat-affected zone.

**Table 12:** Service-property checklist for major Fe-based MEA families.

Alloy Family/Design Route	Relatively Well-Studied Properties	Underreported or Missing Validation	Key Environments to Separate
**Fe-Co-Ni-Cr and Fe-rich FCC/BCC metastable systems** [5,10,36,40]	RT and cryogenic tensile properties; FCC→BCC DIMT; phase-stress partitioning; selected cryogenic impact or Charpy response.	Underreported: fatigue response, fatigue-crack growth, crack initiation after surface strengthening, weld-zone deformation, and dimensional stability. Missing/high-priority: fracture toughness, corrosion-fatigue, hydrogen embrittlement, long-term aging, creep, and weld-joint efficiency.	Room temperature vs. 77 K; uniaxial vs. multiaxial loading; base metal vs. HAZ/weld metal; air vs. corrosive media.
**Fe-Mn-Cr-Ni/Co low-SFE TRIP/TWIP systems** [7,12,37,168,172]	RT and cryogenic tensile properties; FCC→HCP TRIP; TWIP/TRIP competition; selected low-cycle fatigue and corrosion comparisons.	Underreported: high-cycle fatigue, fatigue-crack growth, fracture toughness, corrosion-fatigue, forming reliability, and cryogenic impact toughness. Missing/high-priority: hydrogen effects, long-term aging, weldability, creep/stress relaxation, and combined cyclic-corrosive service behavior.	Room temperature vs. cryogenic temperature; monotonic vs. cyclic loading; chloride solution vs. air; low vs. high strain amplitude.
**Fe-Co-Cr-Ni-Mo/carbide- or μ-strengthened systems** [13,14,18,65]	RT and cryogenic tensile response; precipitation strengthening; Mo-assisted solid-solution strengthening; matrix destabilization and DIMT effects.	Underreported: corrosion behavior, passive-film durability, carbide/μ-phase stability, damage tolerance, and fracture resistance. Missing/high-priority: creep, long-term aging, hydrogen embrittlement, corrosion-fatigue, and toughness after thermal exposure.	Cryogenic vs. elevated-temperature service; chloride-containing environments; aged vs. solution-treated states.
**Al/Ti/Si-modified and L1_2_/B2-strengthened systems** [38,99,168,169]	Strength–ductility retention over a wide temperature range; precipitation-assisted strengthening; selected corrosion-resistant L1_2_-strengthened designs; Si-related cyclic behavior.	Underreported: fatigue-crack growth, fracture toughness, impact toughness, coarsening resistance, weldability, and forming behavior. Missing/high-priority: creep resistance, long-term precipitate stability, corrosion-fatigue, hydrogen effects, and thermal-cycling durability.	Cryogenic, room-temperature, and elevated-temperature conditions; aged vs. overaged states; dry vs. corrosive environments.
**C/B/N and interstitially modified systems** [12,52,53,155,167]	Cryogenic tensile response; matrix stabilization or destabilization; selected corrosion and irradiation stability in C-containing systems.	Underreported: fatigue, toughness, carbide/nitride stability, corrosion-fatigue, and local embrittlement caused by interstitial segregation. Missing/high-priority: hydrogen embrittlement, long-term aging, creep, weldability, and combined irradiation-corrosion-mechanical response.	Cryogenic tension; chloride corrosion; irradiation/thermal exposure; hydrogen-containing environments.
**Surface-modified, AM-processed, and welded Fe-based MEAs** [37,63,147,148,171]	Surface strengthening; AM cell structures; weld-metal/HAZ phase response; residual microstructural heterogeneity; selected fatigue or welding examples.	Underreported: standardized fatigue resistance, residual-stress effects, defect tolerance, local corrosion, and HAZ stability. Missing/high-priority: weld-joint efficiency, fatigue-crack growth, corrosion-fatigue, hydrogen-assisted cracking, long-term aging of AM cells, and creep of printed/welded components.	As-built vs. post-treated AM states; base metal vs. weld metal/HAZ; room-temperature vs. cryogenic service; air vs. corrosive/hydrogen media.

Note: “Relatively well-studied” means that multiple tensile or service-related reports exist for that alloy family, not that the property is fully qualified for engineering use. “Underreported” indicates scattered or system-specific evidence. “Missing/high-priority validation” indicates essential structural-qualification properties that remain insufficiently tested. Representative references are listed in the alloy-family column and further discussed in the surrounding text.

**Table 13:** Numbered challenge checklist for Fe-based MEAs.

Challenge	Why It Matters	Current Evidence Gap	Recommended Test or Modeling Approach
**1. Cost-performance and critical-element reduction** [1,17,36]	Fe enrichment and Co/Ni reduction are attractive only if strength–ductility balance, corrosion resistance, processability, and reproducibility are retained. A nominally low-cost alloy is not necessarily application-ready.	Many studies report tensile properties, but fewer report price-normalized strength, density-normalized strength, critical-element usage, processing yield, post-processing cost, and service-property trade-offs.	Establish cost-property maps combining alloy price, density, critical-element content, processing route, heat-treatment cost, tensile properties, toughness, corrosion resistance, and weldability. Combine CALPHAD/ICME screening with validation on reproducible heats.
**2. Local metastability and transformation kinetics** [9,10,40,151,157,163]	TRIP/TWIP/DIMT can improve strain hardening, but uncontrolled transformation can cause early FCC exhaustion, strain localization, and embrittlement. The useful condition is gradual and spatially distributed transformation, not maximum martensite fraction.	A single SFE value or room-temperature tensile curve is often used to judge stability, whereas local chemistry, short-range order, temperature, strain rate, grain size, stress state, and initial phase fraction are not systematically mapped.	Build deformation-mechanism maps across temperature, strain rate, stress state, grain size, and initial phase fraction. Use in situ XRD/neutron/synchrotron diffraction, EBSD-DIC, TEM, local strain mapping, and crystal-plasticity/phase-field modeling to quantify transformation onset, rate, and spatial distribution.
**3. Quantitative separation of strengthening mechanisms** [13,63,153,154,155]	Solute strengthening, grain refinement, retained defects, precipitation, HDI strengthening, TRIP, and TWIP usually operate together. Without mechanism separation, composition design can become empirical and difficult to transfer across alloy families.	Many examples merge multiple mechanisms without identifying the dominant contribution or distinguishing direct strengthening from indirect effects such as matrix destabilization or martensite-nucleation-site redistribution.	Combine strengthening decomposition with in situ diffraction, dislocation-density measurement, precipitate statistics, local strain mapping, and constitutive modeling. Separate direct effects such as solute, particle, grain-boundary, dislocation, and interface strengthening from indirect effects such as altered SFE, ΔG, transformation kinetics, and damage tolerance.
**4. Service-property validation beyond tensile tests** [12,36,37,168,169,171]	High tensile strength and elongation do not guarantee fatigue resistance, fracture toughness, impact toughness, corrosion durability, hydrogen tolerance, creep resistance, weldability, or long-term stability.	Existing datasets are rich in room-temperature and cryogenic tensile curves but sparse in fatigue-crack growth, fracture toughness, corrosion-fatigue, hydrogen embrittlement, creep/stress relaxation, weld-joint efficiency, HAZ stability, and long-term aging.	Establish service-property matrices for room-temperature, cryogenic, elevated-temperature, corrosive, hydrogen-containing, and irradiation environments. Required tests include HCF/LCF, fatigue-crack growth, Charpy/KIC/JIC or CTOD toughness, corrosion and corrosion-fatigue, hydrogen charging/slow-strain-rate tests, creep/stress relaxation, and long-term aging.
**5. Processing, joining, AM, and scale-up reproducibility** [63,140,143,148,171]	Real components require rolling, forging, forming, additive manufacturing, welding, and post-processing. These processes can alter phase stability, residual stress, segregation, defects, precipitates, and HAZ behavior.	Many alloys are tested as small laboratory samples, while fewer studies verify large plates, printed parts, welded joints, processing windows, residual-stress control, defect tolerance, and heat-to-heat reproducibility.	Develop process-structure-property maps for wrought, AM, surface-modified, and welded states. Use weldability trials, HAZ characterization, residual-stress measurement, AM defect statistics, post-processing optimization, and component-scale validation to compare Fe-based MEAs with steels, Ni-based alloys, and conventional cryogenic alloys.

Note: The cited references after each challenge provide representative published examples related to the corresponding issue. They support the existence of relevant alloy-design, metastability, strengthening, service-property, processing, AM, or welding cases, but they do not imply that every cited study covers all evidence gaps or recommended validation approaches listed in the same row. The evidence gaps and recommended approaches are author-synthesized conclusions based on the current state of the literature.

**Table 14:** Time-staged future priorities and concrete deliverables for Fe-based MEAs.

Timeframe	Priority	Concrete Deliverables	Recommended Actions
**Near-term** **(1–2 years)**	**1. Build comparable open datasets**	Open datasets: composition, processing, heat treatment, phase constitution, grain size, recrystallized fraction, precipitate state, tensile and available service data.	Standardize reporting; disclose temperature, strain rate, geometry, raw stress–strain data, and processing history; extend high-throughput DED/composition-gradient studies to fatigue, corrosion, thermal stability, and local fracture indicators.
**Near-term** **(1–2 years)**	**2. Define benchmark alloy families**	Benchmark Fe-rich FCC, FCC/HCP TRIP, FCC/BCC DIMT, interstitially tuned, and precipitation-assisted Fe-based MEAs.	Select compositionally related alloy series rather than isolated record compositions; repeat tests across laboratories to evaluate reproducibility and heat-to-heat sensitivity.
**Near-term** **(1–2 years)**	**3. Establish standard heat treatments and processing histories**	Standard solution treatments, aging conditions, cold/warm rolling schedules, recrystallization states, AM post-treatments, and welding/post-weld heat treatments.	Report processing windows needed to reproduce phase fraction, defect density, precipitate distribution, texture, segregation state, residual stress, and weld/HAZ microstructures.
**Mid-term** **(3–5 years)**	**4. Construct multi-property service matrices**	Property matrices covering room-temperature, cryogenic, elevated-temperature, corrosive, hydrogen-containing, irradiated, and welded conditions.	Go beyond YS, UTS, and elongation; evaluate fatigue life, fatigue-crack growth, fracture toughness, impact toughness, creep/stress relaxation, corrosion, corrosion-fatigue, hydrogen embrittlement, weld-joint efficiency, formability, and aging stability.
**Mid-term** **(3–5 years)**	**5. Build mechanism maps for metastability control**	Deformation-mechanism maps linking composition, local chemistry, SFE/ΔG, grain size, phase fraction, strain rate, temperature, and stress state.	Use in situ XRD, neutron/synchrotron diffraction, EBSD-DIC, TEM, and local strain mapping to quantify transformation onset/rate/saturation, twin activity, phase-stress partitioning, and damage initiation.
**Mid-term** **(3–5 years)**	**6. Integrate manufacturability into alloy design**	Processing–structure–property maps for wrought, AM, surface-modified, and welded Fe-based MEAs.	Treat printability, hot cracking, lack-of-fusion defects, rolling/forming response, residual stress, segregation, heat-treatment tolerance, weldability, and HAZ stability as design variables; validate plates, printed parts, and welded joints.
**Long-term** **(5+ years)**	**7. Develop validated constitutive and damage models**	Validated crystal-plasticity, phase-field, transformation-kinetics, and damage models supported by in situ experiments and service tests.	Link CALPHAD and first-principles calculations with crystal-plasticity/phase-field models to predict local metastability, strain partitioning, TRIP/TWIP activation, precipitate evolution, crack initiation, hydrogen-assisted damage, creep, and weld-HAZ degradation.
**Long-term** **(5+ years)**	**8. Establish certifiable Fe-based MEA families**	Reproducible alloy families with validated processing windows, standard heat treatments, service-property matrices, and component-level data.	Shift from isolated record compositions to scalable, certifiable alloy series; benchmark Fe-based MEAs against steels, Ni-based alloys, and conventional cryogenic alloys using identical manufacturing and service-qualification criteria.

Note: The proposed timeframes are indicative. Near-term priorities emphasize data comparability and reproducibility; mid-term priorities emphasize multi-property qualification and mechanism mapping; long-term priorities emphasize predictive modeling and certifiable alloy families. References for representative examples are cited in the surrounding text rather than repeated in the table.

## Data Availability

No new data were created or analyzed in this study. Data sharing is not applicable to this article.
